# Genome-Wide Identification of Expansin Gene Family and Their Response under Hormone Exposure in *Ginkgo biloba* L.

**DOI:** 10.3390/ijms24065901

**Published:** 2023-03-21

**Authors:** Fangyun Guo, Jing Guo, Yousry A. El-Kassaby, Guibin Wang

**Affiliations:** 1 College of Forestry, Nanjing Forestry University, Nanjing 210037, China; 2 Co-Innovation Center for Sustainable Forestry in Southern China, Nanjing Forestry University, Nanjing 210037, China; 3 Department of Forest and Conservation Sciences, Faculty of Forestry, The University of British Columbia, Vancouver, BC V6T 1Z4, Canada

**Keywords:** *Ginkgo biloba*, expansins (*GbEXPs*) identification, tissue development, exogenous phytohormones

## Abstract

Expansins are pH-dependent enzymatic proteins that irreversibly and continuously facilitate cell-wall loosening and extension. The identification and comprehensive analysis of *Ginkgo biloba* expansins *(GbEXPs*) are still lacking. Here, we identified and investigated 46 *GbEXPs* in *Ginkgo biloba*. All *GbEXPs* were grouped into four subgroups based on phylogeny. *GbEXPA31* was cloned and subjected to a subcellular localization assay to verify our identification. The conserved motifs, gene organization, cis-elements, and Gene Ontology (GO) annotation were predicted to better understand the functional characteristics of *GbEXPs*. The collinearity test indicated segmental duplication dominated the expansion of the *GbEXPA* subgroup, and seven paralogous pairs underwent strong positive selection during expansion. A majority of *GbEXPAs* were mainly expressed in developing Ginkgo kernels or fruits in transcriptome and real-time quantitative PCR (qRT-PCR). Furthermore, *GbEXLA4*, *GbEXLA5*, *GbEXPA5*, *GbEXPA6*, *GbEXPA8*, and *GbEXPA24* were inhibited under the exposure of abiotic stresses (UV-B and drought) and plant hormones (ABA, SA, and BR). In general, this study expanded our understanding for expansins in Ginkgo tissues’ growth and development and provided a new basis for studying *GbEXPs* in response to exogenous phytohormones.

## 1. Introduction

Cell-wall plasticity is a key determinant in plant growth, development, and response to diverse environments [1]. To grow, plant cells can change their shape or structure in a short time to transform themselves into various differentiated cells to meet current plant-growth needs and adapt to a specific growth environment [1]. Cell-wall loosening is a prerequisite for the remodeling of the cell wall, in which new structural components are added to the cell wall or altering its physical structure, inducing anisotropic growth and shape changes in the cell [2]. Modifying proteins attached to the cell wall play an integral role in cell-wall relaxation, the most recognized of which are expansins (EXPs) [3]. As the primary wall-loosening factor, EXPs can regulate wall relaxation directly without any chemical energy by means of auxin activity, and this relaxation of stress on the cell wall induces a bulking turgor-driven cell expansion [1,4].

Therefore, EXPs constitute a class of proteins that contribute to cell-wall extension [5,6]. It has been proposed that plant EXPs function in breaking hydrogen bonds between cell-wall polymers made of cellulose and hemicellulose, allowing the polymers to become movable and rearrangeable to produce new expansive growth [7,8]. This growth hypothesis proposed that EXPs promote cell-wall elongation since they are acid-induced proteins [9]. Cellulose polysaccharides constitute the primary cell-wall polymer. H^+^-ATP, which is released from the plasma membrane, lowers the pH around the cell wall [10], inducing EXPs to play their roles, and short polysaccharide fragments are released from the cell-wall polymer cellulose microfibrils, relieving the tension and, by loosening the binding between the cellulose microfibrils and the polysaccharide [11], allowing sliding among microfibrils [12]. The newly synthesized cellulose, hemicellulose, and pectin are filled and precipitated into the cell wall to maintain the thickness of the cell wall, enhancing its strength and toughness during the new growth processes [13].

Based on the above knowledge, it is believed that EXPs are the main determinant in cell shape for many developmental processes, such as their regulation in cell-wall extensibility [7,8,14], including expansion and elongation [15]. Since the discovery of *EXPs* in cucumber [10], their functions have been reported successively in other species, such as promoting the elongation of roots in soybean [16] and stems in rice [17], contributing to strawberry green fruits growth [18] and loquat mature fruits softening [19], and triggering rose petal and fruits abscission [20]. The overexpression of *OSEXPA8* affected rice height, leaf number, and root development [21]. In aspen, *PttEXPA1* transgenic lines showed increased stem internode elongation, leaf expansion, and larger cell sizes in leaves [22]. Wheat *TaEXPA2* and *TaEXPB23* improved oxidative stress tolerance in transgenic Arabidopsis and tobacco, respectively [23,24]. Thus, it is generally concluded that EXPs mediate multiple biological functions in growth and development and stress response in most plants. These functions depend on two conserved domains, namely (1) double-psi beta-barrel (DPBB), which comprises more than 120 amino acids with a His-Phe-Asp (HFD) motif and conserved cysteine residues (N-terminal); and (2) Pollen_allerg, which contains polar and aromatic amino acid residues that are conducive to binding polysaccharides (C-terminal) [25]. Almost all *EXPs* contain these two conserved domains, yet little is known about the molecular regulatory functions of *Ginkgo biloba EXPs*.

As typical gymnosperm, *G. biloba* L., is a large ornamental tree that is tolerant to adverse growing conditions [26]; the species is 150 million years old [26], and the leaves’ bright color and unique shape are truly graceful. Its leaves and seeds have medicinal and edible values [27]. The species’ extraordinary growth performances have attracted scholars’ attention; the mutant *G. biloba* golden leaves had fewer and smaller chloroplasts that were abnormally developed [28]. A pharmacologically active *G. biloba* extract had terpenoids synthesized mainly in the roots [29]. The vascular cambium in nonaging Ginkgo was closely related to its long lifespan [30]. However, the achievement for this growth phenomena was based largely on the plasticity of the cell wall, and mining the excellent genes that play an important role in the growth and development and environmental responses of ancient *G. biloba* can provide a reference in learning more about this species. Herein, we systematically identified 46 *GbEXPs* based on the recent *G. biloba* genome sequence release; *GbEXPA31* was further selected for cloning to verify our identification. The functional properties of *GbEXPs* were investigated using multiple bioinformatics analyses, including phylogeny, gene organization, motifs, regulatory elements, GO annotation, and collinearity. Transcriptome profiles combined with qRT-PCR trials investigated the *GbEXPs* expression pattern in developing Ginkgo tissues and exposure of abiotic stresses and plant hormones. This work established the basis for Ginkgo *GbEXPs* study and gave new research perspective in *GbEXPs* responding to diverse conditions.

## 2. Results

### 2.1. GbEXP Genes Identification and Phylogenetic Relationships

Ginkgo *EXPs* were identified by employing local Blastp and online tools. A total of 46 *GbEXP* genes (*GbEXPs*) were designated according to Arabidopsis *AtEXP* classification and *GbEXP* chromosomal position. These 46 *GbEXP* genes were divided into four subgroups, namely *GbEXPA, GbEXPB*, *GbEXLA*, and *GbEXLB*, with 32, 4, 5, and 5 genes, respectively (see Supplementary Table S1 for their general characteristics). GbEXPA24 encoded the shortest putative protein of 231 amino acids; the molecular weight (MW) ranged from 25,010.84 kDa (GbEXPA31) to 41,674.53 kDa (GbEXLB4), with the isoelectric point (pI) ranging from 4.61 (GbEXPA16) to 9.98 (GbEXPA20). Only GbEXPA2, GbEXPA18, and GbEXLB4 exhibited unstable amino acid properties; their instability indexes were greater than 40. Overall, 21 and 16 of 46 GbEXPs might function in the chloroplast and extracellular, respectively.

**Figure 1:**
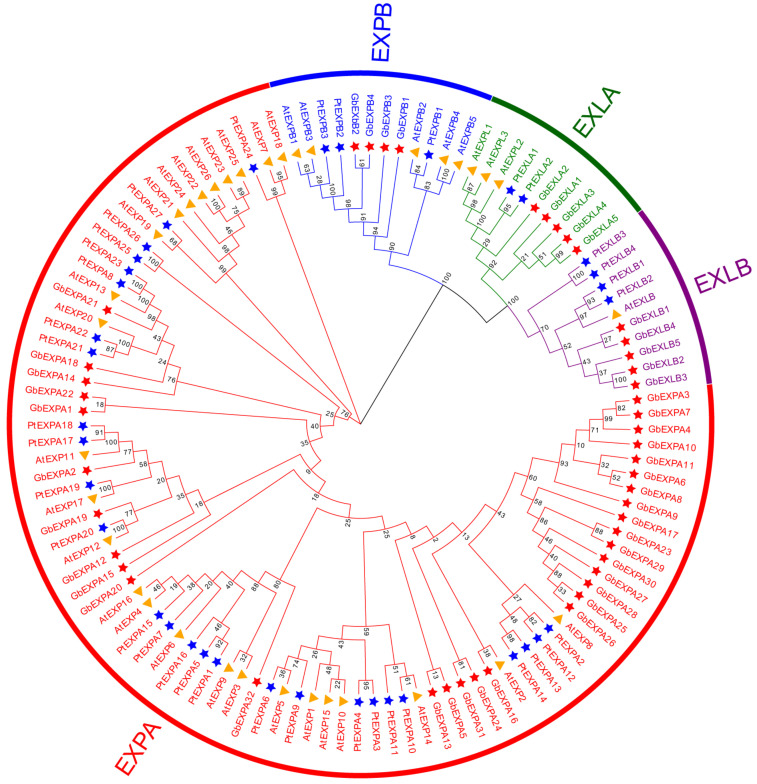
Bayesian phylogenetic tree of all expansin proteins from *Ginkgo biloba*, *Arabidopsis thaliana*, and *Populus trichocarpa*. Clades in red, blue, purple, and orange branches refer to the *EXPA*, *EXPB*, *EXLA*, and *EXLB* subfamilies, respectively. *EXP* genes are marked by orange triangles, blue stars, and red stars for *Arabidopsis*, *Populus trichocarpa*, and *Ginkgo biloba*, respectively.

The phylogenetic relationships among Ginkgo, *Arabidopsis*, and poplar were determined with 46 GbEXPs, 35 AtEXPs [31,32], and 36 PtEXPs [33] (File S1). *EXPA* was the largest subgroup containing 33 *GbEXPs*, and *GbEXPA31* was assigned to this subgroup, which is located in the phylogenetic branch adjacent to *GbEXPA25* and *GbEXPA5* (Figure 1). *PtEXPA14*, *PtEXPA13*, *PtEXPA12*, *AtEXP14*, and *AtEXP2* had higher homology with *GbEXPA31* in all *EXPs* among the three species. *GbEXPB* was the least abundant subgroup, with only four members.

### 2.2. GbEXPA31 Cloning and Characteristic Analysis

The open reading frame (ORF) of *GbEXPA31* was 705 bp and encoded a total of 234 amino acids (Supplementary Figure S3A). It was mainly composed of a random coil (61.97%), extended strand (31.20%), and alpha helix (6.84%) (Supplementary Figure S3B), and the simulated 3D structure verified this prediction (Figure S3C). As the hydrophilic scores in most amino acids were negative, GbEXPA31 may function as a hydrophilic protein (Supplementary Figure S3D). Six conserved motifs (Boxes I–VI) were detected (Figure S3E), and two cysteine-rich regions (C-rich), Box I–II at the N-terminal, and Box III HFD were typically characteristic residues in *EXPs* [33]. Box VI enriched tryptophans (W) at the N-terminal of EXPs. The W residues were involved in cellulose-binding-like domain [33]. These special amino acid residues may be beneficial for *EXPs* to form specific 3D structures to perform their function of binding microfibrils and determining cell walls’ shapes [34,35].

### 2.3. GbEXPA31 Subcellular Localization

**Figure 2:**
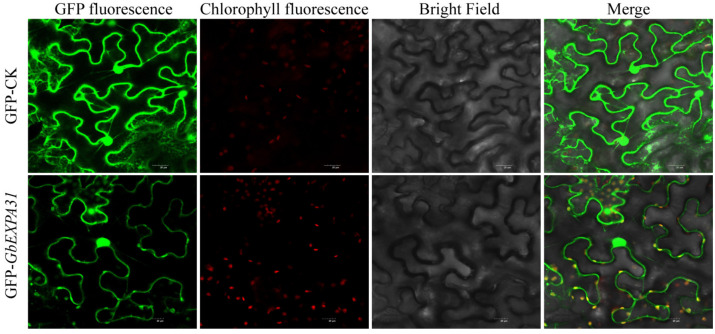
GbEXPA31 subcellular localization. GFP-CK: GFP blank vector, transient expression of *GbEXPA31* fusing GFP in tobacco (Nicotiana benthamiana) epidermal cells by confocal laser microscopy. Scale bars = 20 µm.

The full-length coding sequence of *GbEXPA31* was fused with the Green fluorescent protein (EGFP) at the C terminal under the driver of the 35S cauliflower mosaic virus (CaMV) promoter in the pART-CAM-EGFP backbone vector; a blank vector encoding free GFP was embedded in the same manner. The test group exhibited an additional GFP signal, and more red particles than the control were observed in GFP fluorescence and chloroplast channels. The merged channel for GFP, chlorophyll, and bright field also showed abundant gold particles in the chloroplast. These results revealed that GbEXPA31 might be located in the chloroplast, nucleus, plasma membrane, and cell wall (Figure 2).

### 2.4. Conserved Motifs, Gene Structures, Cis-Regulatory Elements, and Chromosome Mapping Analysis

**Figure 3:**
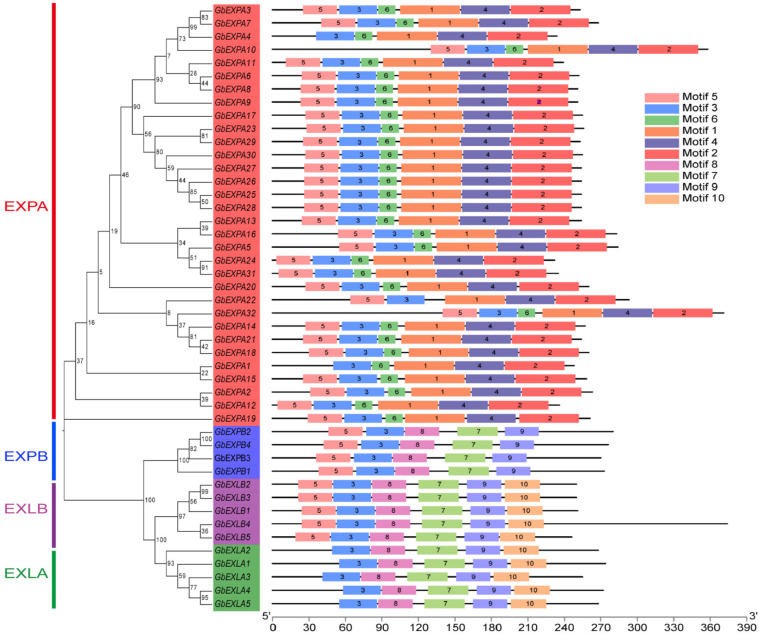
The phylogenetic tree and motif distribution of the *GbEXP* family.

**Figure 4:**
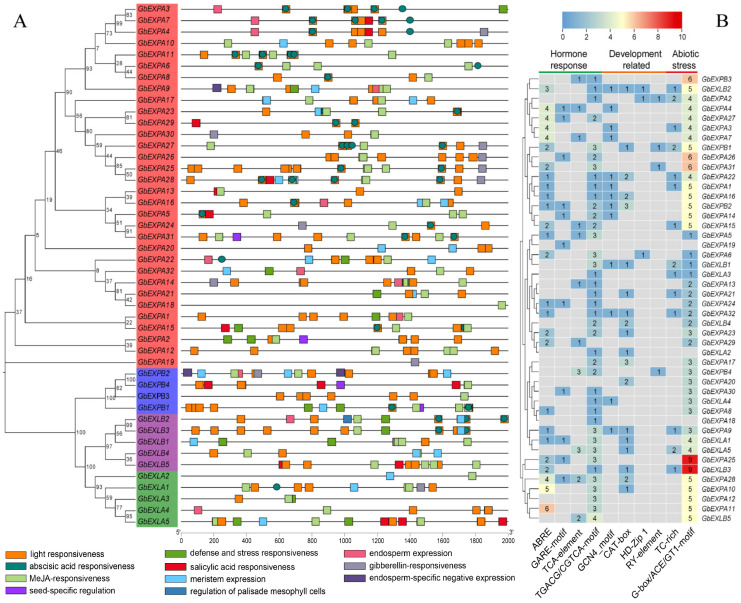
Number of regulatory elements related to plant hormone response, development, and abiotic stress. (**A**) Phylogeny and distribution of cis-elements in *GbEXPs* (**B**) A heatmap showing the counts of cis-acting elements in the promoters of *GbEXPs*.

To further investigate the conservation and variation of *GbEXPs*, the conserved motifs were predicted, and ten types of motifs in *GbEXPs* were found (see Figure 3 and Supplementary Figure S4 for details). The type, number, and order of these motifs in the four subgroups (*GbEXPA*, *GbEXPB*, *GbEXLA*, and *GbEXLB*) corresponded to their phylogenetic clustering. Generally, the *GbEXPA* subgroup possessed Motifs 5, 3, 6, 1, 4, and 2; *GbEXPB* and *GbEXLB* subgroups contained Motifs 5, 3, 8, 7, and 9; and the *GbEXLA* subgroup lacked Motif 5. This finding verified that *GbEXLA* and *GbEXLB* subgroups had a close evolutionary relationship. *GbEXLB3* had the longest intron sequence, followed by *GbEXPA7,* and their intron sequences were much longer than those of the other two subgroups (Supplementary Figure S5). No significant differences were found in the cis-acting elements related to abiotic stress, growth and development, and phytohormone responses among the four *GbEXP* subgroups (Figure 4A). Notably, *GbEXPA25* and *GbEXLB3* contained nine elements involved in light responsiveness, and they were the most common in all elements (Figure 4B and Table S3). *GbEXPA31*, *GbEXPA2*, *GbEXPB4*, and *GbEXPB1* contained one seed-specific regulatory element (RY-element) (Table S3). Moreover, *GbEXLA5* contained three salicylic acid (SA)-responsive elements, and this may reflect its role in responding to the SA treatment. Collectively, most *GbEXPs* contained more cis-elements related to plant hormone and abiotic stress responses than were related to development. The motifs and cis-elements results indicated that several *GbEXPs* may have a conserved function.

The 46 *GbEXPs* were unevenly distributed on 10 out of the Gingko’s 12 chromosomes (Supplementary Figure S6). Chr1 and chr11 harbored more *GbEXPs* than the other chromosomes, with 12 and 11 members, respectively. Among them, 22 of 23 belonged to the *EXPA* subgroup, including *GbEXPA31*, and were located in the 3′ region of ch1 (*GbEXPA1-11*) and the 5′ region of ch11 (*GbEXPA22-32*), implying that gene expansion has occurred in the *EXPA* subgroup [36]. There were also few *GbEXPs* located on different chromosomes, such as *GbEXPA12* on chr3, *GbEXPA13-14* on chr4, and *GbEXPA15* on chr7. Moreover, the sparse distribution was significantly different from *GbEXPA1-11* and *GbEXPA22-32*. Therefore, their gene structures and expression characteristics needed to be deeply analyzed and interpreted.

### 2.5. Gene Ontology (GO) and Collinearity Analyses

**Figure 5:**
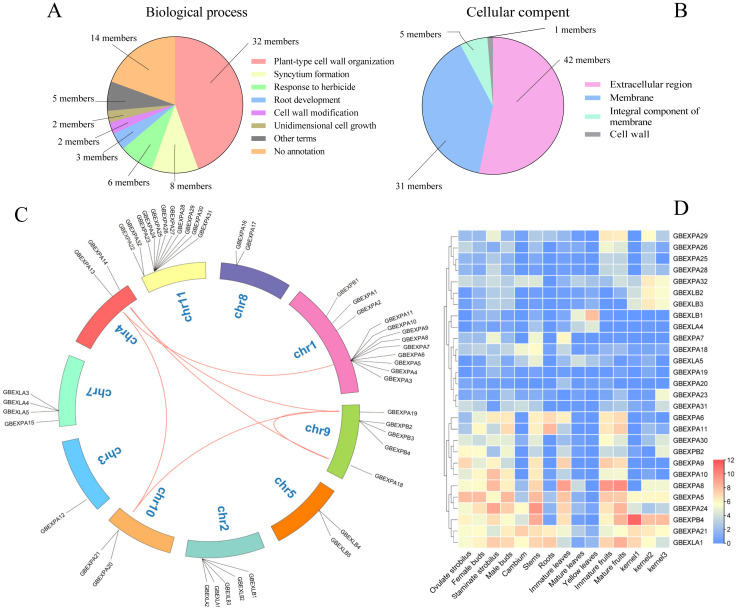
GO annotation, syntenic analysis, and tissue-specific expression of the *GbEXP* family. (**A**) Biological process terms of *GbEXPs*. (**B**) Cellular component terms of *GbEXPs*. (**C**) Collinearity analysis of *GbEXPs*. (**D**) Tissue-specific expression of *GbEXPs* in the transcriptome.

All *GbEXPAs* were annotated to the term of plant-type cell-wall organization; eight and six were involved in syncytium formation and herbicide response, respectively (Figure 5A and Table S4). A total of three, two, and two *GbEXPAs* were involved in root development, cell-wall modification, and unidimensional cell growth, respectively. None of the *GbEXPB*, *GbEXLA*, or *GbEXLB* genes were assigned to any known biological processes. Among the cellular components, 31 genes were located in both extracellular and membrane regions (Figure 5B); only *GbEXPA14* was involved in the cell wall. Moreover, none of the *GbEXPs* were annotated to a molecular functional term. Such findings indicated that the molecular and biological functions of *GbEXPs* needed further investigation, especially the *EXPB*, *EXLA*, and *EXLB* subgroups.

We found 16 duplicated paralogs in the *GbEXPA* subgroup according to their location in syntenic blocks (Figure 5C and Table 1). Seven duplicated pairs (*GbEXPA13/3, GbEXPA13/7*, *GbEXPA13/10*, *GbEXPA13/21*, *GbEXPA19/20*, *GbEXPA18/19*, and *GBEXPA14/19*) were subject to positive selection, as their Ka/Ks ratios were greater than 1, and the ratios of *GbEXPA13/3* and *GbEXPA13/7* were greater than 2, indicating that these three genes (*GbEXPA13*, *GbEXPA3*, and *GbEXPA7*) underwent strong evolutionary divergence. These results partially supported the previous assumption that two gene clusters are located on chr 1(*GbEXPA1-11*) and chr11(*GbEXPA22-32*). These gene pairs are in segmental duplication and may have redundant biological functions.

### 2.6. Expression Pattern of GbEXPs in G. biloba Tissues

**Figure 6:**
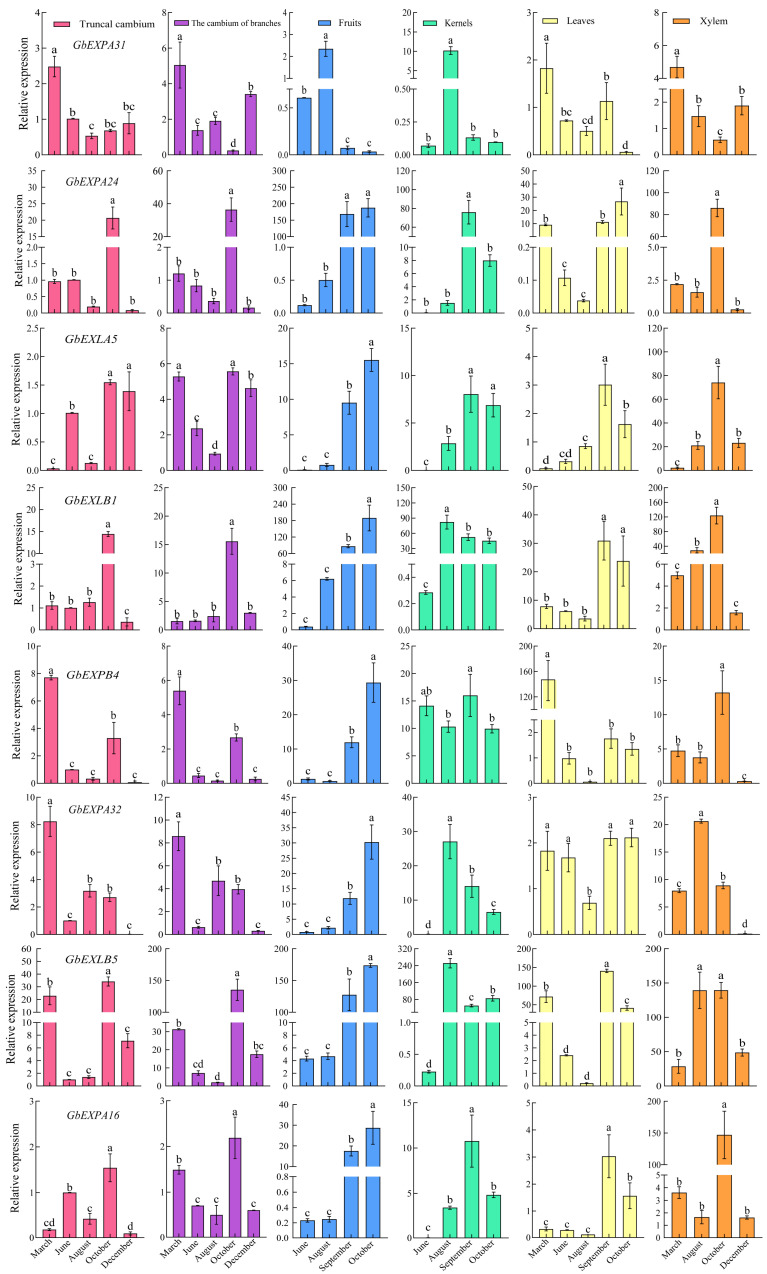
Relative expressions of *GbEXPs* in Ginkgo developing tissues. Lowercase letters indicate significant differences at *p* < 0.05 according to Duncan’s test (values are the means ± SDs of three independent biological replicates).

To understand the potential roles of *GbEXPs* in the developmental process of Ginkgo tissues, the transcript and relative expression profiles were investigated. Most *GbEXPs* showed preferential expression in the developing and rapidly expanding tissues, such as staminate strobili, cambium, young stems, immature leaves, kernels, and fruits, while another specific expression appeared in mature or senescent tissues, such as roots, mature kernels and fruits, and yellow leaves (Figure 5D). A majority of *GbEXPs* were not constitutively expressed in Ginkgo tissues (Figure 5D), showing a high correlation between specific expression and the degree of tissue development. Thus, eight *GbEXPs* were selected for further qRT-PCR assays to analyze their functions in tissue development, and more especially for vascular cambium. *GbEXPA31* was preferentially expressed in kernels in August (Figure 6), and this was consistent with the transcriptome profile and the regulatory element prediction. Similar to *GbEXPA31*, *GbEXPA32* was mainly expressed in kernels (August) and fruits (October), suggesting that *GbEXPA31* and *GbEXPA32* may play an important role in developing kernels. Moreover, *GbEXPA32* may also function as a ripening-regulated expansin, and a situation was found in *GbEXLB1*, as it showed predominant expression in mature fruits (October). *GbEXPA24*, as the most homologous to *GbEXPA31*, had a different expression tendency in the five tissues (cambium, fruits, kernels, leaves, and xylem); its highest level was observed in fruits (October). *GbEXLB5* was highly expressed in developing cambium, fruits, kernels, leaves, and xylem, and the relative expression levels were greater than 100 in certain developmental stages for each tissue (Figure 6). *GbEXPB4* was mainly expressed in leaves (March). Overall, excluding *GbEXPA31*, *GbEXPB4*, and *GbEXLB5*, the remaining five *GbEXPs* all tended to be preferentially expressed in mature fruits (October), followed by xylem (October), which may be the functional indication of these five genes in Ginkgo fruits’ development. In summary, the transcripts and qRT-PCR analysis suggested that *GbEXPs* had highly tissue-specific expression characteristics (Figure 6).

### 2.7. Transcriptional Profiles under Phytohormones Exposure and Abiotic Stress

*EXPs* were previously verified to be involved in response of exogenous plant hormones and abiotic stress [15,37]. Thus, the transcript abundances related to plant hormones and stress responsiveness were investigated. A total of 14, 14, 27, and 2 *GbEXPs* exhibited different expression levels to varying degrees under exposures to MeJA, SA, UV-B, and drought stress, respectively (Supplementary Figure S7). *GbEXPA24*, *GbEXLA4*, and *GbEXPA8* were downregulated after MeJA stimulation (Supplementary Figure S7A).

Different from what was observed for MeJA, *GbEXPA21* and *GbEXLA4* were induced under the SA treatment. *GbEXPA5-8* and *GbEXPA10-11* mapped to chr1 exhibited the consistent expression patterns under UV-B exposure, and their expression levels were downregulated, with *GbEXPA5*-*8* being the most prominent (Supplementary Figure S7B). These findings verified the segmental duplication events among these four *GbEXPAs* and *GbEXPA13*, implying their conservative function in dealing with UV-B stimulation; meanwhile, while *GbEXLA4-5* and *GbEXLB1* were upregulated in this treatment (Supplementary Figure S7B). *GbEXPA10-11*, *GbEXPA6-7,* and *GbEXLA4* were downregulated after 24 h in PEG-6000 stimulation and gradually recovered between 48 and 72 h, with the lowest peak at 24 h and the highest peak at 72 h (Supplementary Figure S7C).

### 2.8. Expression Profiles of GbEXPs under Exogenous Abscisic acid (ABA), Salicylic Acid (SA), and Brassinolide (BR) Treatments

**Figure 7:**
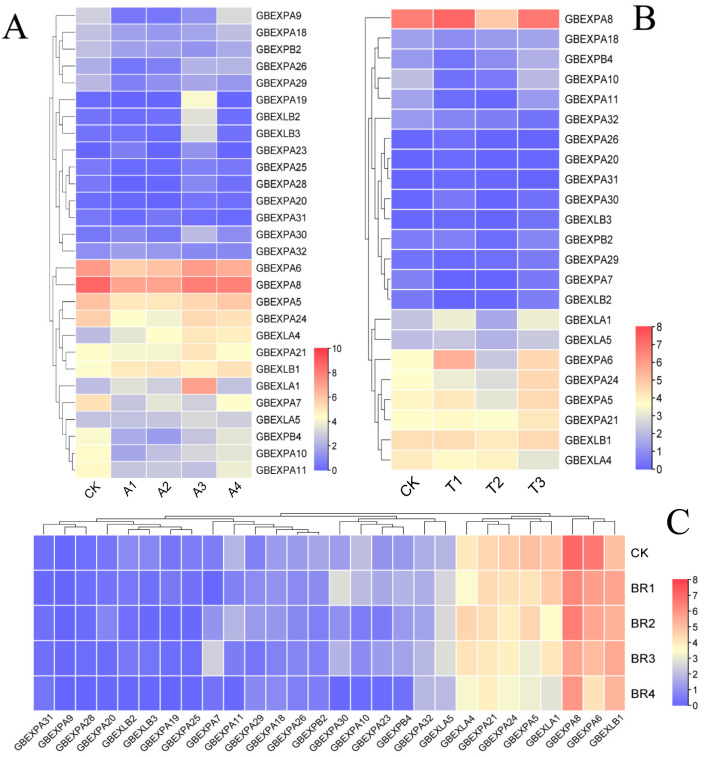
Expression profiles of GbEXPs under exogenous ABA, SA, and BR treatments. Transcript abundance TPM values were transformed into log2(TPM + 1) to construct heatmaps. (**A**) GbEXPs expression under induction of ABA. (**B**) GbEXPs expression under exogenous SA spraying. (**C**) GbEXPs expression under exogenous BR.

The exogenous ABA, SA, and BR were sprayed to evaluate whether *GbEXPs* were induced in response to phytohormones. The transcriptome profiling quantified *GbEXP* expression under these three treatments. Most *GbEXPs* (e.g., *GbEXPA7*, *GbEXPA10-11*, and *GbEXPA24*) were inhibited at 0.5 and 1 mmol/L ABA (A1 and A2), but this inhibition was relieved when the concentration increased to 1.5–2 mmol/L (A3 and A4) (Figure 7A). Under 2 mmol/L SA (T2), some *GbEXPs* were suppressed to the lowest level, including *GbEXLA1*, *GbEXLA5*, *GbEXPA5*, *GbEXPA6*, *GbEXPA21*, and *GbEXPA24*, and as the concentration increased to 3 mmol/L (T3), they were upregulated compared to their CKs (Figure 7B). Moreover, a downregulated expression was also found in the BR treatment, producing results consistent with those under SA, especially at a 2 mg/L (BR4) concentration (Figure 7C).

## 3. Discussion

As one of the most studied structural proteins that promote primary cell-wall growth in plants, *EXPs* can fill microfibrils and bind cellulose networks to form independent reticular systems [8,38], enhancing the strength and toughness of cell walls to maintain continuous cell-wall extension [39]. Here, to better understand the developmental regulation of *EXPs* in Ginkgo, we systematically identified 46 *GbEXP* members. Similar to other plant species [38,39], the *EXPA* subgroup in Ginkgo accounted for the majority of all *GbEXPs* (69.6%); this percentage was evidenced by the syntenic analysis of gene expansion, which included a total of 16 fragmental duplication pairs, with 9 out of 16 pairs undergoing extensive purifying selection and the remaining 7 pairs exhibiting strong positive evolutionary selection [40].

In phylogenesis, *GbEXPA31* was located in a phylogenetic clade with *AtEXPA2*. *AtEXPA2* was reported to be involved in Arabidopsis seed germination [41]. Blastp search showed that *GbEXPA31* had 78.85% sequence identity to the *CLEXPA2* and *ClLEXPA1* within all *GbEXPs*, and *CLEXPA1* and *CLEXPA1-2* were involved in cambium development in *Cunninghamia lanceolata* [42]. To further investigate the expansive growth function of *GbEXPA31*, we extracted *GbEXPA31* from the Ginkgo vascular cambium and analyzed its structural features and subcellular localization. *GbEXPA31* was located in the nucleus, cell wall, plasma membrane, and chloroplast. Moreover, it was preferentially expressed in Ginkgo kernels during August, and a previous prediction suggested that it contained a cis-element related to seed development; thus, the analysis for *GbEXPA31* might verify our putative identification for the *GbEXP* family. We also found that the light-response regulatory element (G-box/ACE/GT1-motif) was the most abundant in *GbEXPs*, and all members contained at least one, suggesting that light-induced responses were essential for the expansive function of these expansins. The GO annotation showed that 32 *GbEXPAs* were involved in plant cell-wall organization, and of these, *GbEXPA26* was only located in the extracellular region, while other *GbEXPAs* were located in the membrane and extracellular region; *GbEXPA14* was also present in the cell wall. These common annotation terms in *GbEXPAs* may indicate their functional redundancy [33].

Gene-expression patterns are closely involved in their biological functions [31]. Expansin in different subgroups had different expansive functions [15]; most *EXPAs* tended to be constitutively expressed [43,44], while some (*EXPBs*, *EXLAs*, and *EXLBs*) were not only drastically affected by tissue development and environment but also had obvious temporal- and spatial-specific expression preferences [44,45]. Thus, RT-qPCR and transcriptome profiling in Ginkgo tissues (leaves, fruits, kernels, cambium, and xylem) were analyzed to investigate these effects, and the results showed that most *GbEXPs* were highly expressed in rapidly developing tissues, such as immature kernels, fruit, and cambium. Moreover, *GbEXPA32*, *GbEXPA24*, and *GbEXLB1* had high expression in fruit (October), suggesting their roles in fruit maturation. Under the PEG-6000 stimulation, most *GbEXPs* showed downregulation after 24 h and 48 h from treatment and then recovered at 72 h. It is interesting that *GbEXLA4* located in the same subgroup with *AtEXLA2* was substantially upregulated; overexpression of *AtEXLA2* might reduce the hypocotyls’ cell-wall strength [45]. Thus, we can infer that drought induced cell-wall expansion within a short time, improving the water-use efficiency and reducing the internal water potential in cells to absorb extra water and mitigate the damage under stress [46,47]. *EXPs* are also regulated by exogenous plant hormones, such as BR and ABA [48,49]. In our work, several *GbEXPAs*, *GbEXLA1,* and *GbEXLA4* fluctuated between up- and downregulation under mild or severe ABA and SA induction, especially for *GbEXLA1* and *GbEXLA4*, suggesting that different phytohormones may have specificity in these gene regulations, as is consistent with the complex mechanism of phytohormones stimulating plant growth and development [50]. Some *EXPs* only performed their function of loosening and expanding the cell wall in specific cell-growth states, and these *EXPs* were indirectly regulated by specific phytohormone-induced signaling cascades [48,49,50]. However, the mechanisms in the differential expression of *GbEXPs* induced by exogenous plant hormones need to be further investigated by means of molecular biology.

## 4. Materials and Methods

### 4.1. Identification and Phylogenetic Analysis of the GbEXP Family

The Ginkgo genome was obtained from Liu et al. [51]. A total of 35 and 36 expansins in *Arabidopsis thaliana* and *Populus trichocarpa*, respectively, were downloaded from EXPANSIN CENTRAL (http://www.personal.psu.edu/fsl/ExpCentral/, accessed on 10 September 2021) as query sequences to blast against the Ginkgo expansin proteins by local blast (https://ftp.ncbi.nlm.nih.gov/blast/executables/blast+/LATEST/, accessed on 15 September 2021) (E-value < 1 × 10^−5^). The candidate members were submitted to the Conserved Domain Database (https://www.ncbi.nlm.nih.gov/cdd, accessed on 20 September 2021) and Pfam (http://pfam.xfam.org/, accessed on 22 September 2021) for verification of the conserved domains DPBB_1 (pfam03330) and Pollen_allerg_1 (pfam01357), and the putative members without two specific domains or complete open reading frames were removed. Expansin protein sequences from *Arabidopsis*, Populus, and Ginkgo were utilized to analyze the phylogenetic relationship, using MEGA 7.0 software [21], with the maximum likelihood method by 1000 replications of bootstrap analysis.

### 4.2. Gene Cloning, Characterization, and Subcellular Localization of GbEXPA31

The CDS of *GbEXPA31* was amplified and cloned into the 5 min TM TA/Blunt-Zero Cloning Kit to verify the putative nucleic acid sequence. The orthologs of *GbEXPA31* were searched using Blastp (http://www.ncbi.nlm.nih.gov/BLAST/, accessed on 10 August 2021). Those expansin protein sequences with more than 80% identity were used for the multiple sequence alignment by DNAMAN 6.0.3 (https://www.lynnon.com/downloads.html, accessed on 10 August 2021). The secondary and tertiary structures were predicted by the GOR IV method [52] and SWISS-MODEL (http://swissmodel.expasy.org/, accessed on 1 November 2021), respectively. The hydrophobicity and hydrophilicity were determined by Protscale (http://web.expasy.org/protsc-ale/, accessed on 23 September 2021).

*GbEXPA31* lacking stop codon was amplified using specific primers designed with SnapGene software (https://www.snapgene.com/, accessed on 8 November 2021) according to the CAM-EGFP plasmid. The blank vector CAM-EGFP was digested with Xho I and EcoR, and *GbEXPA31* was inserted into CAM-EGFP to fuse the EGFP under the driven of CaMV 35S promotor, using homologous recombinase NovoRec^®^ (Novoprotein Scientific Inc., Shanghai, China). The ligated system was successively transferred into DH5α and GV3101 competent cells, LB liquid medium was added into competent cells, and the bacterial solution was smeared on LB solid medium containing kanamycin. The sequencing results for the positive clones are shown in Supplementary Figures S1 and S2 The suspension containing *GbEXPA31* was gently injected into the back of tobacco leaves. GFP fluorescence distributions were observed under a laser scanning confocal microscope (FV3000, Olympus, Tokyo, Japan); the wavelength of GFP in GFP fluorescence channel and autofluorescence in chloroplast channel, respectively, was 488 nm and 561 nm [53].

### 4.3. Physicochemical Property Determination, Motif, and Cis-Element Analysis

Protein characteristics, molecular weight, and theoretical pI were determined using the ProtParam tool (https://web.expasy.org/protparam/, accessed on 23 September 2021). Subcellular localizations were predicted by WoLF PSORT (http://psort.hgc.jp/, accessed on 6 October 2021). Physical chromosomal locations were identified using Gene Location Visualize of TBtools [54]. Conserved protein motifs were searched by MEME (https://meme-suite.org/meme/tools/meme, accessed on 8 October 2021) and specifying the motifs number as ten. The 2 kp upstream sequences of *GbEXPs* start codon were extracted and submitted to PlantCARE (http://bioinformatics.psb.ugent.be/webtools/plantcare/html/, accessed on 11 October 2021) to predict the cis-acting elements. The conserved motifs and acting elements were displayed with TBtools [54].

### 4.4. GBEXPs Collinear Analysis

The two-sequence files of TBtools were used to search the paralogous sequences within *GbEXPs* with an E-value of 1 × 10^−5^. The paralogous file was employed to predict the collinear relationship by the Quick MCScanX Wrapper. The nonsynonymous and synonymous substitution rate (Ka/Ks) values in the duplicated pairs were calculated using DnaSP6 software (http://www.ub.edu/dnasp/, accessed on 15 October 2021).

### 4.5. Transcriptome Profiling Analysis and GO Annotation

Fastq files of RNA-sequencing (RNA-seq) in different tissues, including buds, staminate, and ovulate strobili (PRJNA289172); root (PRJNA373812); stem (PRJNA473396); cambium (PRJNA488475); developing leaves (PRJNA473396, PRJNA517218, and PRJNA578374); fruits (PRJNA473396); and developing kernels (PRJNA292849), were searched with the accession numbers and downloaded from the EMBL-EBI (https://www.ebi.ac.uk/ena/browser/home, accessed on 18 October 2021) database, as well as the data under treatments of exogenous methyl jasmonate (MeJA) (PRJNA553587), salicylic acid (SA) (PRJNA598887), UV-B (PRJNA595103), and drought exposure (PRJNA604486) from a published study [55]. Raw data were filtered using Trimmomatic [56], and then FastQC evaluation was conducted for high-quality data [57], clean reads were mapped to genome with STAR [58], the transcript abundance was calculated as transcripts per million (TPM) value with RSEM [59], and TPM was converted into log2(TPM + 1) values with Micosoft excel 2010 (accessed on 20 April 2020) to draw heatmaps by TBtools [54]. GbEXP sequences were submitted to Protein ANNotation with Z-scoRE server [60] to predict the GO functions that could be annotated.

### 4.6. GbEXP Expression Validation in Ginkgo Tissues by qRT-PCR

We harvested the developing tissues from nine ≈20-year-old female Ginkgo trees, including leaves, fruits, kernels, xylem, vascular cambium from trunk at breast height, and 2–3-year-old branches of the middle canopy. Each tissue was collected from three independent trees as one biological replicate during August 2021 and June 2022. Samples were placed in liquid nitrogen and transferred to −80 °C for RNA extraction. Total RNA was extracted with E.Z.N.A.^®^ Plant RNA Kit (Omega, Nanjing, China), according to the instruction; high-quality RNA from the samples was used for cDNA synthesis using MonScript™ RTIII All-in-One Mix with dsDNase (Monad, Nanjing, China). Eight *GbEXPs* in four subgroups, including *GbEXPA31*, were quantified by qRT-PCR on an Applied Biosystems^®^ 7500 Real Time PCR System as the instruction of MonAmp™ SYBR^®^ Green qPCR Mix (Monad, Nanjing, China) with specific primers (Supplementary Table S2); the reaction program in PCR was fully followed as the introduction of kit. Ginkgo *GAPDH* was utilized as a housekeeping gene to determine the relative expression with the 2^−ΔΔCT^method [61].

### 4.7. Phytohormone ABA, BR and SA Application

Exogenous abscisic acid (ABA) and brassinolide (BR) were sprayed on the leaf surface of one-year-old potted Ginkgo seedlings, and 0 mg. L-1 (CK), 0.5 mg. L-1 (A-1/BR1), 1 mg. L-1 (A-2/BR2), 1.5 mg. L-1 (A-3/BR3), and 2 mg. L-1 (A-4/BR4) were tested separately for ABA or BR spraying. The control was distilled water containing 0.02% Tween 20, with 30 seedlings, which were sprayed in each ABA or BR concentration, yielding three biological replicates. After the first spray was initiated on 31 July 2020, 5 mL of ABA or BR solution at the designated concentration was sprayed on each seedling every 5 d, and mature leaves under the four treatments for the two phytohormones were harvested for growth response and gene-expression determination on day 20 after completing the five sprays. The sequencing data used in this assay were submitted to CNCB (accession no. PRJCA010326). The RNA-seq data of spraying exogenous salicylic acid (SA) were obtained from our previous study [55].

## 5. Conclusions

In this study, we systematically identified and characterized 46 *GbEXPs* in terms of phylogeny, motif distribution, cis-regulatory elements, chromosomal localization, GO annotation, and gene duplication for the first time. *GbEXPA31* was further selected for full-length CDS cloning and subcellular localization to verify our identification, and the result showed that *GbEXPA31* might play a role in Ginkgo’s kernel development. Most *GbEXPs* contained abundant light-response and MeJA-response cis-elements. The tissue-preferential expression of transcriptome and qRT-PCR analyses indicated that most *GbEXPs* had specific expressions in Ginkgo kernels, fruits, or xylem. Under the abiotic stresses and exogenous ABA, SA, and BR treatments, *GbEXLA4*, *GbEXLA5*, and *GbEXPA5*, *GbEXPA6*, *GbEXPA8*, and *GbEXPA24* were downregulated by specific induction, proving the suppressive regulation by stimulations of UV-B, drought stress, and exogenous plant hormones. Collectively, this study provided comprehensive information for *GbEXPs* and an insight into the expression pattern of *GbEXPs* tissue-specific and environmental response.

## Figures and Tables

**Table 1:** Ka (nonsynonymous), Ks (synonymous), and Ka/Ks ratios of duplicated *GbEXPs* in Ginkgo.

Gene 1	Gene 2	Ka	Ks	Ka/Ks (ω)	Selection	Duplication Mode
*GbEXPA13*	*GbEXPA3*	0.39446	0.16952	2.32692	Positive	Segmental
*GbEXPA13*	*GbEXPA4*	0.22435	1.40855	0.15928	Purifying	Segmental
*GbEXPA13*	*GbEXPA5*	0.13250	0.99140	0.13365	Purifying	Segmental
*GbEXPA13*	*GbEXPA6*	0.16868	1.45943	0.11558	Purifying	Segmental
*GbEXPA13*	*GbEXPA7*	0.39487	0.16683	2.36690	Positive	Segmental
*GbEXPA13*	*GbEXPA8*	0.17167	1.32996	0.12908	Purifying	Segmental
*GbEXPA13*	*GbEXPA9*	0.15953	1.57192	0.10149	Purifying	Segmental
*GbEXPA13*	*GbEXPA10*	0.39076	0.27286	1.43209	Positive	Segmental
*GbEXPA13*	*GbEXPA11*	0.14134	1.55588	0.09084	Purifying	Segmental
*GbEXPA13*	*GbEXPA20*	0.23166	2.44114	0.09490	Purifying	Segmental
*GbEXPA13*	*GbEXPA21*	0.54596	0.37531	1.45469	Positive	Segmental
*GbEXPA19*	*GbEXPA20*	0.57707	0.45020	1.28181	Positive	Segmental
*GbEXPA19*	*GbEXPA21*	0.42454	1.47024	0.28876	Purifying	Segmental
*GbEXPA14*	*GbEXPA18*	0.14676	1.55673	0.09427	Purifying	Segmental
*GbEXPA18*	*GbEXPA19*	0.58062	0.51528	1.12680	Positive	Segmental
*GbEXPA14*	*GbEXPA19*	0.58034	0.39994	1.45107	Positive	Segmental

## Data Availability

The data and materials supporting the conclusions of this study are included within the article.

## Supplementary Materials

The supporting information can be downloaded at: https://www.mdpi.com/article/10.3390/ijms24065901/s1.
