# Supplementary material for: Genome-Wide Identification of Expansin Gene Family and Their Response under Hormone Exposure in Ginkgo biloba L
Source: Int J Mol Sci. 2023 Mar 21;24(6):5901. doi: 10.3390/ijms24065901 (PMC10053239; doi:10.3390/ijms24065901)
Supplement: Supplementary file 1 [file ijms-24-05901-s001.zip › Figure S1-S7.pdf]

A B C

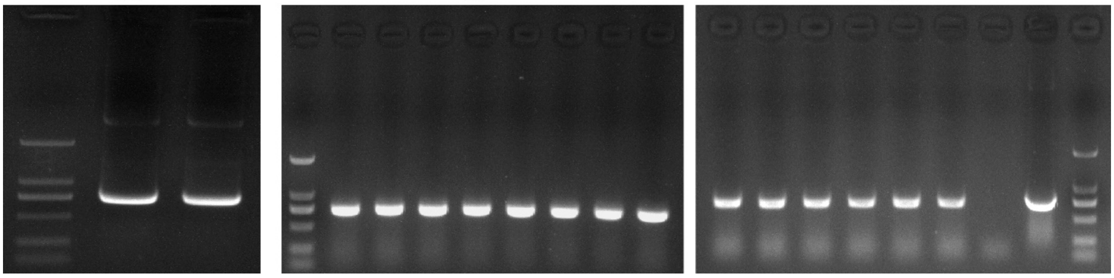

Figure.S1. Identification the positive cloning in vector construction by gel electrophoresis in subcellular localization of *GbEXPA31*. A. CDS full-length amplification of *GbEXPA31* B. Identification the positive cloning of *GbEXPA31* in transformed Escherichia coli. C. Identification the positive cloning of *GbEXPA31* in transformed Agrobacterium tumefaciens.

```
GbEXPA31+Homologous_splice_sequence   ..........................TTGGAGAGGACACG    14
GbEXPA31_cloning_1                    CCTCTATATAAGGAAGTTCATTTCATTTGGAGAGGACACG    40
GbEXPA31_cloning_2                    CCTCTATATAAGGAAGTTCATTTCATTTGGAGAGGACACG    40
GbEXPA31_cloning_3                    CCTCTATATAAGGAAGTTCATTTCATTTGGAGAGGACACG    40
Consensus                                                       ttggagaggacacg

GbEXPA31+Homologous_splice_sequence   CTCGAGATGGCCCAGTTTCTCCAGACATGGGGCTGGACGA    54
GbEXPA31_cloning_1                    CTCGAGATGGCCCAGTTTCTCCAGACATGGGGCTGGACGA    80
GbEXPA31_cloning_2                    CTCGAGATGGCCCAGTTTCTCCAGACATGGGGCTGGACGA    80
GbEXPA31_cloning_3                    CTCGAGATGGCCCAGTTTCTCCAGACATGGGGCTGGACGA    80
Consensus                             ctcgagatggcccagtttctccagacatggggctggacga

GbEXPA31+Homologous_splice_sequence   ATGCTCATGCAACTTTCTATGGAGGCAGCGATGCCACTGG    94
GbEXPA31_cloning_1                    ATGCTCATGCAACTTTCTATGGAGGCAGCGATGCCACTGG   120
GbEXPA31_cloning_2                    ATGCTCATGCAACTTTCTATGGAGGCAGCGATGCCACTGG   120
GbEXPA31_cloning_3                    ATGCTCATGCAACTTTCTATGGAGGCAGCGATGCCACTGG   120
Consensus                             atgctcatgcaactttctatggaggcagcgatgccactgg

GbEXPA31+Homologous_splice_sequence   TACAATGGGCGGCGCGTGTGGGTATGGAAATATGTACAGC   134
GbEXPA31_cloning_1                    TACAATGGGCGGCGCGTGTGGGTATGGAAATATGTACAGC   160
GbEXPA31_cloning_2                    TACAATGGGCGGCGCGTGTGGGTATGGAAATATGTACAGC   160
GbEXPA31_cloning_3                    TACAATGGGCGGCGCGTGTGGGTATGGAAATATGTACAGC   160
Consensus                             tacaatgggcggcgcgtgtgggtatggaaatatgtacagc

GbEXPA31+Homologous_splice_sequence   CAAGGATATGGCACGAACACGGCAGCTCTGAGCACTGCTC   174
GbEXPA31_cloning_1                    CAAGGATATGGCACGAACACGGCAGCTCTGAGCACTGCTC   200
GbEXPA31_cloning_2                    CAAGGATATGGCACGAACACGGCAGCTCTGAGCACTGCTC   200
GbEXPA31_cloning_3                    CAAGGATATGGCACGAACACGGCAGCTCTGAGCACTGCTC   200
Consensus                             caaggatatggcacgaacacggcagctctgagcactgctc

GbEXPA31+Homologous_splice_sequence   TGTTCAACAATGGGCTGAGCTGTGGAGCATGCTATGAAAT   214
GbEXPA31_cloning_1                    TGTTCAACAATGGGCTGAGCTGTGGAGCATGCTATGAAAT   240
GbEXPA31_cloning_2                    TGTTCAACAATGGGCTGAGCTGTGGAGCATGCTATGAAAT   240
GbEXPA31_cloning_3                    TGTTCAACAATGGGCTGAGCTGTGGAGCATGCTATGAAAT   240
Consensus                             tgttcaacaatgggctgagctgtggagcatgctatgaaat

GbEXPA31+Homologous_splice_sequence   CAAATGTAACGACGACCCACAGTGGTGTCTGTCCAACACA   254
GbEXPA31_cloning_1                    CAAATGTAACGACGACCCACAGTGGTGTCTGTCCAACACA   280
GbEXPA31_cloning_2                    CAAATGTAACGACGACCCACAGTGGTGTCTGTCCAACACA   280
GbEXPA31_cloning_3                    CAAATGTAACGACGACCCACAGTGGTGTCTGTCCAACACA   280
Consensus                             caaatgtaacgacgacccacagtggtgtctgtccaacaca

GbEXPA31+Homologous_splice_sequence   ATAGTTGTGACTGCAACGAACTTCTGCCCGCCCAATACTG   294
GbEXPA31_cloning_1                    ATAGTTGTGACTGCAACGAACTTCTGCCCGCCCAATACTG   320
GbEXPA31_cloning_2                    ATAGTTGTGACTGCAACGAACTTCTGCCCGCCCAATACTG   320
GbEXPA31_cloning_3                    ATAGTTGTGACTGCAACGAACTTCTGCCCGCCCAATACTG   320
Consensus                             atagttgtgactgcaacgaacttctgcccgcccaatactg

GbEXPA31+Homologous_splice_sequence   CTCTTCCCAACAATGCCGGCGGATGGTGCAATCCTCCTCT   334
GbEXPA31_cloning_1                    CTCTTCCCAACAATGCCGGCGGATGGTGCAATCCTCCTCT   360
GbEXPA31_cloning_2                    CTCTTCCCAACAATGCCGGCGGATGGTGCAATCCTCCTCT   360
GbEXPA31_cloning_3                    CTCTTCCCAACAATGCCGGCGGATGGTGCAATCCTCCTCT   360
Consensus                             ctcttcccaacaatgccggcggatggtgcaatcctcctct

GbEXPA31+Homologous_splice_sequence   CCTGCACTTTGATCTCTCAGAGCCTGTTTTTCAGCATATT   374
GbEXPA31_cloning_1                    CCTGCACTTTGATCTCTCAGAGCCTGTTTTTCAGCATATT   400
GbEXPA31_cloning_2                    CCTGCACTTTGATCTCTCAGAGCCTGTTTTTCAGCATATT   400
GbEXPA31_cloning_3                    CCTGCACTTTGATCTCTCAGAGCCTGTTTTTCAGCATATT   400
Consensus                             cctgcactttgatctctcagagcctgtttttcagcatatt

GbEXPA31+Homologous_splice_sequence   GCCAAGTTCAGGGCAGGCATTGTGCCTGTAATGTACCAAA   414
GbEXPA31_cloning_1                    GCCAAGTTCAGGGCAGGCATTGTGCCTGTAATGTACCAAA   440
GbEXPA31_cloning_2                    GCCAAGTTCAGGGCAGGCATTGTGCCTGTAATGTACCAAA   440
GbEXPA31_cloning_3                    GCCAAGTTCAGGGCAGGCATTGTGCCTGTAATGTACCAAA   440
Consensus                             gccaagttcagggcaggcattgtgcctgtaatgtaccaaa

GbEXPA31+Homologous_splice_sequence   GGGTACCCTGCCAGAAGAAGGGGGGGATTCGGTTCACAAT   454
GbEXPA31_cloning_1                    GGGTACCCTGCCAGAAGAAGGGGGGGATTCGGTTCACAAT   480
GbEXPA31_cloning_2                    GGGTACCCTGCCAGAAGAAGGGGGGGATTCGGTTCACAAT   480
GbEXPA31_cloning_3                    GGGTACCCTGCCAGAAGAAGGGGGGGATTCGGTTCACAAT   480
Consensus                             gggtaccctgccagaagaagggggggattcggttcacaat

GbEXPA31+Homologous_splice_sequence   CAATGGGCATTCATACTTCAACCTCGTTCTGCTAACCAAC   494
GbEXPA31_cloning_1                    CAATGGGCATTCATACTTCAACCTCGTTCTGCTAACCAAC   520
GbEXPA31_cloning_2                    CAATGGGCATTCATACTTCAACCTCGTTCTGCTAACCAAC   520
GbEXPA31_cloning_3                    CAATGGGCATTCATACTTCAACCTCGTTCTGCTAACCAAC   520
Consensus                             caatgggcattcatacttcaacctcgttctgctaaccaac

GbEXPA31+Homologous_splice_sequence   GTAGGAGGTGCAGGGGATGTCAATGCGGTTTCCATCAAGG   534
GbEXPA31_cloning_1                    GTAGGAGGTGCAGGGGATGTCAATGCGGTTTCCATCAAGG   560
GbEXPA31_cloning_2                    GTAGGAGGTGCAGGGGATGTCAATGCGGTTTCCATCAAGG   560
GbEXPA31_cloning_3                    GTAGGAGGTGCAGGGGATGTCAATGCGGTTTCCATCAAGG   560
Consensus                             gtaggaggtgcaggggatgtcaatgcggtttccatcaagg

GbEXPA31+Homologous_splice_sequence   GCTCAGGAACCGGGTGGCAGCCAATGTCTCGCAACTGGGG   574
GbEXPA31_cloning_1                    GCTCAGGAACCGGGTGGCAGCCAATGTCTCGCAACTGGGG   600
GbEXPA31_cloning_2                    GCTCAGGAACCGGGTGGCAGCCAATGTCTCGCAACTGGGG   600
GbEXPA31_cloning_3                    GCTCAGGAACCGGGTGGCAGCCAATGTCTCGCAACTGGGG   600
Consensus                             gctcaggaaccgggtggcagccaatgtctcgcaactgggg

GbEXPA31+Homologous_splice_sequence   CCAAAATTGGCAGAGCAACTCCAACCTCAACGGACAAAGT   614
GbEXPA31_cloning_1                    CCAAAATTGGCAGAGCAACTCCAACCTCAACGGACAAAGT   640
GbEXPA31_cloning_2                    CCAAAATTGGCAGAGCAACTCCAACCTCAACGGACAAAGT   640
GbEXPA31_cloning_3                    CCAAAATTGGCAGAGCAACTCCAACCTCAACGGACAAAGT   640
Consensus                             ccaaaattggcagagcaactccaacctcaacggacaaagt

GbEXPA31+Homologous_splice_sequence   CTCTCCTTCAAGGTCACCACCAGCGATGGACAGACCGTCA   654
GbEXPA31_cloning_1                    CTCTCCTTCAAGGTCACCACCAGCGATGGACAGACCGTCA   680
GbEXPA31_cloning_2                    CTCTCCTTCAAGGTCACCACCAGCGATGGACAGACCGTCA   680
GbEXPA31_cloning_3                    CTCTCCTTCAAGGTCACCACCAGCGATGGACAGACCGTCA   680
Consensus                             ctctccttcaaggtcaccaccagcgatggacagaccgtca

GbEXPA31+Homologous_splice_sequence   CTTCATACAATGCTGCTCCCAGCAACTGGCAATTTGGCCA   694
GbEXPA31_cloning_1                    CTTCATACAATGCTGCTCCCAGCAACTGGCAATTTGGCCA   720
GbEXPA31_cloning_2                    CTTCATACAATGCTGCTCCCAGCAACTGGCAATTTGGCCA   720
GbEXPA31_cloning_3                    CTTCATACAATGCTGCTCCCAGCAACTGGCAATTTGGCCA   720
Consensus                             cttcatacaatgctgctcccagcaactggcaatttggcca

GbEXPA31+Homologous_splice_sequence   GACTTTTAGCGGAGCCCAACTTGGCAATGAATTCATGGTG   734
GbEXPA31_cloning_1                    GACTTTTAGCGGAGCCCAACTTGGCAATGAATTCATGGTG   760
GbEXPA31_cloning_2                    GACTTTTAGCGGAGCCCAACTTGGCAATGAATTCATGGTG   760
GbEXPA31_cloning_3                    GACTTTTAGCGGAGCCCAACTTGGCAATGAATTCATGGTG   760
Consensus                             gacttttagcggagcccaacttggcaatgaattcatggtg

GbEXPA31+Homologous_splice_sequence   AGCAAGGG.........................                  742
GbEXPA31_cloning_1                    AGCAAGGGCGAGGAGCTGTTCACCGGGGTGGTG                  793
GbEXPA31_cloning_2                    AGCAAGGGCGAGGAGCTGTTCACCGGGGTGGTG                  793
GbEXPA31_cloning_3                    AGCAAGGGCGAGGAGCTGTTCACCGGGGTGGTG                  793
Consensus                             agcaaggg
```

Figure.S2. Sequence alignment of *GbEXPA31* fusing pART-CAM-EGFP.

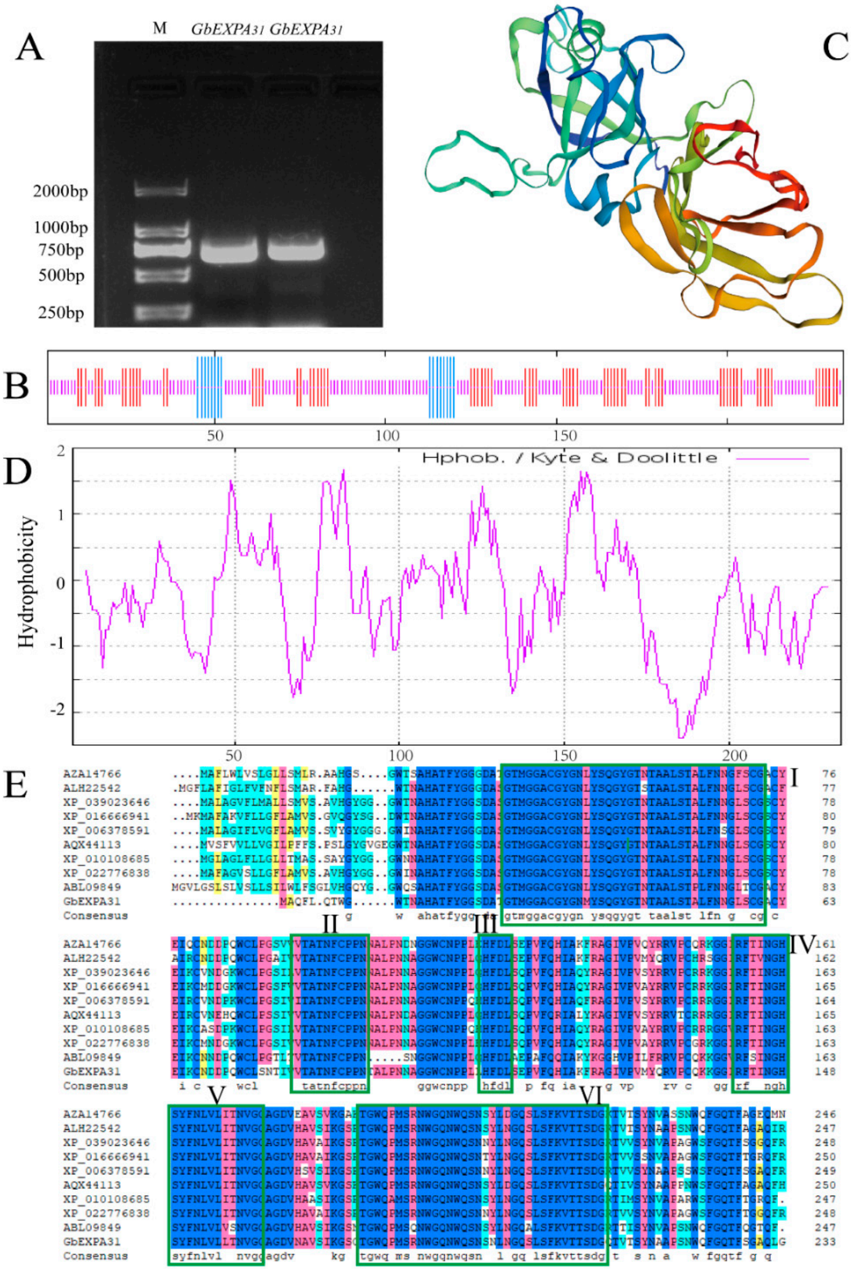

Figure S3. *GbEXPA31* cloning and characteristic analysis of GbEXPA31. (A) Gel electrophoresis image of *GbEXPA31*. (B) Tertiary structure of GbEXPA31 (colors indicate the peptide chain order starting as dark blue from the N-terminus to red at the C-terminus). (C) Secondary structure prediction of GbEXPA31 (long blue, short red, and short purple lines indicate alpha helices, extended strands, and random coils, respectively). (D) GbEXPA31 hydrophobicity characteristic (purple waving line shows the hydrophobicity tendency of all amino acids; the positive and negative values indicate hydrophobicity and hydrophilicity, respectively). (E) Multiple sequence alignment of Ginkgo GbEXPA31 with other species (AZA14766 from *Pinus taeda*; ALH22542 from *Pinus radiata*; XP_039023646 from *Hibiscus syriacus*; XP_016666941 from *Gossypium hirsutum*; XP_006378591 from *Populus trichocarpal*; AQX44113 from *Dendrobium officinale*; XP_010108685 from *Morus notabilis*; XP_022776838 from *Durio zibethinus*; ABL09849 from *Cunninghamia lanceolata*).

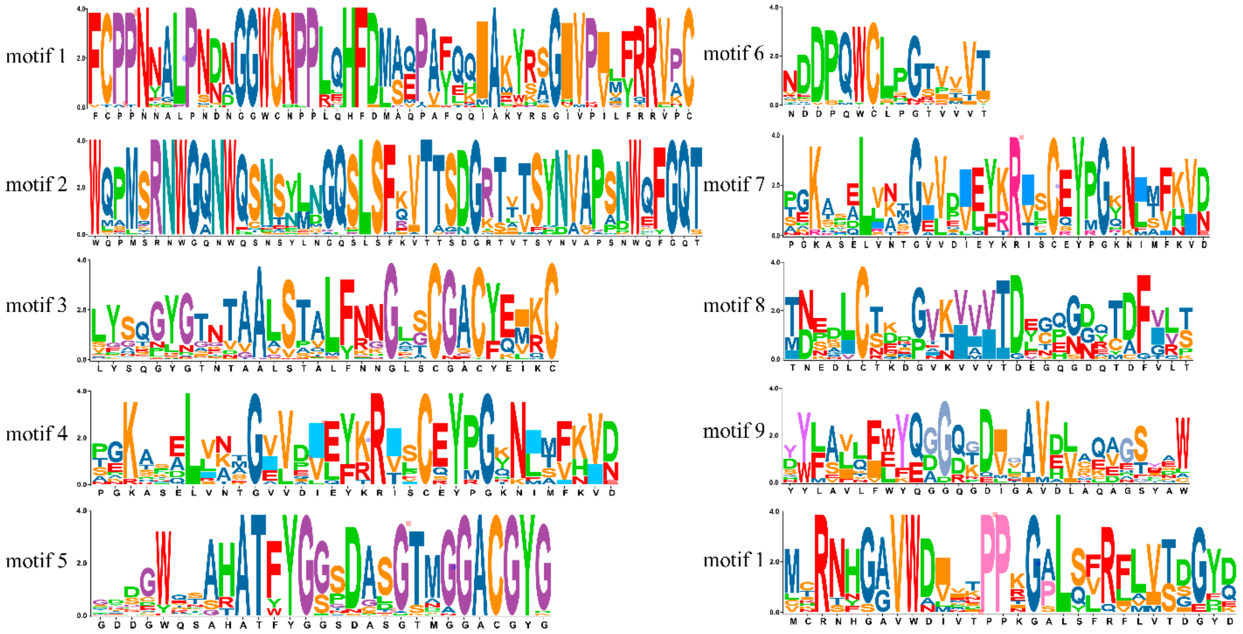

Figure S4. The motif logos in *GbEXP* family.

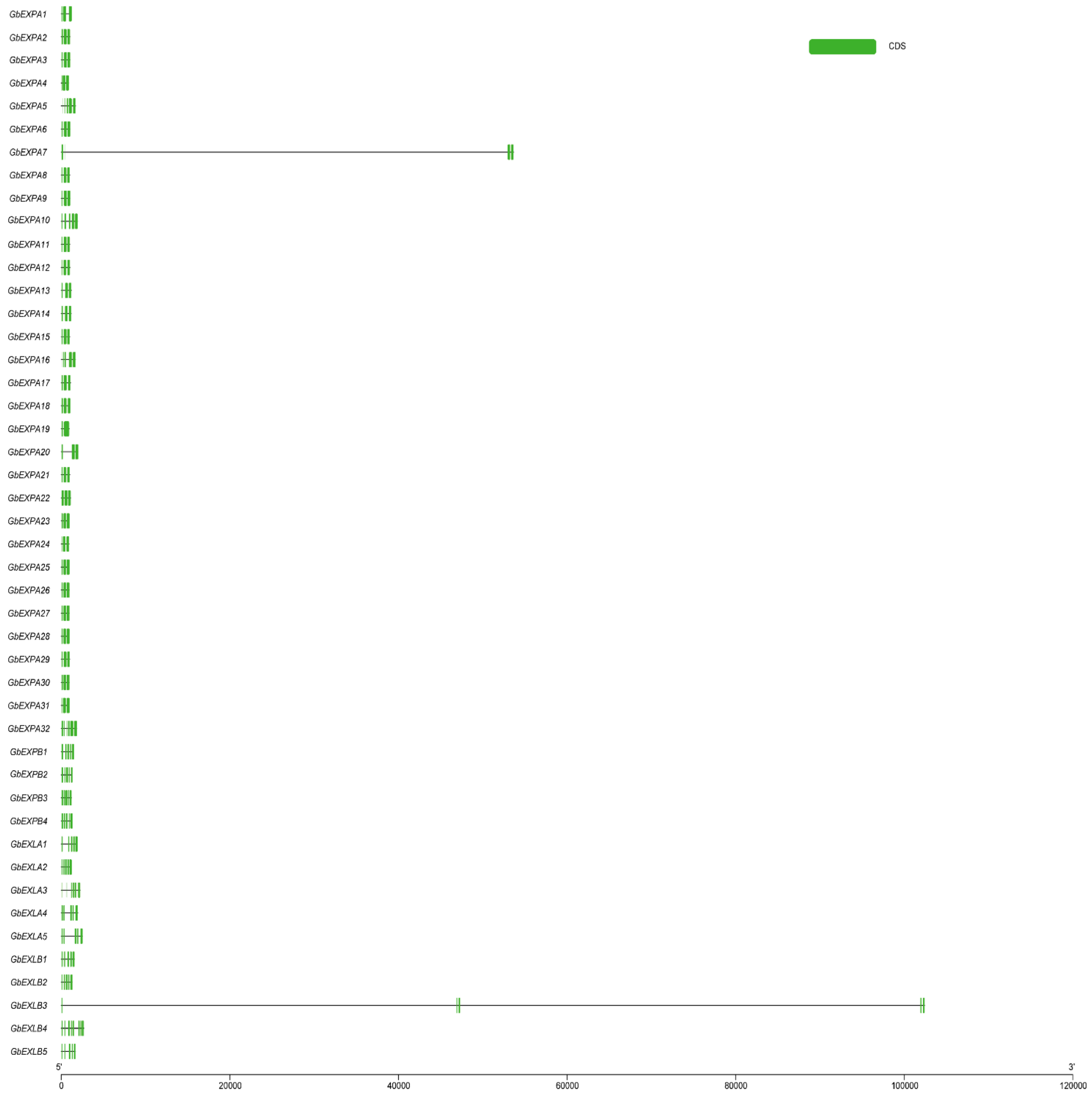

Figure S5. The exon-intron structures of *GbEXP* members.

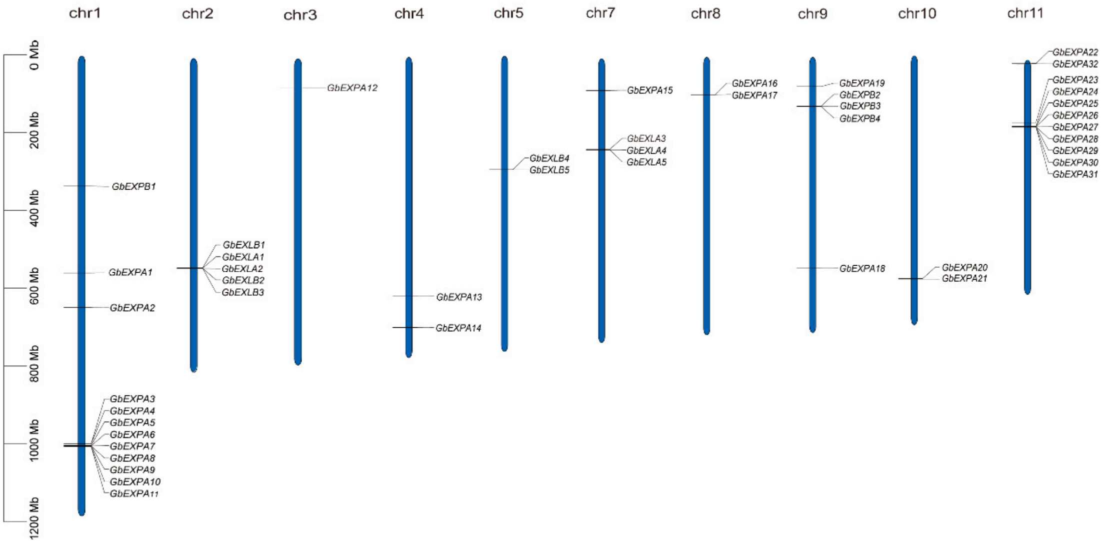

Figure.S6. Physical chromosomal locations of *GbEXP*s

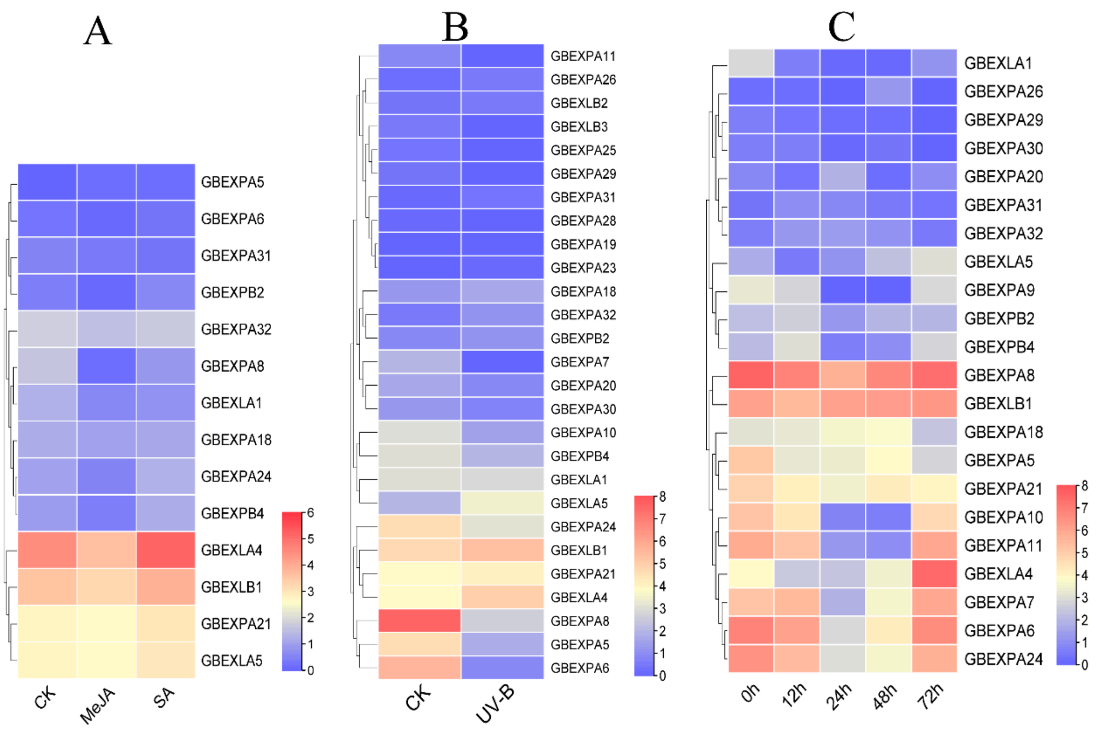

Figure.S7. Heatmap of *GbEXPs* expression in response to abiotic stress. (A) Expression profile of *GbEXPs* under methyl jasmonate (MeJA) and salicylic acid (SA) treatments. (B) Expression profile of *GbEXPs* under UV-B exposure. (C) Expression profile of *GbEXPs* under PEG-6000 stress.
