# Supplementary material for: Genome-Wide Identification of Expansin Gene Family and Their Response under Hormone Exposure in Ginkgo biloba L
Source: Int J Mol Sci. 2023 Mar 21;24(6):5901. doi: 10.3390/ijms24065901 (PMC10053239; doi:10.3390/ijms24065901)
Supplement: Supplementary file 1 [file ijms-24-05901-s001.zip › File S1.Protein sequences in phylogenetic analysis.pdf]

>AtEXP1

MALVTFLFIATLGA

MTSHVNGYAGGGWVNAHATFYGGGDASGTMGGACGYGNLYSQGYGTNTAALSTALFNNGLSCGACFEIRCQNDGKWCLP
GSIVVTATNFCPPNNALPNNAGGWCNPPQQHFDLSQPVFQRIAQYRAGIVPVAYRRVPCVRRGGIRFTINGHSYFNLVL
ITNVGGAGDVHSAMVKGSRTGWQAMSRNWGQNWQSNSYLNGQSLSFKVTTSDGQTIVSNNVANAGWSFGQTFTGAQLR

>AtEXP2

MNLTEYSHILFLSLCTLNFCLYSINS

DDNGGWERGHATFYGGADASGTMGGACGYGNLHSQGYGLQTAALSTALFNSGQKCGACFELQCEDDPEWCIPGSIIVSA
TNFCPPNFALANDNGGWCNPPLKHFDLAEPAFLQIAQYRAGIVPVAFRRVPCEKGGGIRFTINGNPYFDLVLITNVGGA
GDIRAVSLKGSKTDQWQSMSRNWGQNWQSNTYLRGQSLSFQVTDSDGRTVVSYDVVPHDWQFGQTFEGGQF

>AtEXP3

MTATAFRVGLWLAVTASFLLTATNA

KIPGVYSGGPWQNAHATFYGGSDASGTMGGACGYGNLYSQGYGVNTAALSTALFNNGFSCGACFEIKCTDDPRWCVPGN
PSILVTATNFCPPNFAQPSDDGGWCNPPREHFDLAMPMFLKIGLYRAGIVPVSYRRVPCRKIGGIRFTVNGFRYFNLVL
VTNVAGAGDINGVSVKGSKTDWVRMSRNWGQNWQSNAVLIGQSLSFRVTASDRRSSTSWNVAPATWQFGQTFSGKNFRV

>AtEXP4

MAIKLAILFTTFVLFSLADA

RIPGIYSGGAWQNAHATFYGGSDASGTMGGACGYGNLYSQGYGTNTAALSTALFNNGMSCGACFELKCANDPQWCHSGS
PSILITATNFCPPNLAQPSDNGGWCNPPREHFDLAMPVFLKIAQYRAGIVPVSYRRVPCRKRGGIRFTINGHRYFNLVL
ITNVAGAGDIVRASVKGSRTGWMSLSRNWGQNWQSNAVLVGQALSFRVTGSDRRTSTSWNMVPSNWQFGQTFVGKNFRV

>AtEXP5

MGVLVISLLVVHLLAFSVCVQG

GYRRGGHHPGGHMGPWINAHATFYGGGDASGTMGGACGYGNLYSQGYGLETAALSTALFDQGLSCGACFELMCVNDPQW
CIKGRSIVVTATNFCPPGGACDPPNHHFDLSQPIYEKIALYKSGIIPVMYRRVRCKRSGGIRFTINGHSYFNLVLVTNV
GGAGDVHSVSMKGSRTKWQLMSRNWGQNWQSNSYLNGQSLSFVVTTSDRRSVVSFNVAPPTWSFGQTYTGGQFRY

>AtEXP6

MAMLGLVLSVLTTILALSEA

RIPGVYNGGGWETAHATFYGGSDASGTMGGACGYGNLYSQGYGVNTAALSTALFNNGFSCGACFELKCASDPKWCHSGS
PSIFITATNFCPPNFAQPSDNGGWCNPPRPHFDLAMPMFLKIAEYRAGIVPVSFRRVPCRKRGGIRFTINGFRYFNLVL
VTNVAGAGNIVRLGVKGTHTSWMTMSRNWGQNWQSNSVLVGQSLSFRVTSSDRRSSTSWNIAPANWKFGQTFMGKNFRV

>AtEXP7

MGPISSSWSFNKFFSIVFVVFAISGEFVAG

YYRPGPWRYAHATFYGDETGGETMGGACGYGNLFNSGYGLSTAALSTTLFNDGYGCGQCFQITCSKSPHCYSGKSTVVT
ATNLCPPNWYQDSNAGGWCNPPRTHFDMAKPAFMKLAYWRAGIIPVAYRRVPCQRSGGMRFQFQGNSYWLLIFVMNVGG

AGDIKSMAVKGSRTNWISMSHNWGASYQAFSSLYGQSLSFRVTSYTTGETIYAWNVAPANWSGGKTYKSTANFR

>AtEXP8

MYTPSYLKYSIISIISVLFLQGTHG

DDGGWQGGHATFYGGEDASGTMGGACGYGNLYGQGYGTNTAALSTALFNNGLTCGACYEMKCNDDPRWCLGSTITVTAT
NFCPPNPGLSNDNGGWCNPPLQHFDLAEPAFLQIAQYRAGIVPVSFRRVPCMKKGGIRFTINGHSYFNLVLISNVGGAG
DVHAVSIKGSKTQSWQAMSRNWGQNWQSNSYMNDQSLSFQVTTSDGRTLVSNDVAPSNWQFGQTYQGGQF

>AtEXP9

MAAKVITFMAVMVVTAFTANA

KIPGVYTGGPWINAHATFYGEADASGTMGGACGYGNLYSQGYGVNTAALSTALFNNGLSCGSCFELKCINDPGWCLPGN
PSILITATNFCPPNFNQASDNGGWCNPPREHFDLAMPMFLSIAKYKAGIVPVSYRRIPCRKKGGIRFTINGFKYFNLVL
VTNVAGAGDVIKVSVKGSNTQWLDLSRNWGQNWQSNALLVGQSLSFRVKTSDGRSSTSNNIAPSNWQFGQTYSGKNFRV

>AtEXP10

MGHLGFLVMIMVGVMASSVSG

YGGGWINAHATFYGGGDASGTMGGACGYGNLYSQGYGTSTAALSTALFNNGLSCGSCFEIRCENDGKWCLPGSIVVTAT
NFCPPNNALANNNGGWCNPPLEHFDLAQPVFQRIAQYRAGIVPVSYRRVPCRRRGGIRFTINGHSYFNLVLITNVGGAG
DVHSAAIKGSRTVWQAMSRNWGQNWQSNSYLNGQALSFKVTTSDGRTVVSFNAAPAGWSYGQTFAGGQFR

>AtEXP11

MSKSLAGLAVLAALFIAVDAFR

PSGLTNGHATFYGGSDASGTMGGACGYGDLYSAGYGTMTAALSTALFNDGASCGECYRITCDHAADSRWCLKGASVVIT
ATNFCPPNFALPNNNGGWCNPPLKHFDMAQPAWEKIGIYRGGIVPVVFQRVSCYKKGGVRFRINGRDYFELVNIQNVGG
AGSIKSVSIKGSKTGWLAMSRNWGANWQSNAYLDGQALSFSITTTDGATRVFLNVVPSSWSFGQIYSSNVQF

>AtEXP12

MDMKGTYLVTVILLVSTLSVGMC

SNGWIRAHATYYGVNDSPASLGGACGYDNPYHAGFGAHTAALSGELFRSGESCGGCYQVRCDFPADPKWCLRGAAVTVT
ATNFCPTNNNNGWCNLPRHHFDMSSPAFFRIARRGNEGIVPVFYRRVGCKRRGGVRFTMRGQGNFNMVMISNVGGGGSV
RSVAVRGSKGKTWLQMTRNWGANWQSSGDLRGQRLSFKVTLTDSKTQTFLNVVPSSWWFGQTFSSRGRQFV

>AtEXP13

MQRFLLPLLFLALSPPAIC

HYSSSTSSPSSSSVSSDASEWRPARATYYAATNPRDAVGGACGYGDLVKSGYGMATVGLSETLFERGQICGACFELRCV
DDLRWCIPGTSIILTATNFCAPNYGFDPDGGGHCNPPNKHFVLPIEAFEKIAIWKAGNMPVQYRRINCRKEGSMRFTVD
GGGIFISVLITNVAGSGDIAAVKIKGSRTGWLPMGRNWGQNWHINADLRNQAXSFEVTSSDRSXVTSYNVSPKNWNYGQ
TFEGKQFETP

>AtEXP14

MEFFGKMIISLSLMMMIMWKSVDG

YSSGWVNARATFYGGADASGTMGGACGYGNLYSQGYGTNTAALSTALFNGGQSCGACFQIKCVDDPKWCIGGTITVTGT
NFCPPNFAQANNAGGWCNPPQHHFDLAQPIFLRIAQYKAGVVPVQYRRVACRRKGGIRFTINGHSYFNLVLITNVAGAG
DVISVSIKGTNTRWQSMSRNWGQNWQSNAKLDGQALSFKVTTSDGRTVISNNATPRNWSFGQTYTGKQFRAQR

>AtEXP15

MGLLGIALFCFAAMVCSVHG

YDAGWVNAHATFYGGSDASGTMGGACGYGNLYSQGYGTNTAALSTALFNNGLSCGACFEIKCQSDGAWCLPGAIIVTAT
NFCPPNNALPNNAGGWCNPPLHHFDLSQPVFQRIAQYKAGVVPVSYRRVPCMRRGGIRFTINGHSYFNLVLVTNVGGAG
DVHSVAVKGSRTRWQQMSRNWGQNWQSNNLLNGQALSFKVTASDGRTVVSNNIAPASWSFGQTFTGRQFR

>AtEXP16

MAINPLILLTIFPLFLLLSFTDA

GIPRVFSGGSWQTAHATFYGGNDASGTMGGACGYGNLYSQGYGTNTAALSTSLFNSGQSCGACFEIKCVNDPKWCHPGN
PSVFVTATNFCPPNLAQPSDNGGWCNPPRSHFDLAMPVFLKIAEYRAGIVPISYRRVACRKSGGIRFTINGHRYFNLVL
ITNVAGAGDIARTSVKGSKTGWMSLTRNWGQNWQSNAVLVGQSLSFRVTSSDRRTSTSWNIAPSNWQFGQTFVGKNFRV

>AtEXP17

MTKIFSLLVAMIFSTMFFMKISSVSA

GWLQAHATFYGGSDASGTMGGACGYGNLYTDGYKTNTAALSTALFNDGKSCGGCYQILCDATKVPQWCLKGKSITITAT
NFCPPNFAQASDNGGWCNPPRPHFDMAQPAFLTIAKYKAGIVPILYKKVGCRRSGGMRFTINGRNYFELVLISNVAGGG
EISKVWIKGSKSNKWETMSRNWGANYQSNTYLNGQSLSFKVQLSDGSIKAALNVVPSNWRFGQSFKSNVNF

>AtEXP18

MDQNLYRKCLVILSMMAMIGTSMA

TYAGTPWRTASATFYGDDTGSATMGGACGYGNMYDSGYGVATTALSTALFNEGYACGQCFQLKCVSSPNCYYGSPATVV
TATNICPPNYGQASNNGGWCNPPRVHFDLTKPAFMKIANWKAGIIPVSYRRVACKKIGGIRFKFEGNGYWLLVYVMNVG
GPGDIKTMAVKGSRTGWINMSHNWGASYQAFSSLYGQSLSFRLTSYTTRQTIYAYNAAPASWSAGKTYQSKANFN

>AtEXP19

MNRSITFVVLLAVLVSVANLGLA

HVGLTNIDPSWYDAHATFYGDMSGGETMQGACGYGDLFKQGYGLETAALSTALFNNGQTCGACFELMCVSSKWCKPNAG
SIKITATNFCPPNYQEPVQYHWCNPPNKHFDLSMKMFTTVAEYRAGIVPVKFRRVACHKRGGVRFEIKGNPYYIMVLVY
NVGGAGDVSNVEIRGQKSNWIVMKRNWGQIWDTGLDLVGQSLSFIVRTSDGRSMTFFNVAPPNWGFGQTYEAKSNF

>AtEXP20

MDSGLQQLALCLFFILCRLFQATA

EDDWKIATATLSRDRDGSSSVATGGACGYGDLRQSSFAGYSAGLSGKLFNRGSSCGACLEVRCVNHIRWCLQGSPSVVV
TATDFCPPNSGLSSDYGGWCNFPKEHLELSHAAFTGIAETRAEMIPIQYRRVKCGRRGGLRFSLSGSSHFFQVLISNVG
LDGEVVGVKVKGHTTAWIPMARNWGQNWHSSLDLIGQSLSFEVTLKGGKTIASYDVAPPYWRFGMTYQGKQFHS

>AtEXP21

MTYVECF MII MATWFMFISYSHG

ANVAAAPGTNGLDTAWYDARAAYYGDIHGGGTELEGACGYGDLNKHGYGLATAALSTALFNSGASCGACYEIMCSPNPQ
GCLSGSIKITATDLCPPGSAWCYLPNKHFDLSLPMFIKIAQVKAKMVPVRYRRVPCAKTGGVKFEVKGNPNILTILPYN
VGGAGDIIAVSAKGSKTAWVVMSRYWGQNWTTNVNLTGQSVSLRVTTSDGITKDFTDVMPASWGFGQTFDGKTNF

>AtEXP22

MIYVEFLMIIMVIWVVPMSYG

HGAMIGNAVEAPDVAEAPGINDPSKALDTNWYDARATFYGDIHGGDTQQGACGYGNLFRQGYGLATAALSTALFNDGYT
CGACYEIMCTRDPQWCLPGSVKITATNFCPANYSKTTDLWCNPPQKHFDLSLAMFLKIAKYKAGVVPVRYRRIPCSKTG
GVKFETKGNPYFLMVLIYNVGGAGDIKYVQVKGNKTGWITMKKNWGQNWTTITVLTGQGLSFRVTTSDGITKDFWNVMP
KNWGFGQTFDGRINF

>AtEXP23

MIYVEGFMMIMATLLVSMSYG

HRAMINDVAEAPVFDDVVSPNGLDSSWYDARATFYGDIHGGETQQGACGYGDLFKQGYGLETAALSTALFNEGYTCGAC
YQIMCVNDPQWCLPGSVKITATNFCPPDYSKTEGVWCNPPQKHFDLSLPMFLKIAQYKAGVVPVKYRRISCARTGGVKF
ETKGNPYFLMILPYNVGGAGDIKLMQVKGDKTGWITMQKNWGQNWTTGVNLTGQGISFRVTTSDGVTKDFNNVMPNNWG
FGQTFDGKINF

>AtEXP24

MELLKRKLYAKILMMVMVIWIAPMTNG

HDHASHVPGGRPGAHPSHGAHPAHGAHPSHGAHPSHGAHPSHGAHPSHGALPSHGGQVPHSGWGHGRATFYGDINGGET
QQGACGYGDLHKQGYGLETAALSTALFNNGSRCGACYEIMCEHAPQWCLPGSIKITATNFCPPDFTKPNDNWCNPPQKH
FDLSQPMFLKIAKYKAGVVPVKFRRVPCAKIGGVKFEIKGNPHFLMILPYNVGGAGAVRAMQIKGTRTQWIAMKKNWGQ
IWSTGVVLTGQCLSFRLTTSDGVMKEFIDVTPPDWKCNGQSFDGKVNF

>AtEXP25

MKLLEQMVYVECFMIIMATLLVSMSYG

HRAMINDVAEAPVIDNVGSPTNGLDSSWYDARATFYGDIHGGETQQGACGYGDLFKQGYGLETAALSTALFNEGYTCGA
CYQIMCVHDPQWCLPGTIKITATNFCPPDYSKTEGVWCNPPQKHFDLSLPMFLKIAQYKAGVVPVKYRRISCARTGGVK
FETKGNPYFLMILPYNVGGAGDIKLMQVKGDKTGWITMQKNWGQNWTTGVNLTGQGISFRVTTSDGVTKDFNNVMPNNW
GFGQTFDGKINF

>AtEXP26

MKLLEKMIYVEFLMIIMAMWVVPMSYG

HGAMIGNAVEAPDVAEAPGINDPSKALDPNWYDARATFYGDIHGGDTQQGACGYGNLFRQGYGLATAALSTALFNDGYT
CGACYEIMCTRDPQWCLPGSVKITATNFCPANYSKTTDLWCNPPQKHFDLSLAMFLKIAKYKAGVVPVRYRRIPCSKTG
GVKFETKGNPYFLMVLIYNVGGAGDIKYVQVKENKTGWITMKKNWGQNWTTSTVLTGQGLSFRVTTTDGITKDFWNVMP
KNWGFGQTFDGKINF

>AtEXPB1

```
MQLFPVILPTLCVFLHLLISGSGS

TPPLTHSNQQVAATRWLPATATWYGSAEGDGSSGGACGYGSLVDVKPFKARVGAVSPILFKGGEGCGACYKVRCLDKTI
CSKRAVTIIATDQSPSGPSAKAKHTHFDLSGAAFGHMAIPGHNGVIRNRGLLNILYRRTACKYRGKNIAFHVNAGSTDY
WLSLLIEYEDGEGDIGSMHIRQAGSKEWISMKHIWGANWCIVEGPLKGPFSVKLTTLSNNKTLSATDVIPSNWVPKATY
TSRLNFSPVL

>AtEXPB2

MTILVVDRYYML  MNLLFALTCLLLNLTHC

FSPKKFNISAATTSDSDWSIAGSTWYGNPTGYGSDGGACGYGNAVAQPPFSKMVSAGGPSLFKSGKGCGACYQVKCTSK
SACSKNPVTVVITDECPGCVKESVHFDLSGTAFGAMAISGQDSQLRNVGELQILYKKVECNYIGKTVTFQVDKGSNANS
FAVLVAYVNGDGEIGRIELKQALDSDKWLSMSQSWGAVWKLDVSSPLRAPLSLRVTSLESGKTVVASNVIPANWQPGAI
YKSNVNF

>AtEXPB3

MQLFPVMLATLCIVLQLLIGSSA

LATTNRHVSNSHWLPAVATWYGSPNGDGSDGGACGYGTLVDVKPLHARVGAVNPILFKNGEGCGACYKVRCLDKSICSR
RAVTVIITDECPGCSKTSTHFDLSGAVFGRLAIAGESGPLRNRGLIPVIYRRTACKYRGKNIAFHVNEGSTDFWLSLLV
EFEDGEGDIGSMHIRQAGAREWLEMKHVWGANWCIIGGPLKGPFSIKLTTLSAGKTLSATDVVPRNWAPKATYSSRLNF
SPVL

>AtEXPB4

MASSQRYFALLALFAVSLKFCYC

QNETIDVAGSGTAGVTWYGEPFGAGSTGGACGYGSAVANPPLYAMVSAGGPSLFNNGKGCGTCYQVVCIGHPACSGSPI
TVTITDECPGGPCASEPVHIDLSGKAMGALAKPGQADQLRSAGVIRVNYKRAACLYRGTNIVFRMDAGANPYYISFVVE
YENGDGDLSNVEIQPAGGSFISMQEMRSAVWKVNSGSALRGPFNIRLTSGESHKVIVAYNVIPANWKPDESYRSIVNF
>AtEXPB5
MASSSLKCFSFIVVLTTFFAISLKPCYC
HNKTHWNTAGITWYGDREGPGTTGGACGYGDAVAKHPYRCMVSAGGPSLFKDGKGCGACYRLKCDHPLCTKKPIKVMIS
DECPGCTKESVHFDLSGKAFGALAKRGKGDQLRNLGELKVSYKRACCKHPKTMIAIHVDAGANPYYMSFAVKFANGDGN
FACIEVQPAGGQYMKMEEMRSAVWRLSPGVPLKGPFNIRLTSAVSGKKIIAKGVIPEKWSPGAIYHSKVNFPVQRKQK

>AtEXPL1
MGSFLFLIVVIFLFSSSVNA
CDRCLHRSKAAYFSSASALSSGACAYGSMATSFFAGHIAAAIPSIYKDGAGCGACFQVRCKNPKLCSTKGTIVMITDLN
KSNQTDLVLSSRAFRAMAKPIVGADKDLLKQGIVDIEYQRVPCDYGNKNMNVRVEEASKKPNYLEIKLLYQGGQTEVVS
IDIAQVGSSPNWGYMTRSHGAVWVTDKVPTGAIQFRFVVTGGYDGKMIWSQSVLPSNWEAGKIYDAGVQITDIAQEGCD
PCDAHIWN

>AtEXPL2
MLQGFLFLLSVVLLFSSSAAA
CDRCLHSSKAAYFSSASALSSGACAYGSMATGFFAGHIAAALPSIYKDGSGCGACFQVRCKNPTLCSSKGTTVIVTDLN
KTNQTDLVLSSRAFRAMAKPVVGADRDLLKQGIVDIEYRRVPCDYGNKKMNVRVEESSKNPNYLAIKLLYQGGQTEVVA
IYIAQVGSSHWSYMTRSHGAVWVTDKVPNGALQFRFVVTAGYDGKMVWSQRVLPANWEAGKSYDAGVQITDIAQEGCDP
CDDHIWN

>AtEXPL3
MRSFLYLIVVIFLFSSSVNA
```

CDRCLHRSKASYFSSASALSSGACAYGPMATSFFAGHIAAAIPSIYKDGAGCGACFQVRCKNPKLCNSKGTIVMVTDLN
TSNQTDLVLSSRAFRAMAKPVVGVDKYLLKQGIVDVEYQRVPCNYGKRNLNVRVEEASKKPNYLAIKLLYQGGQTEVVG
IDIAPVGSSQWSYMSRSHGAVWATDKVPTGALQFKFTVTGGYDGKTVWSKRVLPANWNSGRIYDAGVQITDIAQEGCDT
CGHIWN

>AtEXLB
MKHSHVLLLLFVQVIVLLPLLCLS
DDFVNSRATYYGSPDCKANPRGHCGYGEFGRDINNGEVSGVSWRLWNNGTGCGACYQVRCKIPPHCSEEGVYVVATDSG
EGDGTDFILSPKAYGRMARPGTENQLYSFGVVNVEYQRIPCRYAGYNLVYKIHEKSYNPHYLAILVLYVGGVNDILAVE
VWQEDCKEWRRMRRVFGAVHDLQNPPRGTLTLRFLVYGSAGINWIQSPNAIPADWTAGATYDSNILLT

>GbEXPA1
MGLVNDLWYLLFILIAQLFQCDAKQRWSNAHATFYGGSTYAESMGGACGYGSAEEYGRNTAALSTALFNNGQACGSCYE
IKCSDDPMWCLKGSVKVTATN
FCPPNNALPNDNGGWCNPPLQHFDMSQAAFTTIALYHAGIVPVLYRRVHCARKGGIRFSINGNKYFNLVLISNVGGRGD
VQRVWMKGSKTQWIEMKRNWG
QNWESGENVCGQSLSFRITVSNGKSATSYNVAPSNWQFGQTFVGKQL*
>GbEXPA2
MAMPSQHGRLMMGIITLALAMAMAGLKSCTATSDEWSHGHATFYGGSDASGTMGGACGYGDLYSSGYGTKTAALSTALF
HNGASCGECFLIKCDVAADSQ
WCLPGKSITITATNFCPPNYDLPSNDGGWCNPPLQHFDMAEPAWEQIAIYRGGIVPVLFKRVPCKKSGGVRFTINGRSY
FDLVLISNVGGSGVISAVSIK
GSRTEWQAMSRNWGANWQSNSYINGQSLSFRITTSDGKTITFPNVAPSNWQFGQTFSTSLQF*
>GbEXPA3
MAPATQSITLIFTLLVFLWLISSALGYDGWQTAHATFYGGSDASGTMGGACGYGNLYSQGYGTNTAALSTALFTNGLTC
GACYEMKCNDDPQWCLPGTVV
VTATNFCPPNNALPNDNGGWCNPPLQHFDMAEPAYQQIAKYSSGIVPILFRRVPCLKKGGIRFTVNGHSYFNLVLVSNV
GGAGDVHAVWIKGSKTGWQPM
SRNWGQNWQSNSNMNGQSLSFKVTTSDGRITVSYNVAPSNWEFGQTFQGTQF*
>GbEXPA4
MKENCLTVSRFYVQLNGTCNTEHNTYIHIEARGYGNLYGQGYGTNTAALSTALFNNGLTCGAYYEMKCNDDPQWCLPGT
VVVTATNFCPPNNALPKHNGG
WCNPPLQHFDMAEPAYQQIAKYSSGIVPILFRRVPCLKKGGIRFTVNGHSYFNLVLVSNVGGAGDVHPVWIKGSKTGWQ
PMSRNWGQNWQSNSNMNGQSL
SFKVTASDGRTTVSYNVAPSNWEFGQTFQGTQF*
>GbEXPA5
MIEILCIPDSRGLGLQILLFVGMEEFRKRCVLKFEPEMASLGFILLGFLSLARFVLVQSWTNAHATFYGGSDASGTMGG
ACGYGNLYSQGYGTNTAALST
ALFNGGLSCGACYEVKCSGDPQWCLPRSIVVTATNFCPPNFALPNNAGGWCNPPLQHFDLSQPVFQNIAKYRAGVVPVV
YRRVPCHRKGGIRYTINGHSY
FNLVLITNVGGAGDVHSVSIKGSKTGWQPMSRNWGQNWQSNTYLNGQSLSFKVTTSDGRTVTSYNAAPANWQFGQTFSG
AQFH*
>GbEXPA6
MALIYSISLTLLLEFLWLAPRIYGYVDGWQTAHATFYGGSDASGTMGGACGYGNLYSQGYGTNTAALSTALFNNGLSCG
ACYEMKCNDDPQWCLPGTVVV
TATNFCPPNNALPNNNGGWCNPPLQHFDMAEPAYQQIAKYRSGIVPIVFRRVPCLKKGGIRFTINGHSYFNLVLVSNVG
GAGDVQAVWIKGSRTGWQPMS
RNWGQNWQSNSYMNGQSLSFKVTTSDGRTTVSYNAAPSNWQFGQTFQGTQF*
>GbEXPA7
MKENFLTVSRFYVQLNGTCNTEHNTYIHIVGLLWLISSALGYDGWQTAHATFYGGSDASGTMGGACGYGNLYSQGYGTN
TAALSTALFNNGLTCGACYEM
KCNDDPQWCLPGTVVVTATNFCPPNNALPNDNGGWCNPPLQHFDMAEPAYQQIAKYSSGIVPILFRRVPCLKKGGIRFT
VNGHSYFNLVLVSNVGGAGDV
HAVWIKGSKTGWQPMSRNWGQNWQSNSNMNGQSLSFKVTTSDGRTTVSYNVAPSKWEFGQTFQGTQF*

>GbEXPA8
MALVQSIALVASLGFLWLLPRAYGYDGWQTAHATFYGGSDASGTMGGACGYGNLYSQGYGTNTAALSTALFNNGLSCGA
CYEMQCNDDPQWCLPGTVVVT
ATNFCPPNNALPNDNGGWCNPPLQHFDMAEPAYQQIAKYRSGIVPILFRRVPCLKKGGIRFSINGHSYFNLVLVSNVGG
AGDVQAVWIKGSRTGWQPMSR
NWGQNWQSNSYMNGQSLSFKVTTSDGRTSVSYNVAPSNWQFGQTFQGTQF*
>GbEXPA9
MALLQSMVLTTLLGFLLLLPRAYGYDGWQTAHATFYGGSDASGTMGGACGYGNLYNQGYGTNTAAVSTALFNNGLSCGA
CYEMKCNDDPQWCLPGTVVVT
ATNFCPPNNALPNNNGGWCNPPLQHFDMAEPAYQQIAMYRSGIVPILFRRVPCLKKGGIRFTINGHSYFNLVLVSNVGG
AGDVHAVWIKGSKTGWQPMSR
NWGQNWQSNSYMNGQSLSFKVTTSDGRTAVSYNAAPSNWQFGQTFQGTQF*
>GbEXPA10
MADLAAYCTYNCLQEHFPAVGVKTSQMRMESWKVVRNATKPSNWARDPSPDEILVPTRANIRVLLATAAGLSTTWHWFT
GAPAHVQVAPSKALEHVHLIR
TKRLNSMALIESIPLITVLVGLLWLLPRAYGYDGWQTAHATFYGGSDASGTMGGACGYGNLYSQGYGTNTAALSTALFN
NGLTCGACYEMKCNDDPQWCL
PGTVVVTATNFCPPNNALPNDNGGWCNPPLQHFDMAEPAYEQIAKYRSGIVPILFRRVPCLKKGGIRFTINGHSYFNLV
LVSNVGGPGDVHAVWIKGSKT
GWQPMSRNWGQNWQSNSYMNGQSLSFKVTTSDGRTTVSYNVAPSNWQFGQTFQGTQF*
>GbEXPA11
MGFMCLAFPTLGYDGWETAHATFYGGSDASGTMGGACGYGNLYSHGYGTNTAALSTALFNNGLSCGACYEMKCNDDPQW
CLPGTVVVTATNFCPPNNALP
NDDGGWCNPPLQHFDMAEPAFLHIAKYRSGIVPILFRRVPCLKKGGIRFTINGHSYFNLVLVSNVGGAGDVHAVWIKGS
RTGWQPLSRNWGQNWQSNSYM
NGQSLSFKVTTSDGRTSVSYNVAPSNWQFGQTFQGTQF*
>GbEXPB1
MFAGVSVEAIYFIVIGATLILSSKSVVCIHLKAYGLQEDGTDWQSATATWYGSPDGDGSDGGACGYGDLVKQVPFASKI
AAGSPVLFKGGKGCGACFQVK
CTDNGLCSETPVTVVITDECPGGAYCGTGKTHFDLSGTAFGAMGAPAKASDLRNTGVVPILFRRVPCEYPGKNIAFHVN
EGANSNWFEVLIEYEDGDGDL
AAVDLKQANSDAWQGMQQVWGANWCLNGGPLKAPFSIRLKSLSSGRTLAANNAIPENWQPSATYRSLVNYND*
>GbEXPA20
MAGFPLLKFVLFLLGFISLVGLAKGSRRNLGWMSAHATFYGGSDASGTLGGACGYGNLYKQGYGIRNAALSTVLFNKGL
TCGACFEIKCNARLDSQWCVR
GNPSIIVTATNFCPPNKALPNNNGGWCNPPLRHFDMSQPAYQHIALYRAGIVPVLYRRVPCKRRGGIQFTLNGWPYFTL
VLITNVGGAGEVHRVWIKGSR
TRGWQPMTRNWGQNWQTNILFSGQSLSFIVTTSDGKSVTSYNVAASNWCYGQTFTGKQF*
>GbEXPA21
MRRLRQGLVLLSIAYLNQIVSGSLQSKDGWNYGRATFYGDPDALGKMAGACGYGDLYREGYGLSTAALSTVLFNKGQSC
GACFELRCVDDPLYCHHGTSI
VITATNFCPPNYGLPSDAGGWCNPPREHFIMPVTAFEKIAMWKIGNMNVEYRRVKCQREGGMRFKIAGKDYFYMVLITN
VGGSGEVVAVKMKGSRTGWLP
MGRNWGQNWHLNTNLKGQPLSFEVTTSDGRTVTSYNVAPPNWEFGQSFVGKQF*
>GbEXLB1
MASSMMLACCFLVALLIQKVFSACENCNFVTSRATYYGSPDSEGTAGGACGYGSYGRNLNGGDVACVAQLYRNGAGCGA
CYQVKCTNEDLCTEDGVNIVV
TDYGEGDRTDFILSYRGYTKLARSGKANELVAMGVVAIQFKRVSCQYPGTNIMFKIDENSNFPGYLAFTFWFQGGQKDV
SAVHVCKDDTLECKLSRNYGA
VWNVMEPPKGALSVRFLVSDGDEEQWMTAPNVIPANWTKGALYDSGIQIN*
>GbEXLA1
MFKSDHMEMYRANILWFMLLMIPAALACDRCLHKSKVAYFAPSHAVNAGACGYGSFGASLKGGFVAAANPKIYRRGVGC
GACYQIRCTNSALCTKSGVKI
VVTDLNQNNQTDFVLPSRSFSSLALPNKASQLVKMGAVDVEYKRIICEYPGQNMTVKVDKSSNYPYYLAVQFLYQGGQT

DITIVDVAQVGSTDWHYMTRN
HGAVWDIQKPPNGPLSFRLMVTGGYDGSWVWPKRNLLPANWRPGAVYDSGVQISEIAQEGCYPCETGDWGHDQ*
>GbEXLA2
METFTRTVCLILIFIVVTVFAGDPCYDKGKVSYYPSKHSMDAGACRYGTYGATVNGGHVAGASAKFYGGGAICGACYEI
RCTNSALCTKTGVKVVVTDLN
QSNQTDFVLPFRTFCSLARTGRAAALVKEGIVDVEYKRIFCEYRGHNLTINIEESSKYPYYLALKFLYQGGQTQILTVA
IYKAGFSDLHYMTRTNSAVWA
LENPPEGSLSLQIAVTSGFDPMWLSKDNVLPAKWKAGDLFDSGFQITAIDKGATRPCQSEDKKKFSM*
>GbEXLB2
MASQCFSGGLIFLIMVQAVFCCGDCYVTSRATYYGSPDCLGTAGGACGYGSFGKNLNAGNVAAVSQLYRNGTGCGGCYQ
VRCTNEDLCTDDGVKIAITDY
GQGDETDFILSSRGYSMLARSEKAKELIAAGVVDIEYKRVSCEYPGYNLMFKIDESSSFPDYFALLFWYQAGQKDVLAV
ELCQEETYEWKAMCRNHGAVW
DVITPPKGALSLRFLVSDGYQQEWISAANVIPANWKAGSVYDSGIQLSY*
>GbEXLB3
MASQCFSGGLIFLIMVQAGFCCGDCYVTSRATYYGSPDCLGTAGGACGYGSFGKNLNAGNVAAVSQLYRNGTGCGGCYQ
VRCTNEDLCTDDGVKIAITDY
GQGDETDFILSSRGYSMLARSEKAKELIAAGVVDIEYKRVSCEYPGYNLMFKIDESSNFPDYFALLFWYQAGQKDVLAV
ELCQEETYEWKAMCRNHGAVW
DVITPPKGALSLRFLVSDGYQQEWISSANVIPANWKAGSVYDSGIQLSY*
>GbEXPA12
MQITVVEGWTAAHATFYGGSDASGTMGGACGYGNLYSTGYGTNTAALSTALFNNGQTCGACFQIKCDAAKVPQWCLSGR
SITITATNFCPPNNALPNDNG
GWCNLPLQHFDMSQPAFQQIAMYKAGIVPVVYRRVPCKKMGGIRFTINGLAYFDLVLISNVGGRGDVKAAWIKGSNTGW
QQMQRNWGANWQSNSYLNGQS
LSFRVTTSNGRTLTMYNVAPANWEFGQTFTTSRQF*
>GbEXPA13
MGYYSKESILGFICLVFFSLARLVQASEWSNAHATFYGGSDASGTMGGACGYGNLYSQGYGTETAALSTALFNNGMSCG
ACYELQCNDDPQWCLPGSIVV
TATNFCPPNYALPNNAGGWCNPPLQHFDLSQPVFLKIAEYRAGIVPVIYRRVPCEKKGGIRFTINGFSYFNLVLITNVG
GAGDVSAVSIKGSKTGWQPMS
RNWGQNWQSNSYLNGQRLSFKVTTSDGRSVTSYNVVPANWLFGQTFTSGAQFH*
>GbEXPA14
MGSFRLVLFWMAVAMNCFNHLLVNGDHDNEGWNYAHATFYGGADASGTMAGACGYGNLYSQGYGTTTAALSTVLFNNGQ
TCGACFELRCVDDPQWCLSGS
PSVTVTATNFCPPNYALPNDDGGWCNPPREHFDMSMPAFEKIAIWKAGIVPIQYRRVKCEKQGGIRFTINGNYYFYLVL
ITNVGVDGDVVAVEIKGSRTG
WMSLSRNWGQNWQCNSNLMGQSLSFKVTTSDGRSLTSYDVSPPDWNFGQTFEGKQF*
>GbEXLB4
MTSRILKLVPGVVLVLFMLNGVLSCDDCYIKSRATFYGSVDYLGTPGGACGYKSFGRNLNGGDVAAVAFLYRDGAGCGA
CYQIRCTDPNLCNSEGINVVV
TDFGHGDRTDFILSPSAYSKLAINGKSIELVASGVVEIEFRRISCRYPGYNIMFKVDENSKYPDYLALNIWYQGGRKDI
VAVEVSLEGSNQWKPCRRSHG
AVWDIVSPPRGALSVRFLVTDEHQYKEPLPISSPEYRWRMKRERDRSEPWERSMQMGAKKNCIHHAIADSLLEYYRSAT
RSCAPMKSLAGSGWFFDAAAG
RMTHLFCGSDDEEQEEGMTDVFLHDVNTRVEKMYANEFLCKRNLVGSSGRLWHFMIAPLNALGKKISNGFCDP*
>GbEXLB5
MSSRMYILVLFMSIHSVFGCDDCYVKSRATYYGSTDCLGTVGGACGYGVFGRNLNAGDVAGVAQLYRNGTGCGACYQLR
CTNEDLCTGEGNKIVVTDYGQ
GDRTDFILSLRAYSRLARAGKSYDLVTSGVVEIEFKRISCSYPGYNLMFKVDESSNYPDYLALLFWYQAGRRDMIAVEL
SCDQGWKAMCRNHGGVWDMVI
PPRGALYVRFLMSDGYKSEWVYAKNPIPANWGPGTVYDSGIQTSD*
>GbEXPA15
MALRLIVAALFALACRVKGEGGDYDSGGVWSSAHATFYGGGDASGTMGGACGYGNLYSTGYGTNTAALSTALFKNGEAC

GSCFQIKCNVQEDPQWCLDGA
PSVVVTATNFCPPNNALPNDNGGWCNPPLQHFDLSQPAFQQIARYSGGIVPVSFRRLKCVKKGGIQFTINGHTYFNLVL
VSNVGGSGDLKSVWIKGSKTG
WLSMQRNWGANWQSNEYLDGQSLSFRVTTGDGYTATSFDVAPADWKYGQSFEGNQIL*
>GbEXLA3
TLIVMFFILSFGPLLYCFSCKEPSSKLIYYLSHCIILVNGACGYGHLGATLYGGNVGVYRKGVGCGACYQIRCTDPQLC
NKIGTKIVVTGVSHNNKTDFV
VTQRAFVNMAAPDKDDQLLKAGIVGLEYKRIPCQYPGRNLSVNVDKSSRFPSYMAIQFLFQGDQPEIVAVDVAEVASPN
WHYVHRNHGAVWDISDPPTGP
LQFRLVITIGYDGKGIWAKHALPANWKPGVIYDTLAQIHDIAQEACGPCETSNW*
>GbEXLA4
MATPVSGFVKEKAALTACLTLLLIVNTALGCDRCVHQSKVAYYAYKGAVNVGACGYGSSGGTLYDGKVGAVSRTMFRKG
AGCNACYQIRCIDPKLCRNMG
EKVMVIDFTENDQMDFVVTTQTFSNLALPEKRDELSNKGIVDIEYKRIVCEYMGQNLSVKVDERSNYPSYLVVQFWKQG
GQTDIEAVDVAQVGSYSWSNM
KRNFGGVWDIDTPPIGPLQFRVLVTSGYDRKWVSSKYVLPSDWKEGIVYDSGVQIEDIAQESCNPCDTSNW*
>GbEXLA5
KVALTPKRWAFLTACLTLLLIANSALGCDRCVHKSKVAYYANRGAVNVGACGYGSLGATLCDGNVAAVSSTMYRKGVGC
NACYQIRCINSKICSKSGAKV
IVIDLTENNQTDFVVTTRTFSSLALPKKGHALVKMGIVDIEYKRILCEYMGQNFSVKVDESSNYPYYLAVQFLYQGGQT
DIVAVDVAQVGSSNWHYMNRN
HGGVWDINKPPKGPLQFRFLITGGYDGKWVWSKYVLPSDWKIGIIYDSGVQIEDIAQESCSPCDTSNX
>GbEXPA16
MELIVGADFIRSIAAAGRPDLVDLSQADFEDIFIMASCNMGVIWFFFLSFAGLVQASDWINAHATFYGGGDASGTMGGA
CGYGNLYNQGYGTETAALSTA
LFNNGMSCGACFEIQCTDDPQWCLPGSIVVTATNFCPPNDGLPNDAGGWCNPPLHHFDLAEPVFQKMAKYHAGIVPVVY
RRVPCEKKGGIRFTINGNPYF
NLVLISNVGGAGDVIGVSIKGSNTGWQPMSRNWGQNWQSNSHLTAQSLSFQVTASDGSTVNSYNVAPSDWQFGQTFEGA
QFN*
>GbEXPA17
MYSKMALAKILVTSFLLAFLWVSPGAHGDGGWRAAHATFYGGNDASGTMGGACGYGNLYSQGYGTNTAALSTALFNNGL
SCGSCYEIKCNEDPQWCLAGT
VTVTATNFCPPNNALPNNNGGWCNPPLRHFDMAVPAYLHIAKYSSGIVPIIFRRVPCERKGGIRFTINGHSYFNLVLVS
NVGGAGDVHAVSIKGSNTGWQ
PMSRNWGQNWQSNSYLNGQSLSMQVTTSNGRTVTSHNVAPPDWQFGQTFEGAQF*
>GbEXPA18
MGSLSRATILALMALLCMHLQEEVMATESNNNDGWSHAHATFYGGSDASGTMAGACGYGNLYSQGYGTTTAALSTALFN
NGQACGACFEIRCVDDPQWCL
QGSPSVVITATNFCPPNYALPSDDGGWCNPPRLHFDMAMPAFERIAVWKAGIVPVQFRRVKCEKDGGLRFTINGNYYFY
LVLITNVGSDGDVVGVKIKGS
RTGWVPMSRNWGQNWQCSTNLMGQSLSFEATTSDGRTVTSYNVAPNHWQFGQTFEGKQY*
>GbEXPA19
MMTKMNTLLLVVAMVCVIWIESSTVVEALARNGWNRGHATFYGSTNAVATMGGACGYDNVYTSGYGIYTAALSSALFRE
GAACGACFEVMCDARSDPQWC
IRGRRITITATNFCPANTDGGPGWCNPPLQHLDMSMPAFNRIARGYKEGVVGILYRPAGCAKKGGVRFTIKGHSYFNLV
LITNVGGSGEVRAVWVRGSGS
NTPWQAMRRNWGANWQSNAFLDRLSLSFRITAGDGKTLTFNNAVRSNWRFGQTFATHQQF*
>GbEXPB2
MAKAIRVEWPMMPNVVINLTGACFMFLFLLKEVETLAFLDQQDLDADQLEWRPATATWYGSPSGDGSDGGACGYGNLVD
QKPLKARVGAVSPVLFKGGEG
CGACYKVKCMDKSICSDRPVTVIITDECPGGYCAFGRTHFDLSGAAFGRMATAGKTPALLNTGELPVLYRRTLCEYPGK
NITFHVNEGSTDYWFSILIEY
EDGDGDVGAVHLKEAGSEAWMEMKHVWGANWCLLGGPMKGPFSLRVTSLSSHRTLSARNVIPQHWLPNATYKSRLNFDD
*

>GbEXPB3
MTKFLLLDVVSLLLAAFLFWVATHALPLLHQSQHIEETDDWQQATATWYGSPNGDGSNGGACGYGSLIQQMPYGSKVGA
GSPVLFRGGKGCGECYKVKCM
NNDVCTEKPVTIVITDECPGGYCAFGRTHFDLSGTAFSDMAATGKVSALLNVGELPVLYTRTSCEYPGKKIAFHVNEGA
TDHWFSVVVEYEDGDGDVGAM
DLMQAGSNSWQAMSQVWGANWCLNDGPLKAPFSIRLRSLSTARSLRAKNVIPEKWQPNATYRSLVNFDD*
>GbEXPB4
MKTFGCLRWQLSPSTAWLVALAVVITLFIKPTLGVDRELIDADEFQWRPATATWYGSPDGDGSNGGACGYGNLVDVKPM
RKRVGAVSPVLFKGGEGCGAC
YKVKCMDESICSDRPVTVIITDECPGGYCAFGRTHFDLSGAAFGRMATTGKTPELLNTGELSVLYRRTLCEYPGKNITF
HVNEGSTDYWFSILIEYEDGD
GDVGAVHLREAGSEAWMEMKHIWGANWCLLGGPMKAPFSLRVTSLSSHKTLSARNVIPQHWLPNATYKSRLNFDD*
>GbEXPA22
METLLLLCSVVFSYIYMQFLALKGPCNNSNWSFTAREDMKKESSLMILAVMLTFQSTASVRGYIPIGGWDEAHATYYGG
NDASGTNNGACGYGNQLSAGY
GTITTALSTPLFEQGKTCGACYELRCFGGEAEGCLYGSPPVIVTATNFCPPGSFGGWCDPPNVHFDLSEPAFSHLARVP
VGHVRVGYTRVNCERSGGMRF
TINGHTYFNQVLVTNVGGAGDVEQVWIRGSNSGWMPMVRNWGQNWLNEGDMDGQKLSFLVTTSDGRTATSYDVAPPNWQ
FGQTFSGTQVLSH*
>GbEXPA23
MASSVENYNNVAMATLLGFMWLLVRSVYGDSGWESAHATFYGGSDATGTMGGACGYGNLYSQGYGTNTAALSTALFNNG
LSCGACYEMKCNDDPQWCLPG
TVMVTATNFCPPNNALPNDNGGWCNPPLQHFDMAQPAYQQIAKYRSGIVPIMFRRVACVRKGGVRFTMNGHSYFNLVLI
SNVGGAGDVHAVSIKGSNTGW
QPMSRNWGQNWQSNSYLNGQSLSFQVTTRDGRTITSYNVAPSNWQFGQTFQGAQF*
>GbEXPA24
MAQAVHGWTNAHATFYGGGDASGTMGGACGYGNLYSQGYGTNTAALSTALFNNGLSCGACYEIRCNDDPQWCLHNTIVV
TATNFCPPNNALPNNDGGWCN
PPLQHFDLSEPVFQHIAKYRAGIVPVMYQRVPCHKRGGIRFTINGHSYFNLVLITNVGGAGDVHAVSIKGSRTGWQPMS
RNWGQNWQSNSYLNGQSLSFK
VTTSDGKTVTSYNVAPSNWQFGQTFSGRQIY*
>GbEXPA25
MASFEQSYVILVTLLGLLWMLSRPVYGDSGWESAHATFYGGSDASGTMGGACGYGNLYSQGYGTNTVALSTALFNNGLS
CGACYEMKCNDDPQWCLPGTV
TVTATNFCPPNNALPNDNGGWCNPPLQHFDMAQPAYQQIAKYRSGIVPIMFRRVACVRKGGVRFTMNGHSYFNLVLISN
VGGAGDVKAVSIKGSNTGWQP
MSRNWGQNWQSNSYLDGQSLSFQVTTSDGRTTTSYNVAPSNWQFGQTFEGAQF*
>GbEXPA26
MASFEQSYVILVTLLGLLWMLSRPVYGDSGWESAHATFYGGSDASGTMGGACGYGNLYSQGYGTNTAALSTALFNNGLS
CGACYEMKCNDDPQWCLPGTV
TVTATNFCPPNNALPNDNGGWCNPPLQHFDMAQPAYQQIAKYRSGIVPIMFRRVACVRKGGVRFTMNGHSYFNLVLISN
VGGAGDVKAVSIKGSNTGWQP
MSRNWGQNWQSNSYLDGQSLSFQVTTSDGRTSTSYNVAPSNWQFGQTFEGAQF*
>GbEXPA27
MASFEQSYVTLVTLLGLLWKLSRPVYGDSGWQSAHATFYGGSDASGTMGGACGYGNLYSQGYGTNTAALSTALFNNGLS
CGACYEMKCNDDPQWCLPGTV
TVTATNFCPPNNALPNDNGGWCNPPLQHFDMAQPAYQQIAKYSSGIVPIMFRRVACVRKGGVRFTMNGHSYFNLVLINN
VGGAGDVQAVSIKGSNTGWQP
MSRNWGQNWQSNSYLNGQSLSFQVTTSDGRTTTSYNVAPSNWQFGQTFEGAQF*
>GbEXPA28
MASFEQSYVTLVTLLGLLWMLSRPVYGDSGWQSAHATFYGGSDASGTMGGACGYGNLYSQGYGTNTAALSTALFNNGLS
CGACYEMKCNDDPQWCLPGTV
TVTATNFCPPNNALPNDNGGWCNPPLQHFDMAQPAYQQIAKYRSGIVPIMFRRVACVRKGGVRFTMNGHSYFNLVLISN
VGGAGDVKVVSIKGSNTGWQP

MSRNWGQNWQSNSYLDGQSLSFQVTTSDGRTTTSYNVAPSNWQFGQTFEGAQF*
>GbEXPA29
MAACTADKIVSATLLGFFLLARAACADSGWQSAHATFYGGSDASGTMGGACGYGNLYSQGYGTNTAALSTSLFNNGLSC
GACYEMQCNDDPQWCLPGTVM
VTATNFCPPNNALPNDNGGWCNPPLQHFDMAQPAYQQIAKYRSGIVPIMFRRVACVRKGGVRFTMNGHSYFNLVLISNV
GGAGDVHAVSIKGSNTGWQPM
SRNWGQNWQSNSYLNGQSLSFQVTTSDGRTITSYNVAPSNWQFGQTFQGAQF*
>GbEXPA30
MASSVESNIVAMATLLGFMWLLVRPVYGDSGWESAHATFYGGSDASGTMGGACGYGNLYSQGYGTNTAALSTALFNNGL
SCGACYEMKCNDDPQWCLPGT
VMVTATNFCPPNNALPNDNGGWCNPPLQHFDMAQPAFQQIAKYRSGIVPIMFRRVACVRKGGVRFTMNGHSYFNLVLIS
NVGGAGDAQSVSIKGSNTGWQ
PMSRNWGQNWQSNSYLNGQSLSFQVTTSDGRTTTSYNVAPSNWQFGQTFEGAQF*
>GbEXPA31
MAQFLQTWGWTNAHATFYGGSDATGTMGGACGYGNMYSQGYGTNTAALSTALFNNGLSCGACYEIKCNDDPQWCLSNTI
VVTATNFCPPNTALPNNAGGW
CNPPLLHFDLSEPVFQHIAKFRAGIVPVMYQRVPCQKKGGIRFTINGHSYFNLVLLTNVGGAGDVNAVSIKGSGTGWQP
MSRNWGQNWQSNSNLNGQSLS
FKVTTSDGQTVTSYNAAPSNWQFGQTFSGAQLGN*
>GbEXPA32
MVKEIRVKRGGEVSGQSSSFIFVCSDDRIFDCSIRFLYICFSWPHTAGRHPQKTKTLKGKEQKNKREHFNCVVRCVVIA
SIKEEKNKVKGIEKETQLEGF
RWSAAITATRMMEYLLAVLMLLISGTSSCMAYGTRGRSFYRAGPWMQAHATFYGGSDASDTMGGACGYGNLYSQGYGVE
TAALSTALFNDGRSCGACFEI
KCYDDPQWCLPGRPSIYVTATNFCPPNYALPNDNGGWCNPPRPHFDLSQPIFLKMAEYRAGIVPVQYRRIPCVKKGGIR
FTINGFKYFNLILITNVGGAG
DVVKARVKGSRTGWMAMSRNWGQNWQCDTVLVGQSLSFSVTTSDGRTLTSYNVSPANWQFGQTFSGRQFF*
>PtEXPA1
MAMSSLICIATSLLIIMSSLWMAEARIPGVYSGGAWQNAHATFYGGADASGTMGGACGYGNLYSQGYGVSTAALSTALF
NNGLTCGSCFEIKC
ASDPRWCHSGSPSIFITATNFCPPNYALPSDNGGWCNPPRPHFDLAMPMFLKIAEYRAGIVPVAYRRVPCRKRGGIRFT
INGFRYFNLVLISN
VAGAGDIVQVSVKGSKTGWMSMSRNWGQNWQSNAVLVGQSLSFRVRASDRRSSTSWNIAPAHWQFGQTFTGKNFRV
>PtEXPA2
MAAQTFASCLLLFICLHLQSILADYGGWQGGHATFYGGGDATGTMGGACGYGNLYSQGYGTSTAALSTALFNNGLSCGA
CYEMRCDDDPKWCLP
GTITVTATNFCPPNPSLPNNNGGWCNPPLQHFDMAEPAFLQIAQYRAGIVPVAFRRVPCVKKGGIRFTINGHSYFNLVL
ITNVGGAGDVHSVSI
KGSKTGWQAMSRNWGQNWQSNNYLNGQSLSFQVTTSDGRTVTSYNAVPAGWQFGQTFEGGQF
>PtEXPA3
MALAGIFLVGFLAMVSSVYGYGGGGWINAHATFYGGGDASGTMGGACGYGNLYSQGYGTNTAALSTALFNSGLSCGSCY
EIRCVNDPKWCLPGSI
VITATNFCPPNNALPNNAGGWCNPPQHHFDLSQPVFQHIAQFRAGIVPVSYRRVPCRRRGGIRFTINGHSYFNLVLITN
VGGAGDVHSVSIKGSR
TGWQPMSRNWGQNWQSNNYLNGQSLSFKVTTSDGRTVVSYNAAPSSWSFGQTFSGAQFR
>PtEXPA4
MKMALAGFILVGLLAMVSSVNGYGGGGWINARATFYGGGDASGTMGGACGYGNLYSQGYGTNTAALSTALFNSGLSCGS
CYEIRCVNDPKWCLPGS
ILVTATNFCPPNNALPNNAGGWCNPPQHHFDLSQPVFQHIARYSAGVVPVSYRRVPCRRRGGIRFTINGHSYFNLVLIT
NVGGAGDVHAVSVKGSR
TGWQPMSRNWGQNWQSNNYLNGQSLSFKVTTSDGRTVVSYNAAPSSWSFGQTFSGAQFR
>PtEXPA5
MAMGSLLCFTTSLLIMMSLMWKGEARIPGVYSGGAWQTAHATFYGGADASGTMGGACGYGNLYSQGYGVSTAALSTALF
NNGLSCGACFEIKCADDP

QWCHSGSPSILITATNFCPPNYALPSDNGGWCNPPRPHFDLAMPMFLKIAEYHAGIVPVAYRRVPCRKRGGIRFTINGF
RYFNLVLISNVAGAGDIV
QVSVKGSKTGWMSMSRNWGQNWQSNAVLVGQSLSFRVRASDRRSSTSWNIVPAHWQFGQTFTGKNFRV
>PtEXPA6
MALLGFLLVGFLSSVSSVHGYYGGWINAHATFYGGGDASGTMGGACGYGNLYSQGYGTNTAALSTALFDNGLSCGSCFE
IRCVNDPQWCLPGVIVVTA
TNFCPPGGWCDPPNHHFDLSQPVFQHIAQYRAGIVPVIYRRIRCRRSGGIRFTINGHSYFNLVLITNVGGAGDVHSVAI
KGSRTGWQSMSRNWGQNWQ
SNSYLNGQSLSFVVTTSDGRSVVSYNVAPAGWSFGQTYSGGQYRY
>PtEXPA7
MTDRATISFISLVLLLSLVEARIPGVYTGAAWQSAHATFYGGNDASGTMGGACGYGNLYSQGYGVNTAALSTALFNNGL
SCGACFEIKCANEPQWCHSGS
PSIFITATNFCPPNYALPSDNGGWCNPPRPHFDLAMPMFLKIAEYRAGIVPVSYRRVPCRKPGGIRFTINGHRYFNLVL
ITNVAGAGDIVKVSVKGSRTG
WMSMSRNWGQNWQSNSVLVGQSLSFRVTGSDRRTSTSWNVVPSNWQFGQTFAGKNFRV
>PtEXPA8
MPPPLPQNPIHTLLKPLLLLLFIFPTATTSLTSSTTTPPPLSTTLSQWQSARATYYAASDPRDTVGGACGYGDLVKAGY
GMATVGLSESMFERGQICGACF
ELKCVDDLRWCIPGTSIIVSVTNFCAPNYGFTSDGGGKCNPPNKHFVLPIEAFEKIAIWKAANMPVQYRRIKCRKEGGI
RFNISGSGIFLSVLISNVAGAG
DVTAVKIKGSRTGWLDMGRNWGQNWHVNANLQNQALSFEVTSSDRMTVISYNVAPKDWRFGQAFEGKQFET
>PtEXPA9
MAPFGFLLVGFLSIVSSVHGYYGGWINAHATFYGGGDASGTMGGACGYGNLYSQGYGANTAALSTALFDNGLSCGACFE
IRCVNDPKWCLRGSIVVTATNFC
PPGGWCDPPNKHFDLSQPVFQHIAQYRAGIVPVIYRRHVKQQLTVVRCRKSGGIRFTINGHSYFNLVLITNVGGAGDVR
SVAIKGSRTRWQAMSRNWGQNWQ
SNAYLDGQSLSFLVTTSDGRRVVSYNVAPAGWSFGQTYSGGQFRY
>PtEXPA10
MECLGYTIVILLTMLKIVKGYGTGWTGAHATFYGGGDASGTMGGACGYGNLYSQGYGTNTAALSTALFNNGLSCGACYE
IKCVNDNIWCLRGSITVTATNFCPPNNALPNNDGGWCNPPQQHFDLSQPVFQKIAQYKAGIVPVQYRRVACRKSGGIRF
TINGHSYFNLVLITNVGGAGDVVAVSIKGTKSNWQAMSRNWGQNWQSNTYLNNQALSFKVTTSDGRTVVSNNVAPSNWA
FGQTYTGRQF
>PtEXPA11
MNFLGICLVVFLTMSKTVHGYGGGWSDAHATFYGGGDASGTMGGACGYGNLYSQGYGTNTAALSTALFKNGLTCGACFE
LKCVNDARWCLSGSIIVTATNFCPPNSAGGWCNPPSQHFDLSQPVFQHLARYKAGIVPVLYRRVACRKTGGIRFTINGH
SYFNLVLITNVGGAGDVVSVSIKGSRTGWQPMSRNWGQNWQSNSYLNGQALSFKVTTSDGRSIISSNVAPPNWAFGQTF
HGGQF
>PtEXPA12
MTMAATISLLLFALHLCLIGTYGDSGGWQGGHATFYGGGDASGTMGGACGYGNLYSQGYGTNTAALSTALFNNGLSCGS
CYEMRCDNDPKWCLPGSITVTATNFCPPNSALSNDNGGWCNPPLQHFDMAEPAFLQIAQYRAGIVPISFRRVPCVKKGG
IRFTINGHSYFNLVLITNVAGAGDVHSVSIKGSKTGWQAMSRNWGQNWQSNSYLNGQSLSFQVTTSDGRTVTSYNAVPE
DWQFGQTFSGGQF
>PtEXPA13
MAIKAMQDFVFVQLYLVLSFLLHGIYGDYGGWQVGHATFYGGSDASGTMGGACGYGNLYSQGYGTSTAALSTALFNNGL
SCGACFQIRCNNDPKWCHSGTITVTATNFCPPNYALSNDNGGWCNPPLKHFDLAQPAFLQIAQYRAGIVPVLFRRVPCV
KKGGIRFTINGHSYFNLVLITNVAGAGDVHAVSIKGSRTGWQTMSRNWGQNWQSNSYLNGQRLSFRVTTSDGKTVTWYN
VVPANWQFGQTFAGGQL
>PtEXPA14
MQFSVFLQLFLVFSLLFHGTCGDRGGWQIGHATFYGGSDASGTMGGACGYGNLYSQGYGTSTAALSTALFNNGLSCGAC
FQIRCNNDPKWCHSSTITVTATNFCPPNYALANDDGGWCNPPLQHFDLAQPAFLKIAQYRAGIVPVLFRRVPCVKKGGI
RFTINGHSYFNLVLITNVAGAGDVHAVSVKGSRTGWQTMSRNWGQDWQSNSYLNGQSLSFRVTASDGTTVTSYNIVPAN
WQFGQTFEGGQF
>PtEXPA15
MAIRATICVASLVLFLTLVEARIPGVYTGGAWQSAHATFYGGSDASGTMGGACGYGNLYSQGYGVNTAALSTALFNNGL

SCGACFEIKCGNDPQWCHSGSPSILITATNFCPPNYALPSDNGGWCNPPRPHFDLAMPVFLKIAEYRAGIVPVSYRRVP
CRKRGGIRFQINGHRYFNLVLITNVAGAGDIVKVSVKGSKTGWMSMSRNWGQNWQSNSVLVGQSLSFRVTGSDRRTSTS
WNVVPSNWQFSQTFVGKNFRV
>PtEXPA16
MAHHRLGLLCIVFLTSVFVLAHARIPGVFTGGPWESAHATFYGGSDASGTMGGACGYGNLYSQGYGVNTAALSTALFNN
GLSCGACFEIKCADDPRWCNPGNPSILVTATNFCPPNFAQPSDNGGWCNPPRPHFDLAMPMFLKIAQYRAGIVPVAHRR
VPCRKQGGIRFTINGFRYFNLVLVTNVAGAGDIVNVWIKGSETDWMSMSRNWGQNWQSNAVLVGQSLSFRVRGSDGRTS
TSKNVASPNWQFGQTFTGKNFRI
>PtEXPA17
MGKLLLAFAIWLQFCNYFAINVNAFAPSGWTKGHATFYGGSDASGTMGGACGYGNLYSTGYGTRTAALSTALFNDGASC
GECYRIMCDFQTDSRWCIKGRSVTITATNFCPPNFALPNNNGGWCNPPLQHFDMAQPAWEKIGIYRGGIVPVLFQRVPC
KKHGGVRFTINGRNYFELVLITNVAGAGSIQSVSIKGSKTGWLAMSRNWGVNWQSNAYLNGQSLSFKVTTTDGQTRFFT
DIVPANWGFGQTFQSSVQF
>PtEXPA18
MGKFLLTFAILLQFCSYLAINVNAFAPSGWRKGHATFYGGSDASGTMGGACGYGNLYSTGYGTRTAALSTALFNDGASC
GECYRIMCDFQTDPRWCIKGKSVTITATNFCPPNYALPNNNGGWCNPPLQHFDMAQPAWEKIGIYRGGIVPVLFQRVPC
KKHGGVRFTINGRNYFELVLISNVAGAGSIQSVSIKGSKTGWMAMSRNWGANWQSNAYLNGQSLSFKITNTDGQTRFFT
DIAPANWGFGQTFSSSVQF
>PtEXPA19
MASLSVLSCAVFLFLGIYCTQFHSASAAWQQAHATFYGGSDASGTMGGACGYGNLYTDGYGIKTAALSTALFNDGKSCG
GCYQIICDSTKVPQWCLRGKYITITATNFCPPNYNLPNDNGGWCNPPRPHFDMSQPAFQTIAKYRAGIVPILYRKVGCD
RSGGIRFTINGKNYFELVLISNVGGAGDISRVWIKGSKSNQWEAMSRNWGSNWQSLSYLNGQSLSFKIQTSNGQTRTAL
NVAPSNWVFGQSFKSNVQF
>PtEXPA20
MGPFVTLSVLVCQFLFFVCLLSLKGINGGWLNAHATFYGANQSPATLGGACGYDNTFHAGFGVNTAAVSGALFRGGEAC
GACYQLMCNYRADPKWCLRRAVITVTATNFCPPNNNGGWCDPPRQHFDMSMPAFFRIARQGNEGIVPVLYRRVSCTRRG
GVRFTLRGQSNFNLVMISNVGGSGDIKAAWVRGSRARSWVPLHRNWGANWQSSFDLRNQKLSFKLTLVDGKTLEFFNVV
PSTWMFGQTFSSRSQ
>PtEXPA21
MGALKAILLYLIIHQTWKITADDEEWKSATATYVKETSGSIIVEGACGYGDLHRATYGKYSAGLSSMLFNRGSTCGACF
EVRCVDHILWCLQGSPSVILTATDFCPPNYGLSSDYGGWCNFPKEHFDMSEAAFAEIAEKKADVVPVQHRRVKCDRRGG
LRFTMSGNFHFYQVLISNVGLDGEVIAVKVKGSRTAWIPMARNWGQNWQSNINLIGQPLSFEVTTSSRRTLTSYNVAPA
NWQFGQIFEGKQF
>PtEXPA22
MGALKAMLLYLIIHQTWKITADDEEWKSATATYVKETNGSIVVEGACGYADLHKATYGKYSAGLSSMLFNRGSTCGACF
EVRCVDHILWCLQGSPSVILTATDFCPPNYGLSADYGGWCNFPKEHFDMSEAAFTEIAEKKADIVPVQHRRVKCERRGG
LRFTVSGNFHFYQVLISNVGLDGEVIAVKVKGSKTAWIPMARNWGQNWQSNVNLIGQPLSFEVTTSSRKTLTSYNVAPA
NWQFGQTFEGKQF
>PtEXPA23
MPPPLLQGPIHTLLKPLLLLLLLLLTLPPTTTSHTSSTTTPPPLSTYLSQWQPARATYYAASDPRDTVGGACGYGDLVKA
GYGMATVALSESMFERGQICGACFEIKCVDDLRWCIPGTSIIVSVTNFCAPNYGFPSDAGGKCNTPNKHFVLPIESFEK
IAIWKAANMPVQYRRIKCRKEGGIRFTISGSGIFLSVLISNVAGAGDVTSVRIKGSRTGWLDMGRNWGQNWHVNANLQN
QALSFEVTSSHKMTVLSYTVAPKDWRFGQTFEGKQFET
>PtEXPA24
MASFFHSWSFSLFLIVLTSAMFIGKSMAVGYGKARVPTVFRPSQWSLAHATFYGDETARETMGGACGYGNLFQTGYGTN
TAALSTTLFNNGYACGTCYQIKCTNAPACYGVITTVTATNICPPNWSQDSNNGGWCNPPRVHFDMSKPAFMKIAQWKAG
IVPVMYRRVPCARTGGLRFKLQGNGYWLLVYVMNVGGGGDIASMWVKGSKTGWISMSHNWGASYQAFASLRGQALSFKI
TSYTTKQTVIALNVAPSTWGAGMTYKSTVNFS
>PtEXPA25
MASPLKSILATLLLLAIALPDCQAKSKPSLLGKQAIHHAIGRQANHIELPKRHKPKFRPGPWKKAHATFYEGGSGTFGG
ACNYKDVAGEGYGMNTVALSSVLFRNGQACGACFEIKCADSPQWCKLGQPSLFVTATDHCPPNPSLPNDNGGWCNVPRE
HFDIAKPVFSQLAGYKAGIIPIQYRRLPCKKQGGIRFTILGNPWFYQVIVWNVGGAGDVVSVQVKGDDKLKWTQMERDW
GATWKTSAILHGESLSFRVRASDERYSTSWHVTPKNWQFGQTYEGKNFK
>PtEXPA26

MASPLKPMLATLLLLAIAFPNCQAKSKPSLSGKQAIHHAIGRLAHDIEIPKRNKPKFQPSPWKEAHATFYEGGSGTFGG
ACNYKDVAGQGYGMNTAALSSVLFKNGQACGACFEIKCADNPQWCKLGQPSLIVTATDHCPPNPSLPNDNGGWCNVPRE
HFDVAKPVFSQLAEYEAGIIPIQYRRVPCQKQGGIRFTILGNPWFYQVIVWNVGGAGDVVGVQVKGDDKLKWTQMERDW
GTTWKTSAILLGESLSFRVSASDDRDSTSWHVTPKNWQFGQTYEGKNFK
>PtEXPA27
MASFKNSFACLVLMALLGVARSDQISFGEKIDSSWYDAHATFYGDIKGGQTMMGACGYGDLFKQGYGLQTAALSTALFN
NGQTCGACFELKCVNDPQWCKNHAGTIKITATNLCPPNYGASNAWCNPPQQHFDLSMPMFLTIAEYRAGIVPVKYRRIL
CSKEGGVKFDIHGNPYWMLVLVRNVAGAGEVINVRIKGSQTRWIQMSRNWGQNWQTGTDLVGQSLSFLVTTSDYKRLYF
NDVAGADWSFGQAYDGKINF
>PtEXPB1
MAIISFQNPFHLPIILAFFLVNSCHCFHPKLFNVSKIQSNSDWSPAGATWYGSPTGAGSDGGACGYGNAVEQAPFSSFI
SAGGDSIYKSGQGCGACYQVKCTSSSNAACSGNPVTVVITDQCPGSPCAQESFHFDLSGTAFGAMAISGKEDQLRNAGV
LQIQHQRVPCNWPGKTVTFHVDSGSNPYYFATVVEYEDGDGELKSVELKQALDSDSWVPMQKSWGAVWKLDAGSLLRAP
LSIKLTSLESGKTIVASGVIPAGWAPGQTYRSLVNFN
>PtEXPB2
MQLLGLYVVVFLKCFLGVFGQQVHPQHNVADLHWKPATATWYGSPDGDGSDGGACGYGSLVDVKPFRARVGAVSPVLFK
NGEGCGACYKVRCLDKSICSRRAVTIIVTDECPGGYCSNGNTHFDLSGAAFGRMAISGENGQLRNRGEIPVIYRRTPCK
YPGKNIAFHVNEGSTDYWLSILVEFEDGDGDVGSMHIREAGGTEWLEMNHVWGATWCIIRGPLKGPFSVKLTTLTTGRT
LSAREVIPRNWAPKATYTSRLNFFH
>PtEXPB3
MQLLGLHVVMLLKCFLVVFGQQYHTHHDVADLHWKPATATWYGSPDGDGSNGGACGYGSLVDVKPLRARVGAVSPILFK
NGEGCGACYKVRCLDKSICSERAVTIIVTDECPGGYCSNGNTHFDLSGAAFGHMAISGENGQLRNRGEIPVIYRRTPCK
YPGKNIAFHVNEGSTDYWLSLLVEFEDGDGDVGSMHIREAGGTEWLEMNHLWGANWCVIRGPLKGPLSVKLTTLSTGRT
LSARDVIPRNWVPKATYTSRLNFFH
>PtEXLA1
MAIFICFLFLAISYATACDRCVHQSKVAYFSRDSALSSGACGYGSMATGFNSGHLAAAVSSLYKDGSGCGACFQIRCKD
TTLCSREGTRVIVTDLNRNNQTDFVLSSRAFMAMANKDKGRNILKQGIVEVEYKRVPCEYKNQNLAVRVEESSKNSNYL
AIKLLYQGGQTEVVAIDFAKVGSSNWGFMSRNHGAVWDSDRVPSGALQFRFVITAGFDGKWIWARNVLPEDWKPGMTYD
SGVQITDIAQEGCSPCDDGIWK
>PtEXLA2
MAVFLCFLFLIISSATGCDRCVHQSKVAYFSKASALSTGACGYGSMAIGFNSGHLAAAVSSLYKDGAGCGACFQIRCKN
TALCSSRGTTVIVTDLNNNNQTDFVLSSRAFMAMANKGMGQDVLKHGILDVEYKRVPCEYKNQNLAVRVEESSKKPNYL
AIKLLYQGGQTEVVAMDVAKVGSSNWGFMSRNHGAVWDTDRVPAGALQFRFVVTAGFDGKWIWAQKVLPEDWKPGMTYD
SGVQITDIAQEGCSPCGDGIWK
>PtEXLB1
MGFAFKYGYSLLCIMALLPAISYSQDYTFSRATYYGSPDCLGTPSGACGFGEYGRTVNDANVAGVSRLYKNGTGCGGCY
QVRCKAPNLCTDDGVNVVVTDYGEGDKTDFILSPRAYARMAHPNMAVELIAYGVVDVEFRRIPCRYSGYNLMFKVHEHS
RFPDYLAIILLYQAGQNEILAVELWQEDCKEWRGMRRAYGAVWDMPNPPKGAISLRFQVSGSAGVTYWAQAANVIPSDW
KAGIAYDSTVQLS
>PtEXLB2
MGFALKYGYSILCVVAVALLPAISHSQDYTCSRATYFGSPDCLGTPTGACGFGGYGRTVNDANVAGVSRLFKNGTGCGG
CYQVRCKASNLCSDDGVNVVVTDYGEGDKTDFILSTRAYARMARPNMALELFAYGVVDVEFRRIPCRYNGYNLMFKVHE
HSRFPEYLAIVLLYQAGQNEILAVELRQEDCKEWRGMRRAYGAVWDIPNPPKGAISLKLQVCGSAGVTWVQADNVLPSD
WKAGVAYDSAIQLP
>PtEXLB3
MVPTLQFLLFLSGTLLFMQTLAEAATCSDCFIHSRAAYYPNSYENGTDTGRCGYGSFGATLNGGDVSAVSDLYRDGVGC
GACYQVRCTDSHYCTDKGVTIVVTDHGSSHNTDFILSRRAFGRMAQTTDAAASLIALGVVDVEYKRVSCSYPNKNITIK
IDENSDHPSYMGFVVWYQQGRRDITAVHLCETQNFACKVLDRSYGAVWTTTSPPSGPLSLRMLFSEEDGDETWVVPVNN
IPNDWKAGQTYDTGVQVNS
>PtEXLB4
MAPSLRFLLFLSATLFFMQTLTEAATCSDCFIHSRAAYYPNSDENGTDTGRCGYGSFGATLNGGDVAAASDLYRDGVGC
GACYQVKCTNSYYCTDNGVTIVITDQGSSDNTDFILSRRAFGRMAQTTDAAASLLALGVVDIEYRRVSCSYPYKNITIK
IDENSNYPYYLGFVIWYQQGRSDITAVQLCETQNFVCKLLDRSYGSVWTTTSPPSGPLSLRMLFSDENGDETWVVPVNN
IPNDWKAGETYDTGVQVNL
