# Supplementary material for: Genome-Wide Identification of Expansin Gene Family and Their Response under Hormone Exposure in Ginkgo biloba L
Source: Int J Mol Sci. 2023 Mar 21;24(6):5901. doi: 10.3390/ijms24065901 (PMC10053239; doi:10.3390/ijms24065901)
Supplement: Supplementary file 1 [file ijms-24-05901-s001.zip › Table S1-S2.pdf]

Table S1. List of expansin (EXP) genes in *Ginkgo biloba*

Gene name	ID	Formula	Number of amino acids	Molecular Weight/kD	Theoretical pI	Instability index	Aliphatic index	Grand average of hydropathicity (GRAVY)	Predicted subcellular localization
GbEXPA1	evm.model.chr1.1594	$C_{1210}H_{1827}N_{339}O_{351}S_{16}$	247	27251.84	8.90	37.13	67.17	-0.253	chloroplast
GbEXPA2	evm.model.chr1.1892	$C_{1238}H_{1880}N_{336}O_{372}S_{18}$	262	27999.65	8.06	42.10	65.99	-0.069	extracellular
GbEXPA3	evm.model.chr1.2822	$C_{1216}H_{1813}N_{325}O_{360}S_{14}$	252	27193.57	7.55	33.32	66.59	-0.122	extracellular
GbEXPA4	evm.model.chr1.2823	$C_{1144}H_{1711}N_{319}O_{337}S_{13}$	233	25741.88	8.76	30.97	60.64	-0.404	nucleus
GbEXPA5	evm.model.chr1.2830	$C_{1380}H_{2089}N_{379}O_{392}S_{15}$	283	30741.97	9.03	36.48	74.77	-0.036	chloroplast
GbEXPA6	evm.model.chr1.2831	$C_{1225}H_{1822}N_{332}O_{358}S_{14}$	251	27376.79	8.60	31.56	65.70	-0.194	extracellular
GbEXPA7	evm.model.chr1.2832	$C_{1294}H_{1927}N_{353}O_{386}S_{15}$	267	29085.57	8.05	32.35	62.81	-0.263	nucleus
GbEXPA8	evm.model.chr1.2833	$C_{1207}H_{1793}N_{331}O_{356}S_{14}$	250	27085.36	8.41	35.46	64.40	-0.200	extracellular
GbEXPA9	evm.model.chr1.2834	$C_{1207}H_{1802}N_{330}O_{353}S_{16}$	250	27096.54	8.61	35.03	64.80	-0.177	extracellular
GbEXPA10	evm.model.chr1.2835	$C_{1750}H_{2653}N_{483}O_{503}S_{19}$	357	39115.39	8.87	37.34	71.88	-0.198	cytoplasm
GbEXPA11	evm.model.chr1.2836	$C_{1149}H_{1692}N_{314}O_{337}S_{15}$	238	25776.88	7.56	33.88	59.03	-0.215	chloroplast
GbEXPA12	evm.model.chr3.288	$C_{1127}H_{1704}N_{316}O_{332}S_{15}$	235	25472.74	9.19	33.21	62.34	-0.190	plasma membrane
GbEXPA13	evm.model.chr4.1947	$C_{1231}H_{1834}N_{328}O_{361}S_{13}$	253	27420.86	8.34	33.11	69.01	-0.081	extracellular
GbEXPA14	evm.model.chr4.2179	$C_{1255}H_{1857}N_{337}O_{373}S_{18}$	256	28210.66	5.50	35.68	62.46	-0.205	chloroplast
GbEXPA15	evm.model.chr7.379	$C_{1216}H_{1826}N_{332}O_{368}S_{12}$	257	27368.60	6.79	31.87	67.59	-0.167	chloroplast
GbEXPA16	evm.model.chr8.517	$C_{1349}H_{1998}N_{358}O_{405}S_{16}$	282	30223.82	4.61	32.38	70.60	-0.009	chloroplast
GbEXPA17	evm.model.chr8.518	$C_{1209}H_{1809}N_{339}O_{356}S_{14}$	254	27237.56	8.63	33.11	65.71	-0.166	extracellular
GbEXPA18	evm.model.chr9.1784	$C_{1241}H_{1868}N_{342}O_{375}S_{19}$	259	28187.69	5.92	44.21	64.05	-0.182	chloroplast
GbEXPA19	evm.model.chr9.473	$C_{1254}H_{1925}N_{365}O_{356}S_{18}$	260	28387.39	9.57	33.44	69.46	-0.070	nucleus
GbEXPA20	evm.model.chr10.1814	$C_{1286}H_{1959}N_{361}O_{346}S_{13}$	259	28429.69	9.98	38.13	76.45	-0.059	chloroplast

$GbEXPA_{21}$	evm.model.chr10.1818	$C_{1251}H_{1908}N_{346}O_{355}S_{17}$	253	28020.04	9.09	33.45	68.58	-0.251	chloroplast
$GbEXPA_{22}$	evm.model.chr11.4	$C_{1403}H_{2103}N_{379}O_{424}S_{19}$	292	31672.56	5.47	24.01	65.75	-0.139	chloroplast
$GbEXPA_{23}$	evm.model.chr11.464	$C_{1215}H_{1811}N_{341}O_{367}S_{18}$	255	27643.89	8.10	33.93	58.55	-0.250	plasma member
$GbEXPA_{24}$	evm.model.chr11.483	$C_{1106}H_{1644}N_{318}O_{330}S_{12}$	231	25059.87	8.63	34.00	60.39	-0.343	nucleus
$GbEXPA_{25}$	evm.model.chr11.484	$C_{1210}H_{1805}N_{331}O_{368}S_{16}$	253	27389.60	6.79	38.20	60.91	-0.231	chloroplast
$GbEXPA_{26}$	evm.model.chr11.485	$C_{1207}H_{1799}N_{331}O_{368}S_{16}$	253	27347.52	6.79	38.20	60.16	-0.241	Chloroplast
$GbEXPA_{27}$	evm.model.chr11.486	$C_{1204}H_{1792}N_{332}O_{369}S_{15}$	253	27302.38	7.53	36.02	58.62	-0.279	chloroplast
$GbEXPA_{28}$	evm.model.chr11.488	$C_{1208}H_{1802}N_{332}O_{368}S_{16}$	253	27376.56	7.53	37.74	59.37	-0.252	chloroplast
$GbEXPA_{29}$	evm.model.chr11.489	$C_{1181}H_{1767}N_{333}O_{360}S_{18}$	252	26967.12	8.03	35.17	58.93	-0.183	extracellular
$GbEXPA_{30}$	evm.model.chr11.490	$C_{1200}H_{1791}N_{333}O_{367}S_{18}$	254	27331.51	6.79	38.03	57.64	-0.218	extracellular
$GbEXPA_{31}$	evm.model.chr11.491	$C_{1104}H_{1645}N_{307}O_{336}S_{13}$	234	25010.84	8.11	25.34	59.66	-0.248	chloroplast
$GbEXPA_{32}$	evm.model.chr11.6	$C_{1837}H_{2819}N_{513}O_{522}S_{25}$	370	41244.22	9.36	37.86	65.62	-0.252	endoplasmic reticulum
$GbEXPB_{1}$	evm.model.chr1.947	$C_{1271}H_{1941}N_{339}O_{391}S_{13}$	272	28643.21	4.95	35.10	76.43	-0.048	plasma member
$GbEXPB_{2}$	evm.model.chr9.667	$C_{1363}H_{2088}N_{364}O_{398}S_{19}$	279	30550.91	5.70	32.25	75.88	-0.128	extracellular
$GbEXPB_{3}$	evm.model.chr9.669	$C_{1298}H_{1977}N_{347}O_{391}S_{16}$	269	29212.03	5.33	37.51	75.06	-0.131	extracellular
$GbEXPB_{4}$	evm.model.chr9.670	$C_{1347}H_{2065}N_{363}O_{394}S_{17}$	275	30193.43	6.08	38.23	75.89	-0.147	extracellular
$GbEXLA_{1}$	evm.model.chr2.1505	$C_{1344}H_{2054}N_{366}O_{386}S_{19}$	273	30124.45	8.61	37.26	75.02	-0.160	extracellular
$GbEXLA_{2}$	evm.model.chr2.1506	$C_{1315}H_{2031}N_{343}O_{385}S_{15}$	267	29286.56	8.59	34.82	77.83	-0.043	extracellular
$GbEXLA_{3}$	evm.model.chr7.777	$C_{1267}H_{1941}N_{335}O_{353}S_{14}$	254	27963.22	7.96	29.20	90.55	0.044	chloroplast
$GbEXLA_{4}$	evm.model.chr7.785	$C_{1326}H_{2062}N_{354}O_{405}S_{18}$	271	30020.17	5.59	35.87	78.67	-0.191	chloroplast
$GbEXLA_{5}$	evm.model.chr7.786	$C_{1323}H_{2056}N_{353}O_{404}S_{17}$	268	29389.94	8.75	39.40	86.90	-0.027	chloroplast
$GbEXLB_{1}$	evm.model.chr2.1504	$C_{1190}H_{1823}N_{319}O_{368}S_{20}$	250	27127.68	4.97	32.79	72.16	-0.081	extracellular
$GbEXLB_{2}$	evm.model.chr2.1507	$C_{1205}H_{1820}N_{310}O_{371}S_{18}$	249	27162.64	4.62	29.00	76.02	-0.030	chloroplast
$GbEXLB_{3}$	evm.model.chr2.1508	$C_{1203}H_{1815}N_{311}O_{372}S_{18}$	249	27163.59	4.62	28.40	74.46	-0.070	chloroplast
$GbEXLB_{4}$	evm.model.chr5.818	$C_{1844}H_{2849}N_{515}O_{540}S_{25}$	373	41674.53	7.87	43.77	76.33	-0.248	extracellular
$GbEXLB_{5}$	evm.model.chr5.819	$C_{1199}H_{1821}N_{323}O_{359}S_{19}$	245	27113.74	6.12	37.59	73.59	-0.134	chloroplast

Table S2. GbEXPs primer sequences.

Gene name	Forward primer (5' to 3')	Reverse primer (5' to 3')
GbEXPA31(ORF)	ATGGCCCAGTTTCTCCAGACA	TCAATTGCCAAGTTGGGCTCC
GbEXPA31	ATGGCCCAGTTTCTCCAGAC	CCGTGTTCGTGCCATATCCTTG
GbEXPA24	CTGCAACCAACTTCTGCCCG	GCACAATGCCTGCCCTGTAC
GbEXPA32	GGAAGCGATGCCTCAGACACTATG	GTGGGTCGTCGTAACACTTGATCTC
GbEXLA5	AGGACAAACAGACATAGTGGCAGTG	ACCACACCCATTTCCCATCATAACC
GbEXPA16	GTTGGAGGAGCAGGAGATGTCATTG	GAGAGGCTTTGGGCAGTCAAGTG
GbEXPB4	CGGATGAATGCCCAGGAGGATATTG	GTCGGTAGAGCCTTCGTTCACATG
GbEXLB5	CTATGCACGGGCGAGGGTAA	TCCCGAGGTCACTAGGTCGT
GbEXLB1	ATGTGGTGCTTGCTATCAGGTGAAG	ACTGGATTGCTACAACTCCCATTGC
GAPDH	CTGCCAAGGCTGTAGGTAAGG	TCAGATTCCTCCTTGATGGCG
GbEXPA31-EGFP	ttggagaggacacgctcgag<br>ATGGCCCAGTTTCTCCAGAC	cccttgctcaccatgaattc<br>ATTGCCAAGTTGGGCTCC
