# Supplementary material for: Genome-Wide Identification of Expansin Gene Family and Their Response under Hormone Exposure in Ginkgo biloba L
Source: Int J Mol Sci. 2023 Mar 21;24(6):5901. doi: 10.3390/ijms24065901 (PMC10053239; doi:10.3390/ijms24065901)
Supplement: Supplementary file 1 [file ijms-24-05901-s001.zip › Table S3.GbEXPs cis-elements.pdf]

GbEXPA1	Box 4	ATTAAT	740	6 +
GbEXPA1	ARE	AAACCA	150	6 +
GbEXPA1	ARE	AAACCA	652	6 -
GbEXPA1	TCT-motif	TCTTAC	1054	6 -
GbEXPA1	CGTCA-motif	CGTCA	1386	5 +
GbEXPA1	GCN4_motif	TGAGTCA	1330	7 +
GbEXPA1	P-box	CCTTTTG	1678	7 +
GbEXPA1	P-box	CCTTTTG	1832	7 +
GbEXPA1	GT1-motif	GGTTAA	123	6 -
GbEXPA1	GT1-motif	GGTTAA	991	6 +
GbEXPA1	GT1-motif	GGTTAA	1291	6 +
GbEXPA1	AuxRR-core	GGTCCAT	224	7 +
GbEXPA1	ABRE	ACGTG	806	5 -
GbEXPA1	G-box	TACGTG	806	6 -
GbEXPA1	GATA-motif	AAGGATAAGC	1500	9 +
GbEXPA1	MRE	AACCTAA	672	7 +
GbEXPA1	CAAT-box	CAAAT	1	5 +
GbEXPA1	TC-rich repeats	GTTTTCTTAC	1187	9 +
GbEXPA1	TGACG-motif	TGACG	1386	5 -
GbEXPA2	ACE	GACACGTATG	551	9 +
GbEXPA2	ACE	GACACGTATG	1351	9 +
GbEXPA2	ABRE	ACGTG	1353	5 -
GbEXPA2	ABRE	CACGTG	1426	6 -
GbEXPA2	ABRE	ACGTG	1427	5 +
GbEXPA2	ABRE	ACGTG	1691	5 +
GbEXPA2	P-box	CCTTTTG	39	7 -
GbEXPA2	ATCT-motif	AATCTAATCC	710	9 +
GbEXPA2	G-Box	CACGTG	1426	6 -
GbEXPA2	G-Box	CACGTT	1690	6 -
GbEXPA2	CGTCA-motif	CGTCA	577	5 -
GbEXPA2	TCT-motif	TCTTAC	280	6 -
GbEXPA2	TCT-motif	TCTTAC	588	6 -
GbEXPA2	RY-element	CATGCATG	744	8 +
GbEXPA2	Box 4	ATTAAT	250	6 +
GbEXPA2	Box 4	ATTAAT	1990	6 -
GbEXPA2	HD-Zip 1	CAAT(A/T)ATT(	1540	8.5 -
GbEXPA2	Gap-box	CAAATGAA(A/	1009	9.5 +
GbEXPA2	Box II	TGGTAATAA	185	9 -
GbEXPA2	TGACG-motif	TGACG	577	5 +
GbEXPA2	I-box	cCATATCCAAT	1947	10 -
GbEXPA2	TC-rich repeats	GTTTTCTTAC	280	9 -
GbEXPA2	TC-rich repeats	GTTTTCTTAC	424	9 -
GbEXPA2	AE-box	AGAAACAA	1227	8 +
GbEXPA2	A-box	CCGTCC	1722	6 -
GbEXPA2	MRE	AACCTAA	313	7 -
GbEXPA2	MRE	AACCTAA	457	7 -

GbEXPA2	G-box	TACGTG	1353	6 -
GbEXPA2	G-box	GCCACGTGGA	1424	9 -
GbEXPA2	G-box	CACGTG	1426	6 -
GbEXPA2	LTR	CCGAAA	1395	6 +
GbEXPA3	GARE-motif	TCTGTTG	1803	7 +
GbEXPA3	AT-rich element	ATAGAAATCA	1070	10 +
GbEXPA3	G-box	TAAACGTG	1015	8 +
GbEXPA3	G-box	CACGAC	1052	6 -
GbEXPA3	G-box	CACGTC	1181	6 +
GbEXPA3	TC-rich repeats	ATTCTCTAAC	1965	9 +
GbEXPA3	Box 4	ATTAAT	925	6 +
GbEXPA3	Box 4	ATTAAT	1007	6 -
GbEXPA3	Box 4	ATTAAT	1252	6 -
GbEXPA3	Box 4	ATTAAT	1585	6 -
GbEXPA3	G-Box	CACGTT	639	6 -
GbEXPA3	G-Box	CACGTT	1017	6 -
GbEXPA3	TCA-element	TCAGAAGAGG	1095	10 -
GbEXPA3	GCN4_motif	TGAGTCA	220	7 +
GbEXPA3	ABRE	ACGTG	640	5 +
GbEXPA3	ABRE	ACGTG	1018	5 +
GbEXPA3	ABRE	ACGTG	1181	5 -
GbEXPA3	ABRE	GCAACGTGTC	1352	9 -
GbEXPA3	AuxRR-core	GGTCCAT	7	7 +
GbEXPA3	AuxRR-core	GGTCCAT	12	7 -
GbEXPA4	Box 4	ATTAAT	527	6 +
GbEXPA4	Box 4	ATTAAT	1053	6 -
GbEXPA4	Box 4	ATTAAT	1298	6 -
GbEXPA4	Box 4	ATTAAT	1631	6 -
GbEXPA4	G-Box	CACGTT	802	6 -
GbEXPA4	G-Box	CACGTT	1063	6 -
GbEXPA4	GCN4_motif	TGAGTCA	447	7 +
GbEXPA4	TCA-element	TCAGAAGAGG	1141	10 -
GbEXPA4	AuxRR-core	GGTCCAT	234	7 +
GbEXPA4	AuxRR-core	GGTCCAT	239	7 -
GbEXPA4	ABRE	ACGTG	803	5 +
GbEXPA4	ABRE	ACGTG	1064	5 +
GbEXPA4	ABRE	ACGTG	1227	5 -
GbEXPA4	ABRE	GCAACGTGTC	1398	9 -
GbEXPA4	AT-rich element	ATAGAAATCA	1116	10 +
GbEXPA4	GARE-motif	TCTGTTG	1849	7 +
GbEXPA4	G-box	TAAACGTG	1061	8 +
GbEXPA4	G-box	CACGAC	1098	6 -
GbEXPA4	G-box	CACGTC	1227	6 +
GbEXPA4	A-box	CCGTCC	1280	6 -
GbEXPA5	AE-box	AGAAACAA	1412	8 -
GbEXPA5	A-box	CCGTCC	1730	6 +

GbEXPA5	TGACG-motif	TGACG	523	5 +
GbEXPA5	TGACG-motif	TGACG	1660	5 +
GbEXPA5	TGACG-motif	TGACG	1720	5 +
GbEXPA5	G-box	TACGTG	129	6 -
GbEXPA5	chs-CMA1a	TTACTTAA	1535	8 +
GbEXPA5	MRE	AACCTAA	881	7 +
GbEXPA5	TCA-element	CCATCTTTTT	168	9 -
GbEXPA5	ABRE	ACGTG	129	5 -
GbEXPA5	AuxRR-core	GGTCCAT	46	7 +
GbEXPA5	TCT-motif	TCTTAC	39	6 -
GbEXPA5	AT1-motif	AATTATTTTTT	672	13 -
GbEXPA5	ARE	AAACCA	973	6 -
GbEXPA5	ARE	AAACCA	1682	6 -
GbEXPA5	ARE	AAACCA	1994	6 -
GbEXPA5	Box 4	ATTAAT	455	6 +
GbEXPA5	CGTCA-motif	CGTCA	523	5 -
GbEXPA5	CGTCA-motif	CGTCA	1660	5 -
GbEXPA5	CGTCA-motif	CGTCA	1720	5 -
GbEXPA5	MBS	CAACTG	1932	6 -
GbEXPA6	AT-rich element	ATAGAAATCA	1261	10 -
GbEXPA6	G-box	CACGTC	471	6 -
GbEXPA6	TGACG-motif	TGACG	641	5 +
GbEXPA6	TGACG-motif	TGACG	832	5 +
GbEXPA6	TGACG-motif	TGACG	1755	5 -
GbEXPA6	TCCC-motif	TCTCCCT	1862	7 +
GbEXPA6	MBS	CAACTG	75	6 -
GbEXPA6	MBS	CAACTG	281	6 +
GbEXPA6	MBS	CAACTG	945	6 -
GbEXPA6	G-Box	CACGTT	1814	6 -
GbEXPA6	CGTCA-motif	CGTCA	641	5 -
GbEXPA6	CGTCA-motif	CGTCA	832	5 -
GbEXPA6	CGTCA-motif	CGTCA	1755	5 +
GbEXPA6	Box 4	ATTAAT	424	6 +
GbEXPA6	Box 4	ATTAAT	548	6 +
GbEXPA6	Box 4	ATTAAT	1158	6 -
GbEXPA6	Box 4	ATTAAT	1220	6 -
GbEXPA6	HD-Zip 1	CAAT(A/T)ATT	1083	8.5 -
GbEXPA6	ABRE	ACGTG	472	5 +
GbEXPA6	ABRE	ACGTG	1815	5 +
GbEXPA6	P-box	CCTTTTG	17	7 -
GbEXPA7	Box 4	ATTAAT	528	6 +
GbEXPA7	Box 4	ATTAAT	1054	6 -
GbEXPA7	Box 4	ATTAAT	1298	6 -
GbEXPA7	Box 4	ATTAAT	1631	6 -
GbEXPA7	G-Box	CACGTT	803	6 -
GbEXPA7	G-Box	CACGTT	1064	6 -

GbEXPA7	MBS	CAACTG	1680	6 -
GbEXPA7	GCN4_motif	TGAGTCA	448	7 +
GbEXPA7	TCA-element	TCAGAAGAGG	1142	10 -
GbEXPA7	AuxRR-core	GGTCCAT	235	7 +
GbEXPA7	AuxRR-core	GGTCCAT	240	7 -
GbEXPA7	ABRE	ACGTG	804	5 +
GbEXPA7	ABRE	ACGTG	1065	5 +
GbEXPA7	ABRE	ACGTG	1228	5 -
GbEXPA7	ABRE	GCAACGTGTC	1398	9 -
GbEXPA7	G-box	TAAACGTG	1062	8 +
GbEXPA7	G-box	CACGAC	1099	6 -
GbEXPA7	G-box	CACGTC	1228	6 +
GbEXPA7	GARE-motif	TCTGTTG	1849	7 +
GbEXPA8	TGACG-motif	TGACG	1510	5 +
GbEXPA8	G-box	CACGTG	897	6 +
GbEXPA8	GATA-motif	AAGGATAAGC	1245	9 +
GbEXPA8	GATA-motif	AAGGATAAGC	1422	9 -
GbEXPA8	GARE-motif	TCTGTTG	1833	7 +
GbEXPA8	LTR	CCGAAA	1841	6 -
GbEXPA8	GT1-motif	GTGTGTGAA	584	9 -
GbEXPA8	GT1-motif	GGTTAA	1414	6 -
GbEXPA8	ABRE	CGTACGTGCA	1	9 -
GbEXPA8	ABRE	CACGTG	897	6 +
GbEXPA8	ABRE	ACGTG	898	5 +
GbEXPA8	CGTCA-motif	CGTCA	1510	5 -
GbEXPA8	G-Box	CACGTG	897	6 +
GbEXPA8	MBS	CAACTG	741	6 +
GbEXPA8	MBS	CAACTG	1692	6 -
GbEXPA8	O2-site	GATGA(C/T)(A/	1774	8.5 -
GbEXPA8	Gap-box	CAAATGAA(A/	1878	9.5 -
GbEXPA8	Box 4	ATTAAT	85	6 +
GbEXPA8	Box 4	ATTAAT	239	6 +
GbEXPA8	Box 4	ATTAAT	658	6 +
GbEXPA8	Box 4	ATTAAT	1321	6 -
GbEXPA8	Box 4	ATTAAT	1637	6 -
GbEXPA9	P-box	TTCCAACAAA	210	13 +
GbEXPA9	AACA_motif	TAACAAACTC	213	11 +
GbEXPA9	GCN4_motif	TGAGTCA	1192	7 -
GbEXPA9	AuxRR-core	GGTCCAT	396	7 -
GbEXPA9	AuxRR-core	GGTCCAT	1623	7 +
GbEXPA9	GT1-motif	GGTTAAT	304	7 -
GbEXPA9	GT1-motif	GGTTAA	305	6 -
GbEXPA9	ABRE	ACGTG	421	5 +
GbEXPA9	Box 4	ATTAAT	407	6 +
GbEXPA9	CAT-box	GCCACT	817	6 +
GbEXPA9	CGTCA-motif	CGTCA	419	5 -

GbEXPA9	CGTCA-motif	CGTCA	1231	5 -
GbEXPA9	CGTCA-motif	CGTCA	1255	5 +
GbEXPA9	TGACG-motif	TGACG	419	5 +
GbEXPA9	TGACG-motif	TGACG	1231	5 +
GbEXPA9	TGACG-motif	TGACG	1255	5 -
GbEXPA9	TC-rich repeats	ATTCTCTAAC	688	9 -
GbEXPA9	TGA-element	AACGAC	1238	6 +
GbEXPA9	G-box	CACGTC	420	6 -
GbEXPA9	G-box	CACGAC	660	6 +
GbEXPA9	G-box	CACGAC	1166	6 -
GbEXPA10	TGACG-motif	TGACG	284	5 +
GbEXPA10	TGACG-motif	TGACG	1143	5 -
GbEXPA10	TGACG-motif	TGACG	1630	5 +
GbEXPA10	TGA-element	AACGAC	1242	6 -
GbEXPA10	GARE-motif	TCTGTTG	1162	7 +
GbEXPA10	G-box	TACGTG	1708	6 +
GbEXPA10	G-box	TACGTG	1755	6 +
GbEXPA10	P-box	CCTTTTG	392	7 +
GbEXPA10	ACE	GACACGTATG	1706	9 -
GbEXPA10	ABRE	ACGTG	904	5 +
GbEXPA10	ABRE	ACGTG	1045	5 -
GbEXPA10	ABRE	GACACGTACG	1705	10 -
GbEXPA10	ABRE	TACGTGTC	1708	8 +
GbEXPA10	ABRE	ACGTG	1709	5 +
GbEXPA10	ABRE	ACGTG	1756	5 +
GbEXPA10	ABRE	ACGTG	1816	5 -
GbEXPA10	Box 4	ATTAAT	323	6 +
GbEXPA10	Box 4	ATTAAT	561	6 +
GbEXPA10	Box 4	ATTAAT	1482	6 -
GbEXPA10	Box 4	ATTAAT	1740	6 -
GbEXPA10	CAT-box	GCCACT	623	6 -
GbEXPA10	TCT-motif	TCTTAC	158	6 +
GbEXPA10	TCT-motif	TCTTAC	495	6 +
GbEXPA10	ARE	AAACCA	1167	6 -
GbEXPA10	O2-site	GATGATGTGG	1468	9 -
GbEXPA10	ATCT-motif	AATCTAATCC	1530	9 +
GbEXPA10	ATCT-motif	AATCTAATCC	1593	9 -
GbEXPA10	CGTCA-motif	CGTCA	284	5 -
GbEXPA10	CGTCA-motif	CGTCA	1143	5 +
GbEXPA10	CGTCA-motif	CGTCA	1630	5 -
GbEXPA10	G-Box	CACGTT	903	6 -
GbEXPA10	G-Box	CACGTT	1045	6 +
GbEXPA10	G-Box	CACGTT	1816	6 +
GbEXPA11	G-box	TACGTG	331	6 -
GbEXPA11	G-box	CACGTG	497	6 +
GbEXPA11	G-box	TAAACGTG	667	8 +

GbEXPA11	G-box	CACGTC	690	6 -
GbEXPA11	I-box	GTATAAGGCC	334	9 +
GbEXPA11	I-box	TGATAATGT	1917	9 +
GbEXPA11	TGACG-motif	TGACG	394	5 -
GbEXPA11	TGACG-motif	TGACG	559	5 +
GbEXPA11	TGACG-motif	TGACG	1288	5 +
GbEXPA11	Box 4	ATTAAT	1099	6 -
GbEXPA11	Box 4	ATTAAT	1103	6 -
GbEXPA11	ARE	AAACCA	183	6 -
GbEXPA11	ARE	AAACCA	1122	6 +
GbEXPA11	ARE	AAACCA	1834	6 -
GbEXPA11	CGTCA-motif	CGTCA	394	5 +
GbEXPA11	CGTCA-motif	CGTCA	559	5 -
GbEXPA11	CGTCA-motif	CGTCA	1288	5 -
GbEXPA11	G-Box	CACGTG	497	6 +
GbEXPA11	G-Box	CACGTT	669	6 -
GbEXPA11	GT1-motif	GGTTAA	137	6 -
GbEXPA11	ABRE	ACGTG	331	5 -
GbEXPA11	ABRE	GACACGTGGC	495	9 +
GbEXPA11	ABRE	CACGTG	497	6 +
GbEXPA11	ABRE	ACGTG	498	5 +
GbEXPA11	ABRE	ACGTG	670	5 +
GbEXPA11	ABRE	ACGTG	691	5 +
GbEXPB1	O2-site	GTTGACGTGA	57	9 +
GbEXPB1	O2-site	GATGACATGG	351	9 +
GbEXPB1	O2-site	GATGACATGG	386	9 -
GbEXPB1	CAT-box	GCCACT	865	6 +
GbEXPB1	RY-element	CATGCATG	1455	8 -
GbEXPB1	Box 4	ATTAAT	28	6 +
GbEXPB1	Box 4	ATTAAT	168	6 +
GbEXPB1	Box 4	ATTAAT	735	6 +
GbEXPB1	G-Box	CACGTT	1289	6 -
GbEXPB1	G-Box	CACGTG	1757	6 -
GbEXPB1	CGTCA-motif	CGTCA	1436	5 -
GbEXPB1	CGTCA-motif	CGTCA	1698	5 -
GbEXPB1	CGTCA-motif	CGTCA	1754	5 +
GbEXPB1	MBS	CAACTG	1432	6 -
GbEXPB1	MBS	CAACTG	1825	6 -
GbEXPB1	MBS	CAACTG	1832	6 -
GbEXPB1	MBS	CAACTG	1914	6 +
GbEXPB1	ABRE	ACGTG	1290	5 +
GbEXPB1	ABRE	CACGTG	1757	6 -
GbEXPB1	ABRE	ACGTG	1758	5 +
GbEXPB1	GT1-motif	GGTTAA	49	6 +
GbEXPB1	GT1-motif	GGTTAA	85	6 -
GbEXPB1	GT1-motif	GGTTAA	109	6 +

GbEXPB1	GT1-motif	GGTTAA	198	6 -
GbEXPB1	LTR	CCGAAA	1841	6 -
GbEXPB1	GC-motif	CCCCCG	1529	6 +
GbEXPB1	GC-motif	CCCCCG	1893	6 -
GbEXPB1	G-box	GCCACGTGGA	1755	9 +
GbEXPB1	G-box	CACGTG	1757	6 -
GbEXPB1	TGA-element	AACGAC	1507	6 -
GbEXPB1	TGA-element	AACGAC	1547	6 +
GbEXPB1	GATA-motif	AAGGATAAGC	1015	9 +
GbEXPB1	A-box	CCGTCC	1402	6 +
GbEXPB1	I-box	gGATAAGGTG	1651	9 -
GbEXPB1	TC-rich repeats	ATTCTCTAAC	773	9 +
GbEXPB1	TC-rich repeats	ATTCTCTAAC	969	9 -
GbEXPB1	TGACG-motif	TGACG	1436	5 +
GbEXPB1	TGACG-motif	TGACG	1698	5 +
GbEXPB1	TGACG-motif	TGACG	1754	5 -
GbEXPA20	ATC-motif	AGCTATCCA	761	9 +
GbEXPA20	CAG-motif	GAAAGGCAGA	170	10 +
GbEXPA20	chs-CMA1a	TTACTTAA	819	8 +
GbEXPA20	G-box	CACGTC	774	6 +
GbEXPA20	G-box	CACGTG	1835	6 -
GbEXPA20	GATA-motif	AAGGATAAGC	1673	9 +
GbEXPA20	TGA-element	AACGAC	777	6 -
GbEXPA20	TCA-element	CCATCTTTTT	1	9 +
GbEXPA20	P-box	CCTTTTG	1361	7 -
GbEXPA20	GT1-motif	GGTTAA	1876	6 +
GbEXPA20	ABRE	ACGTG	774	5 -
GbEXPA20	ABRE	CACGTG	1835	6 -
GbEXPA20	ABRE	ACGTG	1836	5 +
GbEXPA20	O2-site	GATGATGTGG	863	9 -
GbEXPA20	Box 4	ATTAAT	941	6 +
GbEXPA20	Box 4	ATTAAT	1503	6 -
GbEXPA20	CAT-box	GCCACT	1223	6 +
GbEXPA20	CAT-box	GCCACT	1657	6 -
GbEXPA20	TCT-motif	TCTTAC	981	6 +
GbEXPA20	G-Box	CACGTG	1835	6 -
GbEXPA21	TCCC-motif	TCTCCCT	330	7 +
GbEXPA21	TGACG-motif	TGACG	1487	5 +
GbEXPA21	TC-rich repeats	ATTCTCTAAC	1193	9 -
GbEXPA21	LTR	CCGAAA	885	6 +
GbEXPA21	LTR	CCGAAA	1500	6 -
GbEXPA21	TGA-element	AACGAC	1982	6 -
GbEXPA21	G-box	CACGAC	1391	6 -
GbEXPA21	P-box	CCTTTTG	201	7 +
GbEXPA21	AuxRR-core	GGTCCAT	1744	7 -
GbEXPA21	ABRE	ACGTG	1715	5 +

GbEXPA21	Box 4	ATTAAT	126	6	+
GbEXPA21	CAT-box	GCCACT	1413	6	+
GbEXPA21	CGTCA-motif	CGTCA	1487	5	-
GbEXPA21	G-Box	CACGTT	1714	6	-
GbEXPA22	G-box	TACGTG	1149	6	+
GbEXPA22	TGACG-motif	TGACG	1260	5	+
GbEXPA22	TC-rich repeats	ATTCTCTAAC	998	9	+
GbEXPA22	I-box	cCATATCCAAT	97	10	+
GbEXPA22	CGTCA-motif	CGTCA	1260	5	-
GbEXPA22	Box 4	ATTAAT	2	6	+
GbEXPA22	Box 4	ATTAAT	342	6	+
GbEXPA22	Box 4	ATTAAT	490	6	+
GbEXPA22	Box 4	ATTAAT	550	6	+
GbEXPA22	Box 4	ATTAAT	953	6	+
GbEXPA22	CAT-box	GCCACT	779	6	-
GbEXPA22	CAT-box	GCCACT	1529	6	+
GbEXPA22	O2-site	GATGATGTGG	1446	9	-
GbEXPA22	GT1-motif	GGTTAAT	939	7	-
GbEXPA22	GT1-motif	GGTTAA	940	6	-
GbEXPA22	ABRE	GACACGTGGC	247	9	-
GbEXPA22	ABRE	ACGTG	1150	5	+
GbEXPA22	GCN4_motif	TGAGTCA	163	7	-
GbEXPA23	CGTCA-motif	CGTCA	887	5	+
GbEXPA23	CGTCA-motif	CGTCA	1227	5	+
GbEXPA23	G-Box	CACGTG	876	6	+
GbEXPA23	G-Box	CACGTT	1688	6	-
GbEXPA23	Box 4	ATTAAT	856	6	+
GbEXPA23	Box 4	ATTAAT	1595	6	-
GbEXPA23	CAT-box	GCCACT	839	6	+
GbEXPA23	GT1-motif	GGTTAA	1684	6	+
GbEXPA23	ABRE	CACGTG	876	6	+
GbEXPA23	ABRE	ACGTG	877	5	+
GbEXPA23	ABRE	ACGTG	1689	5	+
GbEXPA23	Sp1	GGGCGG	517	6	-
GbEXPA23	GATA-motif	GATAGGG	828	7	+
GbEXPA23	G-box	CACGTG	876	6	+
GbEXPA23	TGACG-motif	TGACG	887	5	-
GbEXPA23	TGACG-motif	TGACG	1227	5	-
GbEXPA23	I-box	gGATAAGGTG	1362	9	-
GbEXPA24	GT1-motif	GGTTAAT	1857	7	+
GbEXPA24	ABRE	ACGTG	1525	5	-
GbEXPA24	CGTCA-motif	CGTCA	1290	5	-
GbEXPA24	G-Box	CACGTT	1525	6	+
GbEXPA24	O2-site	GATGACATGG	291	9	+
GbEXPA24	Box 4	ATTAAT	209	6	+
GbEXPA24	Box 4	ATTAAT	1823	6	-

GbEXPA24	TGACG-motif	TGACG	1290	5 +
GbEXPA24	AE-box	AGAAACTT	426	8 +
GbEXPA24	TCCC-motif	TCTCCCT	1201	7 -
GbEXPA24	GATA-motif	GATAGGG	255	7 +
GbEXPA24	TATC-box	TATCCCA	738	7 -
GbEXPA25	TGACG-motif	TGACG	1134	5 -
GbEXPA25	TGACG-motif	TGACG	1140	5 -
GbEXPA25	TGACG-motif	TGACG	1250	5 -
GbEXPA25	3-AF1 binding site	TAAGAGAGGA	629	10 +
GbEXPA25	TCCC-motif	TCTCCCT	1904	7 -
GbEXPA25	MRE	AACCTAA	100	7 +
GbEXPA25	MRE	AACCTAA	704	7 -
GbEXPA25	G-box	CACGTC	1592	6 +
GbEXPA25	TATC-box	TATCCCA	1839	7 +
GbEXPA25	ABRE	ACGTG	978	5 +
GbEXPA25	ABRE	ACGTG	1592	5 -
GbEXPA25	ACE	CTAACGTATT	342	9 -
GbEXPA25	GT1-motif	GGTTAAT	61	7 +
GbEXPA25	GT1-motif	GGTTAA	649	6 -
GbEXPA25	GT1-motif	GGTTAA	707	6 +
GbEXPA25	GT1-motif	GGTTAA	1698	6 +
GbEXPA25	P-box	CCTTTTG	983	7 +
GbEXPA25	MBS	CAACTG	1426	6 -
GbEXPA25	G-Box	CACGTT	977	6 -
GbEXPA25	CGTCA-motif	CGTCA	1134	5 +
GbEXPA25	CGTCA-motif	CGTCA	1140	5 +
GbEXPA25	CGTCA-motif	CGTCA	1250	5 +
GbEXPA25	Box 4	ATTAAT	1578	6 -
GbEXPA25	Box 4	ATTAAT	1916	6 -
GbEXPA26	GC-motif	CCCCCG	1311	6 -
GbEXPA26	LTR	CCGAAA	1308	6 -
GbEXPA26	LTR	CCGAAA	1472	6 +
GbEXPA26	TATC-box	TATCCCA	1839	7 +
GbEXPA26	chs-CMA1a	TTACTTAA	730	8 +
GbEXPA26	chs-CMA1a	TTACTTAA	1250	8 +
GbEXPA26	G-box	CACGTC	1594	6 +
GbEXPA26	GATA-motif	GATAGGG	558	7 -
GbEXPA26	AE-box	AGAAACAA	1556	8 -
GbEXPA26	TCCC-motif	TCTCCCT	1353	7 -
GbEXPA26	TCCC-motif	TCTCCCT	1904	7 -
GbEXPA26	GA-motif	ATAGATAA	958	8 +
GbEXPA26	TGACG-motif	TGACG	1106	5 -
GbEXPA26	TGACG-motif	TGACG	1112	5 -
GbEXPA26	Box 4	ATTAAT	718	6 +
GbEXPA26	Box 4	ATTAAT	1580	6 -
GbEXPA26	Box 4	ATTAAT	1916	6 -

GbEXPA26	4cl-CMA1b	ATTCCGATAA	1779	12 -
GbEXPA26	CAT-box	GCCACT	1460	6 -
GbEXPA26	CGTCA-motif	CGTCA	1106	5 +
GbEXPA26	CGTCA-motif	CGTCA	1112	5 +
GbEXPA26	G-Box	CACGTT	905	6 -
GbEXPA26	G-Box	CACGTT	936	6 -
GbEXPA26	P-box	CCTTTTG	396	7 -
GbEXPA26	P-box	CCTTTTG	942	7 +
GbEXPA26	GT1-motif	GGTTAA	1700	6 +
GbEXPA26	ABRE	ACGTG	906	5 +
GbEXPA26	ABRE	ACGTG	937	5 +
GbEXPA26	ABRE	ACGTG	1594	5 -
GbEXPA26	ACE	GACACGTATG	1222	9 -
GbEXPA27	AE-box	AGAAACAA	1560	8 -
GbEXPA27	GA-motif	ATAGATAA	673	8 +
GbEXPA27	GA-motif	ATAGATAA	1037	8 +
GbEXPA27	I-box	cGATAAGGCG	944	9 +
GbEXPA27	TGACG-motif	TGACG	178	5 +
GbEXPA27	TGACG-motif	TGACG	1115	5 -
GbEXPA27	TATC-box	TATCCCA	1842	7 +
GbEXPA27	G-box	TAAACGTG	983	8 +
GbEXPA27	G-box	CACGTC	1598	6 +
GbEXPA27	chs-CMA1a	TTACTTAA	805	8 +
GbEXPA27	chs-CMA1a	TTACTTAA	1259	8 +
GbEXPA27	P-box	CCTTTTG	471	7 -
GbEXPA27	P-box	CCTTTTG	991	7 +
GbEXPA27	P-box	CCTTTTG	1021	7 +
GbEXPA27	ABRE	ACGTG	986	5 +
GbEXPA27	ABRE	ACGTG	1016	5 +
GbEXPA27	ABRE	TACGGTC	1045	7 +
GbEXPA27	ABRE	ACGTG	1598	5 -
GbEXPA27	GT1-motif	GGTTAA	1704	6 +
GbEXPA27	CAT-box	GCCACT	1467	6 -
GbEXPA27	CAT-box	GCCACT	1471	6 +
GbEXPA27	ARE	AAACCA	1206	6 -
GbEXPA27	Box 4	ATTAAT	793	6 +
GbEXPA27	Box 4	ATTAAT	1584	6 -
GbEXPA27	Box 4	ATTAAT	1905	6 -
GbEXPA27	G-Box	CACGTT	985	6 -
GbEXPA27	G-Box	CACGTT	1015	6 -
GbEXPA27	CGTCA-motif	CGTCA	178	5 -
GbEXPA27	CGTCA-motif	CGTCA	1115	5 +
GbEXPA28	TGACG-motif	TGACG	677	5 +
GbEXPA28	TGACG-motif	TGACG	1123	5 -
GbEXPA28	TGACG-motif	TGACG	1129	5 -
GbEXPA28	A-box	CCGTCC	1055	6 +

GbEXPA28	TCCC-motif	TCTCCCT	1893	7 -
GbEXPA28	G-box	CACGAC	54	6 +
GbEXPA28	G-box	CACGTC	678	6 -
GbEXPA28	G-box	CACGTC	1583	6 +
GbEXPA28	chs-CMA1a	TTACTTAA	1271	8 +
GbEXPA28	TATC-box	TATCCCA	1830	7 +
GbEXPA28	ABRE	ACGTG	493	5 +
GbEXPA28	ABRE	ACGTG	679	5 +
GbEXPA28	ABRE	ACGTG	938	5 +
GbEXPA28	ABRE	ACGTG	1583	5 -
GbEXPA28	GT1-motif	GGTTAA	1689	6 +
GbEXPA28	TCA-element	CCATCTTTTT	507	9 -
GbEXPA28	TCA-element	CCATCTTTTT	539	9 -
GbEXPA28	P-box	CCTTTTG	973	7 +
GbEXPA28	G-Box	CACGTT	492	6 -
GbEXPA28	G-Box	CACGTT	937	6 -
GbEXPA28	CGTCA-motif	CGTCA	677	5 -
GbEXPA28	CGTCA-motif	CGTCA	1123	5 +
GbEXPA28	CGTCA-motif	CGTCA	1129	5 +
GbEXPA28	MBS	CAACTG	1419	6 -
GbEXPA28	O2-site	GATGACATGG	1982	9 -
GbEXPA28	CAT-box	GCCACT	596	6 -
GbEXPA28	CAT-box	GCCACT	689	6 +
GbEXPA28	Box 4	ATTAAT	46	6 +
GbEXPA28	Box 4	ATTAAT	1569	6 -
GbEXPA28	Box 4	ATTAAT	1905	6 -
GbEXPA29	GA-motif	ATAGATAA	444	8 +
GbEXPA29	I-box	GTATAAGGCC	254	9 +
GbEXPA29	I-box	cCATATCCAAT	1718	10 +
GbEXPA29	TGA-element	AACGAC	971	6 +
GbEXPA29	TGA-element	AACGAC	1632	6 +
GbEXPA29	AT-rich element	ATAGAAATCA	1544	10 +
GbEXPA29	GARE-motif	TCTGTTG	665	7 -
GbEXPA29	GARE-motif	TCTGTTG	1768	7 +
GbEXPA29	GATA-motif	AAGGATAAGG	431	9 +
GbEXPA29	GATA-motif	GATAGGA	1529	7 +
GbEXPA29	GATA-motif	GATAGGA	1650	7 +
GbEXPA29	G-box	TACGTG	946	6 -
GbEXPA29	G-box	CACGTC	1063	6 +
GbEXPA29	TCA-element	CCATCTTTTT	87	9 +
GbEXPA29	ABRE	ACGTG	946	5 -
GbEXPA29	ABRE	ACGTG	1063	5 -
GbEXPA29	Box 4	ATTAAT	1200	6 -
GbEXPA29	MBS	CAACTG	1481	6 -
GbEXPA29	ATCT-motif	AATCTAATCC	326	9 -
GbEXPA29	ATCT-motif	AATCTAATCC	1710	9 +

GbEXPA29	ATCT-motif	AATCTAATCC	1721	9 +
GbEXPA30	ATCT-motif	AATCTAATCC	1198	9 +
GbEXPA30	CGTCA-motif	CGTCA	1181	5 +
GbEXPA30	G-Box	CACGTT	758	6 +
GbEXPA30	G-Box	CACGTT	1017	6 -
GbEXPA30	Box 4	ATTAAT	824	6 +
GbEXPA30	Box 4	ATTAAT	1426	6 -
GbEXPA30	Box 4	ATTAAT	1558	6 -
GbEXPA30	TCT-motif	TCTTAC	628	6 -
GbEXPA30	O2-site	GATGA(C/T)(A/	792	8 -
GbEXPA30	GT1-motif	GTGTGTGAA	914	9 +
GbEXPA30	GT1-motif	GGTTAA	1671	6 +
GbEXPA30	ABRE	ACGTG	758	5 -
GbEXPA30	ABRE	ACGTG	1018	5 +
GbEXPA30	ABRE	ACGTG	1179	5 -
GbEXPA30	TGA-element	AACGAC	293	6 -
GbEXPA30	G-box	CACGTC	1179	6 +
GbEXPA30	3-AF3 binding site	CACTATCTAA(	545	10 +
GbEXPA30	TATC-box	TATCCCA	197	7 -
GbEXPA30	Unnamed_1	GGATTTTACA(	1148	11 +
GbEXPA30	TGACG-motif	TGACG	1181	5 -
GbEXPA30	GA-motif	ATAGATAA	645	8 -
GbEXPA30	AE-box	AGAAACAA	895	8 +
GbEXPA31	G-box	CACGAC	584	6 +
GbEXPA31	G-box	CACGTC	1033	6 +
GbEXPA31	G-box	TACGTG	1369	6 -
GbEXPA31	G-box	CACGAC	1434	6 -
GbEXPA31	G-box	TACGTG	1669	6 -
GbEXPA31	TGA-box	TGACGTAA	227	8 -
GbEXPA31	TGA-element	AACGAC	1446	6 +
GbEXPA31	LTR	CCGAAA	316	6 +
GbEXPA31	I-box	AAGATAAGGC	292	10 +
GbEXPA31	I-box	cCATATCCAAT	793	10 +
GbEXPA31	TGACG-motif	TGACG	230	5 -
GbEXPA31	TGACG-motif	TGACG	1035	5 -
GbEXPA31	TGACG-motif	TGACG	1577	5 -
GbEXPA31	CGTCA-motif	CGTCA	230	5 +
GbEXPA31	CGTCA-motif	CGTCA	1035	5 +
GbEXPA31	CGTCA-motif	CGTCA	1577	5 +
GbEXPA31	RY-element	CATGCATG	337	8 +
GbEXPA31	ACE	CTAACGTATT	129	9 -
GbEXPA31	ACE	GACACGTATG	831	9 +
GbEXPA31	ABRE	ACGTG	1033	5 -
GbEXPA31	ABRE	ACGTG	1369	5 -
GbEXPA31	ABRE	ACGTG	1669	5 -
GbEXPA32	GCN4_motif	TGAGTCA	727	7 -

GbEXPA32	ABRE	CGCACGTGTC	1401	9 -
GbEXPA32	ABRE	CACGTG	1403	6 -
GbEXPA32	ABRE	ACGTG	1404	5 +
GbEXPA32	ABRE	ACGTG	1764	5 -
GbEXPA32	CAT-box	GCCACT	276	6 +
GbEXPA32	TCT-motif	TCTTAC	357	6 -
GbEXPA32	TCT-motif	TCTTAC	1380	6 -
GbEXPA32	Box 4	ATTAAT	1237	6 -
GbEXPA32	G-Box	CACGTG	1403	6 -
GbEXPA32	CGTCA-motif	CGTCA	1582	5 +
GbEXPA32	ATCT-motif	AATCTAATCC	1388	9 -
GbEXPA32	TC-rich repeats	ATTCTCTAAC	534	9 +
GbEXPA32	TGACG-motif	TGACG	1582	5 -
GbEXPA32	G-box	CACGTG	1403	6 -
GbEXPA32	G-box	TACGTG	1764	6 -
GbEXPA32	GATA-motif	GATAGGA	632	7 +
GbEXLB1	GATA-motif	GATAGGA	1204	7 -
GbEXLB1	G-box	CACGTC	1750	6 -
GbEXLB1	chs-CMA2a	TCACTTGA	154	8 -
GbEXLB1	TGACG-motif	TGACG	1323	5 +
GbEXLB1	TGACG-motif	TGACG	1349	5 +
GbEXLB1	TGACG-motif	TGACG	1352	5 -
GbEXLB1	TGACG-motif	TGACG	1749	5 +
GbEXLB1	TC-rich repeats	GTTTTCTTAC	144	9 +
GbEXLB1	TC-rich repeats	ATTCTCTAAC	250	9 +
GbEXLB1	TC-rich repeats	ATTCTCTAAC	841	9 +
GbEXLB1	TC-rich repeats	ATTCTCTAAC	917	9 +
GbEXLB1	MBS	CAACTG	1556	6 -
GbEXLB1	G-Box	CACGTT	1490	6 -
GbEXLB1	CGTCA-motif	CGTCA	1323	5 -
GbEXLB1	CGTCA-motif	CGTCA	1349	5 -
GbEXLB1	CGTCA-motif	CGTCA	1352	5 +
GbEXLB1	CGTCA-motif	CGTCA	1749	5 -
GbEXLB1	CAT-box	GCCACT	74	6 +
GbEXLB1	TCT-motif	TCTTAC	148	6 +
GbEXLB1	TCT-motif	TCTTAC	1797	6 -
GbEXLB1	Box 4	ATTAAT	1245	6 -
GbEXLB1	ABRE	ACGTG	1491	5 +
GbEXLB1	ABRE	ACGTG	1751	5 +
GbEXLB1	GCN4_motif	TGAGTCA	1314	7 +
GbEXLA1	TGACG-motif	TGACG	393	5 +
GbEXLA1	TGACG-motif	TGACG	1383	5 -
GbEXLA1	TGACG-motif	TGACG	1393	5 +
GbEXLA1	G-box	GCCACGTGGA	778	9 -
GbEXLA1	G-box	TACGTG	780	6 +
GbEXLA1	G-box	TACGTG	1535	6 -

GbEXLA1	GT1-motif	GGTTAA	682	6 -
GbEXLA1	GT1-motif	GGTTAA	711	6 -
GbEXLA1	ABRE	TACGGTC	584	7 -
GbEXLA1	ABRE	ACGTG	781	5 +
GbEXLA1	ABRE	ACGTG	1535	5 -
GbEXLA1	GTGGC-motif	CAGCGTGTGG(	1748	10 +
GbEXLA1	A-box	CCGTCC	964	6 +
GbEXLA1	MRE	AACCTAA	331	7 +
GbEXLA1	MRE	AACCTAA	447	7 -
GbEXLA1	TGA-element	AACGAC	1039	6 +
GbEXLA1	TATC-box	TATCCCA	1462	7 -
GbEXLA1	AuxRR-core	GGTCCAT	581	7 -
GbEXLA1	CGTCA-motif	CGTCA	393	5 -
GbEXLA1	CGTCA-motif	CGTCA	1383	5 +
GbEXLA1	CGTCA-motif	CGTCA	1393	5 -
GbEXLA1	Box 4	ATTAAT	292	6 +
GbEXLA1	Box 4	ATTAAT	982	6 +
GbEXLA1	CAT-box	GCCACT	1055	6 +
GbEXLA1	TCT-motif	TCTTAC	381	6 +
GbEXLA1	TCT-motif	TCTTAC	869	6 +
GbEXLA2	TGACG-motif	TGACG	1778	5 -
GbEXLA2	I-box	cGATAAGGCG	1968	9 +
GbEXLA2	TC-rich repeats	GTTTTCTTAC	657	9 +
GbEXLA2	G-box	TAACACGTAG	389	9 -
GbEXLA2	G-box	TAACACGTAG	759	9 -
GbEXLA2	G-box	TAACACGTAG	1122	9 +
GbEXLA2	GT1-motif	GGTTAAT	67	7 +
GbEXLA2	TCA-element	TCAGAAGAGG	838	9 -
GbEXLA2	CGTCA-motif	CGTCA	1778	5 +
GbEXLA2	CAT-box	GCCACT	1275	6 -
GbEXLA2	Box 4	ATTAAT	569	6 +
GbEXLA2	Box 4	ATTAAT	1368	6 -
GbEXLA2	Box 4	ATTAAT	1497	6 -
GbEXLB2	ABRE	GCAACGTGTC	1568	9 -
GbEXLB2	ABRE	ACGTG	1570	5 -
GbEXLB2	ABRE	ACGTG	1741	5 +
GbEXLB2	ABRE	ACGTG	1976	5 +
GbEXLB2	P-box	CCTTTTG	692	7 -
GbEXLB2	G-Box	CACGTT	1570	6 +
GbEXLB2	G-Box	CACGTT	1740	6 -
GbEXLB2	HD-Zip 1	CAAT(A/T)ATT(	1013	8 -
GbEXLB2	CAT-box	GCCACT	1707	6 +
GbEXLB2	Box 4	ATTAAT	211	6 +
GbEXLB2	Box 4	ATTAAT	215	6 +
GbEXLB2	Box 4	ATTAAT	230	6 +
GbEXLB2	Box 4	ATTAAT	669	6 +

GbEXLB2	Box 4	ATTAAT	714	6 +
GbEXLB2	A-box	CCGTCC	702	6 +
GbEXLB2	G-box	TACGTG	1975	6 +
GbEXLB3	TGACG-motif	TGACG	1076	5 -
GbEXLB3	TC-rich repeats	ATTCTCTAAC	1246	9 -
GbEXLB3	TGA-element	AACGAC	544	6 -
GbEXLB3	GCN4_motif	TGAGTCA	664	7 -
GbEXLB3	GT1-motif	GGTTAA	85	6 -
GbEXLB3	GT1-motif	GGTTAA	201	6 -
GbEXLB3	GT1-motif	GGTTAAT	362	7 -
GbEXLB3	GT1-motif	GGTTAA	363	6 -
GbEXLB3	GT1-motif	GGTTAAT	811	7 +
GbEXLB3	GT1-motif	GGTTAAT	997	7 +
GbEXLB3	AuxRR-core	GGTCCAT	41	7 +
GbEXLB3	ABRE	ACGTG	1576	5 -
GbEXLB3	ABRE	ACGTG	1742	5 +
GbEXLB3	Box 4	ATTAAT	264	6 +
GbEXLB3	CAT-box	GCCACT	1708	6 +
GbEXLB3	ATCT-motif	AATCTAATCC	392	9 -
GbEXLB3	CGTCA-motif	CGTCA	1076	5 +
GbEXLB3	G-Box	CACGTT	1741	6 -
GbEXPA12	TGA-element	AACGAC	1320	6 -
GbEXPA12	G-box	TACGTG	78	6 -
GbEXPA12	G-box	CACGTC	1185	6 +
GbEXPA12	G-box	CACGTC	1379	6 +
GbEXPA12	AE-box	AGAAACTT	1365	8 +
GbEXPA12	TGACG-motif	TGACG	1187	5 -
GbEXPA12	TGACG-motif	TGACG	1627	5 +
GbEXPA12	TGACG-motif	TGACG	1664	5 -
GbEXPA12	Box 4	ATTAAT	274	6 +
GbEXPA12	Box 4	ATTAAT	587	6 +
GbEXPA12	Box 4	ATTAAT	961	6 +
GbEXPA12	Box 4	ATTAAT	1988	6 -
GbEXPA12	G-Box	CACGTT	444	6 +
GbEXPA12	G-Box	CACGTT	1916	6 -
GbEXPA12	CGTCA-motif	CGTCA	1187	5 +
GbEXPA12	CGTCA-motif	CGTCA	1627	5 -
GbEXPA12	CGTCA-motif	CGTCA	1664	5 +
GbEXPA12	ABRE	ACGTG	78	5 -
GbEXPA12	ABRE	ACGTG	444	5 -
GbEXPA12	ABRE	ACGTG	1185	5 -
GbEXPA12	ABRE	ACGTG	1379	5 -
GbEXPA12	ABRE	ACGTG	1917	5 +
GbEXPA12	GT1-motif	GGTTAA	1423	6 -
GbEXPA13	TCA-element	CCATCTTTTT	221	9 +
GbEXPA13	P-box	CCTTTTG	532	7 +

GbEXPA13	ABRE	ACGTG	1693	5 -
GbEXPA13	TCT-motif	TCTTAC	1851	6 +
GbEXPA13	Box 4	ATTAAT	215	6 +
GbEXPA13	Box 4	ATTAAT	690	6 +
GbEXPA13	Box 4	ATTAAT	886	6 +
GbEXPA13	Box 4	ATTAAT	1017	6 -
GbEXPA13	Box 4	ATTAAT	1915	6 -
GbEXPA13	G-Box	CACGTT	1693	6 +
GbEXPA13	CGTCA-motif	CGTCA	238	5 -
GbEXPA13	TGACG-motif	TGACG	238	5 +
GbEXPA13	TGA-element	AACGAC	1948	6 +
GbEXPA13	MRE	AACCTAA	1090	7 +
GbEXPA14	AuxRR-core	GGTCCAT	1076	7 -
GbEXPA14	GT1-motif	GGTTAA	742	6 -
GbEXPA14	ACE	CTAACGTATT	322	9 -
GbEXPA14	ABRE	ACGTG	749	5 -
GbEXPA14	ABRE	ACGTG	1416	5 +
GbEXPA14	GCN4_motif	TGAGTCA	1325	7 -
GbEXPA14	Sp1	GGGCGG	1258	6 +
GbEXPA14	P-box	CCTTTTG	649	7 +
GbEXPA14	CGTCA-motif	CGTCA	1374	5 +
GbEXPA14	CGTCA-motif	CGTCA	1379	5 -
GbEXPA14	CGTCA-motif	CGTCA	1405	5 -
GbEXPA14	CGTCA-motif	CGTCA	1411	5 +
GbEXPA14	CGTCA-motif	CGTCA	1719	5 +
GbEXPA14	G-Box	CACGTT	1415	6 -
GbEXPA14	Box 4	ATTAAT	641	6 +
GbEXPA14	Box 4	ATTAAT	684	6 +
GbEXPA14	Box 4	ATTAAT	987	6 +
GbEXPA14	Box 4	ATTAAT	1026	6 -
GbEXPA14	Box 4	ATTAAT	1976	6 -
GbEXPA14	TGACG-motif	TGACG	1374	5 -
GbEXPA14	TGACG-motif	TGACG	1379	5 +
GbEXPA14	TGACG-motif	TGACG	1405	5 +
GbEXPA14	TGACG-motif	TGACG	1411	5 -
GbEXPA14	TGACG-motif	TGACG	1719	5 -
GbEXPA14	G-box	TACGTG	749	6 -
GbEXPA14	GATA-motif	AAGGATAAGC	958	9 -
GbEXPA14	TATC-box	TATCCCA	196	7 +
GbEXLB4	TGACG-motif	TGACG	404	5 -
GbEXLB4	TGACG-motif	TGACG	1444	5 -
GbEXLB4	GA-motif	ATAGATAA	1348	8 +
GbEXLB4	G-box	TAACACGTAG	195	9 +
GbEXLB4	G-box	CACGAC	617	6 -
GbEXLB4	TGA-box	TGACGTAA	401	8 -
GbEXLB4	chs-CMA2a	TCACTTGA	704	8 +

GbEXLB4	CGTCA-motif	CGTCA	404	5 +
GbEXLB4	CGTCA-motif	CGTCA	1444	5 +
GbEXLB4	TCT-motif	TCTTAC	1024	6 +
GbEXLB4	TCT-motif	TCTTAC	1379	6 -
GbEXLB4	TCT-motif	TCTTAC	1784	6 +
GbEXLB4	CAT-box	GCCACT	1436	6 -
GbEXLB4	CAT-box	GCCACT	1566	6 -
GbEXLB4	Box 4	ATTAAT	965	6 +
GbEXLB4	Box 4	ATTAAT	1676	6 -
GbEXLB4	Box 4	ATTAAT	1709	6 -
GbEXLB5	GT1-motif	GGTTAA	638	6 -
GbEXLB5	ABRE	ACGTG	770	5 +
GbEXLB5	ABRE	GCAACGTGTC	1389	9 -
GbEXLB5	ABRE	ACGTG	1391	5 -
GbEXLB5	ABRE	GCAACGTGTC	1428	9 -
GbEXLB5	ABRE	ACGTG	1430	5 -
GbEXLB5	ABRE	ACGTG	1571	5 -
GbEXLB5	AuxRE	TGTCTCAATA/	198	11 +
GbEXLB5	TCA-element	CCATCTTTTT	618	9 -
GbEXLB5	TCA-element	TCAGAAGAGG	1329	9 +
GbEXLB5	CGTCA-motif	CGTCA	1180	5 +
GbEXLB5	CGTCA-motif	CGTCA	1489	5 -
GbEXLB5	CGTCA-motif	CGTCA	1543	5 +
GbEXLB5	CGTCA-motif	CGTCA	1803	5 +
GbEXLB5	G-Box	CACGTT	1391	6 +
GbEXLB5	G-Box	CACGTT	1430	6 +
GbEXLB5	TCT-motif	TCTTAC	811	6 -
GbEXLB5	TGACG-motif	TGACG	1180	5 -
GbEXLB5	TGACG-motif	TGACG	1489	5 +
GbEXLB5	TGACG-motif	TGACG	1543	5 -
GbEXLB5	TGACG-motif	TGACG	1803	5 -
GbEXLB5	G-box	TACGTG	769	6 +
GbEXLB5	G-box	TAACACGTAG	1427	9 +
GbEXLB5	G-box	CACGTC	1571	6 +
GbEXPA15	Box 4	ATTAAT	160	6 +
GbEXPA15	Box 4	ATTAAT	639	6 +
GbEXPA15	Box 4	ATTAAT	919	6 +
GbEXPA15	Box 4	ATTAAT	1209	6 -
GbEXPA15	Box 4	ATTAAT	1219	6 -
GbEXPA15	Box 4	ATTAAT	1657	6 -
GbEXPA15	G-Box	CACGTT	1733	6 +
GbEXPA15	CGTCA-motif	CGTCA	1312	5 -
GbEXPA15	CGTCA-motif	CGTCA	1749	5 -
GbEXPA15	TCA-element	CCATCTTTTT	266	9 +
GbEXPA15	ABRE	ACGTG	1199	5 -
GbEXPA15	ABRE	ACGTG	1733	5 -

GbEXPA15	GT1-motif	GGTTAAT	664	7 +
GbEXPA15	GT1-motif	GTGTGTGAA	1683	9 +
GbEXPA15	G-box	TACGTG	1199	6 -
GbEXPA15	TGA-element	AACGAC	1479	6 +
GbEXPA15	TC-rich repeats	GTTTTCTTAC	346	9 +
GbEXPA15	TGACG-motif	TGACG	1312	5 +
GbEXPA15	TGACG-motif	TGACG	1749	5 +
GbEXPA15	3-AF1 binding site	TAAGAGAGGA	615	10 -
GbEXLA3	GT1-motif	GGTTAA	350	6 +
GbEXLA3	CGTCA-motif	CGTCA	655	5 +
GbEXLA3	TCCC-motif	TCTCCCT	493	7 -
GbEXLA3	TC-rich repeats	ATTCTCTAAC	670	9 +
GbEXLA3	TGACG-motif	TGACG	655	5 -
GbEXLA4	TCT-motif	TCTTAC	1117	6 +
GbEXLA4	CGTCA-motif	CGTCA	890	5 -
GbEXLA4	G-Box	CACGTG	1876	6 -
GbEXLA4	P-box	CCTTTTG	412	7 -
GbEXLA4	GCN4_motif	TGAGTCA	100	7 +
GbEXLA4	GT1-motif	GGTTAA	1415	6 -
GbEXLA4	ABRE	ACGTG	1804	5 +
GbEXLA4	ABRE	GACACGTGGC	1874	9 -
GbEXLA4	ABRE	CACGTG	1876	6 -
GbEXLA4	ABRE	ACGTG	1877	5 +
GbEXLA4	GATA-motif	AAGGATAAGC	1197	9 -
GbEXLA4	G-box	CACGTC	1803	6 -
GbEXLA4	G-box	ACACGTGGC	1874	9 -
GbEXLA4	G-box	CACGTG	1876	6 -
GbEXLA4	TCCC-motif	TCTCCCT	1981	7 +
GbEXLA4	AE-box	AGAAACTT	710	8 -
GbEXLA4	A-box	CCGTCC	815	6 +
GbEXLA4	TGACG-motif	TGACG	890	5 +
GbEXLA4	I-box	GTATAAGGCC	452	9 -
GbEXLA5	TCCC-motif	TCTCCCT	304	7 -
GbEXLA5	GA-motif	ATAGATAA	185	8 +
GbEXLA5	TC-rich repeats	ATTCTCTAAC	338	9 +
GbEXLA5	TC-rich repeats	ATTCTCTAAC	1020	10 +
GbEXLA5	TGA-element	AACGAC	721	6 -
GbEXLA5	Box 4	ATTAAT	1423	6 -
GbEXLA5	TCT-motif	TCTTAC	242	6 -
GbEXLA5	CAT-box	GCCACT	498	6 -
GbEXLA5	MBS	CAACTG	951	6 -
GbEXLA5	CGTCA-motif	CGTCA	212	5 +
GbEXLA5	CGTCA-motif	CGTCA	820	5 -
GbEXLA5	CGTCA-motif	CGTCA	1463	5 +
GbEXLA5	TGACG-motif	TGACG	212	5 -
GbEXLA5	TGACG-motif	TGACG	820	5 +

GbEXLA5	TGACG-motif	TGACG	1463	5 -
GbEXLA5	GATA-motif	AAGGATAAGC	1138	9 -
GbEXLA5	GARE-motif	TCTGTTG	1976	7 +
GbEXLA5	G-box	CACGTC	210	6 +
GbEXLA5	G-box	CACGTC	1461	6 +
GbEXLA5	G-box	ACACGTGGC	1830	9 -
GbEXLA5	G-box	CACGTG	1832	6 -
GbEXLA5	P-box	CCTTTTG	924	7 -
GbEXLA5	P-box	CCTTTTG	991	7 +
GbEXLA5	TCA-element	CCATCTTTTT	1240	9 -
GbEXLA5	TCA-element	CCATCTTTTT	1349	9 -
GbEXLA5	TCA-element	CCATCTTTTT	1962	9 -
GbEXLA5	GT1-motif	GGTTAA	1291	6 -
GbEXLA5	GT1-motif	GGTTAA	1400	6 -
GbEXLA5	ABRE	ACGTG	210	5 -
GbEXLA5	ABRE	ACGTG	1461	5 -
GbEXLA5	ABRE	GACACGTGGC	1830	9 -
GbEXLA5	ABRE	CACGTG	1832	6 -
GbEXLA5	ABRE	ACGTG	1833	5 +
GbEXLA5	HD-Zip 1	CAAT(A/T)ATT[illegible]	626	8.5 +
GbEXLA5	G-Box	CACGTG	1832	6 -
GbEXPA16	Sp1	GGGCGG	1638	6 -
GbEXPA16	GCN4_motif	TGAGTCA	870	7 -
GbEXPA16	LAMP-element	CTTTATCA	28	8 +
GbEXPA16	ABRE	ACGTG	690	5 +
GbEXPA16	GT1-motif	GGTTAAT	378	7 +
GbEXPA16	GT1-motif	GTGTGTGAA	692	9 +
GbEXPA16	GT1-motif	GGTTAA	1017	6 +
GbEXPA16	TCT-motif	TCTTAC	849	6 +
GbEXPA16	CAT-box	GCCACT	1464	6 -
GbEXPA16	CAT-box	GCCACT	1535	6 -
GbEXPA16	CAT-box	GCCACT	1607	6 -
GbEXPA16	Box 4	ATTAAT	1268	6 -
GbEXPA16	CGTCA-motif	CGTCA	1496	5 +
GbEXPA16	TGACG-motif	TGACG	1496	5 -
GbEXPA16	TGA-element	AACGAC	466	6 +
GbEXPA16	GATA-motif	AAGGATAAGC	737	9 -
GbEXPA16	GATA-motif	GATAGGA	1030	7 -
GbEXPA16	MRE	AACCTAA	344	7 +
GbEXPA16	G-box	CACGTC	689	6 -
GbEXPA17	G-box	TACGTG	1525	6 -
GbEXPA17	ATC-motif	AGTAATCT	1614	8 +
GbEXPA17	ATC-motif	AGTAATCT	1772	8 -
GbEXPA17	TGACG-motif	TGACG	781	5 +
GbEXPA17	TGACG-motif	TGACG	1224	5 -
GbEXPA17	CGTCA-motif	CGTCA	781	5 -

GbEXPA17	CGTCA-motif	CGTCA	1224	5	+
GbEXPA17	CAT-box	GCCACT	517	6	-
GbEXPA17	CAT-box	GCCACT	521	6	+
GbEXPA17	CAT-box	GCCACT	1415	6	+
GbEXPA17	Box 4	ATTAAT	103	6	+
GbEXPA17	Box 4	ATTAAT	1286	6	-
GbEXPA17	ABRE	CGTACGTGCA	1523	10	-
GbEXPA17	ABRE	ACGTG	1525	5	-
GbEXPA17	GT1-motif	GGTTAAT	1051	7	-
GbEXPA17	GT1-motif	GGTTAA	1052	6	-
GbEXPA17	GT1-motif	GGTTAAT	1192	7	-
GbEXPA17	GT1-motif	GGTTAA	1193	6	-
GbEXPA18	Box 4	ATTAAT	1440	6	-
GbEXPA18	Box 4	ATTAAT	1837	6	-
GbEXPA18	TCT-motif	TCTTAC	203	6	+
GbEXPA18	CGTCA-motif	CGTCA	1959	5	+
GbEXPA18	Sp1	GGGCGG	85	6	+
GbEXPA18	ACE	GACACGTATG	1218	9	-
GbEXPA18	MRE	AACCTAA	1128	7	+
GbEXPA18	ATC-motif	AGTAATCT	1371	8	-
GbEXPB2	ACE	GACACGTATG	793	9	-
GbEXPB2	TATC-box	TATCCCA	466	7	+
GbEXPB2	MRE	AACCTAA	343	7	-
GbEXPB2	G-Box	CACGTT	1023	6	+
GbEXPB2	GCN4_motif	TGAGTCA	349	7	+
GbEXPB2	AACA_motif	TAACAAACTC	34	11	-
GbEXPB2	AACA_motif	TAACAAACTC	970	11	+
GbEXPB2	P-box	CCTTTTG	109	7	-
GbEXPB2	ABRE	ACGTG	1023	5	-
GbEXPB2	GT1-motif	GGTTAA	453	6	-
GbEXPB2	GT1-motif	GGTTAA	765	6	-
GbEXPB2	GT1-motif	GGTTAA	1541	6	-
GbEXPB2	G-box	CACGAC	1532	6	+
GbEXPB2	TGACG-motif	TGACG	324	5	+
GbEXPB2	TGACG-motif	TGACG	714	5	-
GbEXPB3	G-Box	CACGTG	602	6	+
GbEXPB3	G-Box	CACGTT	1419	6	+
GbEXPB3	CGTCA-motif	CGTCA	1730	5	-
GbEXPB3	ATCT-motif	AATCTAATCC	533	9	+
GbEXPB3	Box 4	ATTAAT	460	6	+
GbEXPB3	RY-element	CATGCATG	971	8	+
GbEXPB3	ABRE	CACGTG	602	6	+
GbEXPB3	ABRE	ACGTG	603	5	+
GbEXPB3	ABRE	ACGTG	915	5	-
GbEXPB3	ABRE	ACGTG	1419	5	-
GbEXPB3	GT1-motif	GGTTAAT	1293	7	-

GbEXPB3	GT1-motif	GGTTAA	1294	6	-
GbEXPB3	TCA-element	CCATCTTTTT	755	9	-
GbEXPB3	Sp1	GGGCGG	764	6	+
GbEXPB3	G-box	CACGTG	602	6	+
GbEXPB3	G-box	TACGTG	915	6	-
GbEXPB3	TGA-element	AACGAC	1677	6	+
GbEXPB3	TGA-element	AACGAC	1816	6	+
GbEXPB3	TGACG-motif	TGACG	1730	5	+
GbEXPB3	3-AF1 binding site	TAAGAGAGGA	726	10	-
GbEXPB4	TATC-box	TATCCCA	1882	7	+
GbEXPB4	TGA-element	AACGAC	1188	6	+
GbEXPB4	G-box	CACGTC	1750	6	+
GbEXPB4	A-box	CCGTCC	748	6	-
GbEXPB4	A-box	CCGTCC	771	6	+
GbEXPB4	TGACG-motif	TGACG	371	5	-
GbEXPB4	TGACG-motif	TGACG	1752	5	-
GbEXPB4	I-box	GTATAAGGCC	1760	9	+
GbEXPB4	TCT-motif	TCTTAC	542	6	+
GbEXPB4	CGTCA-motif	CGTCA	371	5	+
GbEXPB4	CGTCA-motif	CGTCA	1752	5	+
GbEXPB4	TCA-element	CCATCTTTTT	161	9	+
GbEXPB4	TCA-element	TCAGAAGAGG	851	9	-
GbEXPB4	TCA-element	TCAGAAGAGG	1677	10	-
GbEXPB4	ACE	CTAACGTATT	363	9	-
GbEXPB4	ABRE	ACGTG	1750	5	-
GbEXPB4	AuxRR-core	GGTCCAT	1670	7	-
GbEXPB4	GT1-motif	GGTTAA	109	6	-

Petroselinum crispum	part of a conserved DNA module involved in light responsiveness
Zea mays	cis-acting regulatory element essential for the anaerobic induction
Zea mays	cis-acting regulatory element essential for the anaerobic induction
Arabidopsis thaliana	part of a light responsive element
Hordeum vulgare	cis-acting regulatory element involved in the MeJA-responsiveness
Oryza sativa	cis-regulatory element involved in endosperm expression
Oryza sativa	gibberellin-responsive element
Oryza sativa	gibberellin-responsive element
Arabidopsis thaliana	light responsive element
Arabidopsis thaliana	light responsive element
Arabidopsis thaliana	light responsive element
Nicotiana tabacum	cis-acting regulatory element involved in auxin responsiveness
Arabidopsis thaliana	cis-acting element involved in the abscisic acid responsiveness
Arabidopsis thaliana	cis-acting regulatory element involved in light responsiveness
Solanum tuberosum	part of a light responsive element
Petroselinum crispum	MYB binding site involved in light responsiveness
Pisum sativum	common cis-acting element in promoter and enhancer regions
Nicotiana tabacum	cis-acting element involved in defense and stress responsiveness
Hordeum vulgare	cis-acting regulatory element involved in the MeJA-responsiveness
Petroselinum crispum	cis-acting element involved in light responsiveness
Petroselinum crispum	cis-acting element involved in light responsiveness
Arabidopsis thaliana	cis-acting element involved in the abscisic acid responsiveness
Arabidopsis thaliana	cis-acting element involved in the abscisic acid responsiveness
Arabidopsis thaliana	cis-acting element involved in the abscisic acid responsiveness
Arabidopsis thaliana	cis-acting element involved in the abscisic acid responsiveness
Oryza sativa	gibberellin-responsive element
Pisum sativum	part of a conserved DNA module involved in light responsiveness
Pisum sativum	cis-acting regulatory element involved in light responsiveness
Pisum sativum	cis-acting regulatory element involved in light responsiveness
Hordeum vulgare	cis-acting regulatory element involved in the MeJA-responsiveness
Arabidopsis thaliana	part of a light responsive element
Arabidopsis thaliana	part of a light responsive element
Helianthus annuus	cis-acting regulatory element involved in seed-specific regulation
Petroselinum crispum	part of a conserved DNA module involved in light responsiveness
Petroselinum crispum	part of a conserved DNA module involved in light responsiveness
Arabidopsis thaliana	element involved in differentiation of the palisade mesophyll cells
Arabidopsis thaliana	part of a light responsive element
Solanum tuberosum	part of a light responsive element
Hordeum vulgare	cis-acting regulatory element involved in the MeJA-responsiveness
Flaveria trinervia	part of a light responsive element
Nicotiana tabacum	cis-acting element involved in defense and stress responsiveness
Nicotiana tabacum	cis-acting element involved in defense and stress responsiveness
Arabidopsis thaliana	part of a module for light response
Petroselinum crispum	cis-acting regulatory element
Petroselinum crispum	MYB binding site involved in light responsiveness
Petroselinum crispum	MYB binding site involved in light responsiveness

Arabidopsis thaliana	cis-acting regulatory element involved in light responsiveness
Arabidopsis thaliana	cis-acting regulatory element involved in light responsiveness
Arabidopsis thaliana	cis-acting regulatory element involved in light responsiveness
Hordeum vulgare	cis-acting element involved in low-temperature responsiveness
Brassica oleracea	gibberellin-responsive element
Glycine max	binding site of AT-rich DNA binding protein (ATBP-1)
Brassica oleracea	cis-acting regulatory element involved in light responsiveness
Zea mays	cis-acting regulatory element involved in light responsiveness
Zea mays	cis-acting regulatory element involved in light responsiveness
Nicotiana tabacum	cis-acting element involved in defense and stress responsiveness
Petroselinum crispum	part of a conserved DNA module involved in light responsiveness
Petroselinum crispum	part of a conserved DNA module involved in light responsiveness
Petroselinum crispum	part of a conserved DNA module involved in light responsiveness
Petroselinum crispum	part of a conserved DNA module involved in light responsiveness
Pisum sativum	cis-acting regulatory element involved in light responsiveness
Pisum sativum	cis-acting regulatory element involved in light responsiveness
Brassica oleracea	cis-acting element involved in salicylic acid responsiveness
Oryza sativa	cis-regulatory element involved in endosperm expression
Arabidopsis thaliana	cis-acting element involved in the abscisic acid responsiveness
Arabidopsis thaliana	cis-acting element involved in the abscisic acid responsiveness
Arabidopsis thaliana	cis-acting element involved in the abscisic acid responsiveness
Hordeum vulgare	cis-acting element involved in the abscisic acid responsiveness
Nicotiana tabacum	cis-acting regulatory element involved in auxin responsiveness
Nicotiana tabacum	cis-acting regulatory element involved in auxin responsiveness
Petroselinum crispum	part of a conserved DNA module involved in light responsiveness
Petroselinum crispum	part of a conserved DNA module involved in light responsiveness
Petroselinum crispum	part of a conserved DNA module involved in light responsiveness
Petroselinum crispum	part of a conserved DNA module involved in light responsiveness
Pisum sativum	cis-acting regulatory element involved in light responsiveness
Pisum sativum	cis-acting regulatory element involved in light responsiveness
Oryza sativa	cis-regulatory element involved in endosperm expression
Brassica oleracea	cis-acting element involved in salicylic acid responsiveness
Nicotiana tabacum	cis-acting regulatory element involved in auxin responsiveness
Nicotiana tabacum	cis-acting regulatory element involved in auxin responsiveness
Arabidopsis thaliana	cis-acting element involved in the abscisic acid responsiveness
Arabidopsis thaliana	cis-acting element involved in the abscisic acid responsiveness
Arabidopsis thaliana	cis-acting element involved in the abscisic acid responsiveness
Hordeum vulgare	cis-acting element involved in the abscisic acid responsiveness
Glycine max	binding site of AT-rich DNA binding protein (ATBP-1)
Brassica oleracea	gibberellin-responsive element
Brassica oleracea	cis-acting regulatory element involved in light responsiveness
Zea mays	cis-acting regulatory element involved in light responsiveness
Zea mays	cis-acting regulatory element involved in light responsiveness
Petroselinum crispum	cis-acting regulatory element
Arabidopsis thaliana	part of a module for light response
Petroselinum crispum	cis-acting regulatory element

Hordeum vulgare	cis-acting regulatory element involved in the MeJA-responsiveness
Hordeum vulgare	cis-acting regulatory element involved in the MeJA-responsiveness
Hordeum vulgare	cis-acting regulatory element involved in the MeJA-responsiveness
Arabidopsis thaliana	cis-acting regulatory element involved in light responsiveness
Daucus carota	part of a light responsive element
Petroselinum crispum	MYB binding site involved in light responsiveness
Nicotiana tabacum	cis-acting element involved in salicylic acid responsiveness
Arabidopsis thaliana	cis-acting element involved in the abscisic acid responsiveness
Nicotiana tabacum	cis-acting regulatory element involved in auxin responsiveness
Arabidopsis thaliana	part of a light responsive element
Solanum tuberosum	part of a light responsive module
Zea mays	cis-acting regulatory element essential for the anaerobic induction
Zea mays	cis-acting regulatory element essential for the anaerobic induction
Zea mays	cis-acting regulatory element essential for the anaerobic induction
Petroselinum crispum	part of a conserved DNA module involved in light responsiveness
Hordeum vulgare	cis-acting regulatory element involved in the MeJA-responsiveness
Hordeum vulgare	cis-acting regulatory element involved in the MeJA-responsiveness
Hordeum vulgare	cis-acting regulatory element involved in the MeJA-responsiveness
Arabidopsis thaliana	MYB binding site involved in drought-inducibility
Glycine max	binding site of AT-rich DNA binding protein (ATBP-1)
Zea mays	cis-acting regulatory element involved in light responsiveness
Hordeum vulgare	cis-acting regulatory element involved in the MeJA-responsiveness
Hordeum vulgare	cis-acting regulatory element involved in the MeJA-responsiveness
Hordeum vulgare	cis-acting regulatory element involved in the MeJA-responsiveness
Spinacia oleracea	part of a light responsive element
Arabidopsis thaliana	MYB binding site involved in drought-inducibility
Arabidopsis thaliana	MYB binding site involved in drought-inducibility
Arabidopsis thaliana	MYB binding site involved in drought-inducibility
Pisum sativum	cis-acting regulatory element involved in light responsiveness
Hordeum vulgare	cis-acting regulatory element involved in the MeJA-responsiveness
Hordeum vulgare	cis-acting regulatory element involved in the MeJA-responsiveness
Hordeum vulgare	cis-acting regulatory element involved in the MeJA-responsiveness
Petroselinum crispum	part of a conserved DNA module involved in light responsiveness
Petroselinum crispum	part of a conserved DNA module involved in light responsiveness
Petroselinum crispum	part of a conserved DNA module involved in light responsiveness
Petroselinum crispum	part of a conserved DNA module involved in light responsiveness
Arabidopsis thaliana	element involved in differentiation of the palisade mesophyll cells
Arabidopsis thaliana	cis-acting element involved in the abscisic acid responsiveness
Arabidopsis thaliana	cis-acting element involved in the abscisic acid responsiveness
Oryza sativa	gibberellin-responsive element
Petroselinum crispum	part of a conserved DNA module involved in light responsiveness
Petroselinum crispum	part of a conserved DNA module involved in light responsiveness
Petroselinum crispum	part of a conserved DNA module involved in light responsiveness
Petroselinum crispum	part of a conserved DNA module involved in light responsiveness
Pisum sativum	cis-acting regulatory element involved in light responsiveness
Pisum sativum	cis-acting regulatory element involved in light responsiveness

Arabidopsis thaliana	MYB binding site involved in drought-inducibility
Oryza sativa	cis-regulatory element involved in endosperm expression
Brassica oleracea	cis-acting element involved in salicylic acid responsiveness
Nicotiana tabacum	cis-acting regulatory element involved in auxin responsiveness
Nicotiana tabacum	cis-acting regulatory element involved in auxin responsiveness
Arabidopsis thaliana	cis-acting element involved in the abscisic acid responsiveness
Arabidopsis thaliana	cis-acting element involved in the abscisic acid responsiveness
Arabidopsis thaliana	cis-acting element involved in the abscisic acid responsiveness
Hordeum vulgare	cis-acting element involved in the abscisic acid responsiveness
Brassica oleracea	cis-acting regulatory element involved in light responsiveness
Zea mays	cis-acting regulatory element involved in light responsiveness
Zea mays	cis-acting regulatory element involved in light responsiveness
Brassica oleracea	gibberellin-responsive element
Hordeum vulgare	cis-acting regulatory element involved in the MeJA-responsiveness
Arabidopsis thaliana	cis-acting regulatory element involved in light responsiveness
Solanum tuberosum	part of a light responsive element
Solanum tuberosum	part of a light responsive element
Brassica oleracea	gibberellin-responsive element
Hordeum vulgare	cis-acting element involved in low-temperature responsiveness
Solanum tuberosum	light responsive element
Arabidopsis thaliana	light responsive element
Hordeum vulgare	cis-acting element involved in the abscisic acid responsiveness
Arabidopsis thaliana	cis-acting element involved in the abscisic acid responsiveness
Arabidopsis thaliana	cis-acting element involved in the abscisic acid responsiveness
Hordeum vulgare	cis-acting regulatory element involved in the MeJA-responsiveness
Pisum sativum	cis-acting regulatory element involved in light responsiveness
Arabidopsis thaliana	MYB binding site involved in drought-inducibility
Arabidopsis thaliana	MYB binding site involved in drought-inducibility
Zea mays	cis-acting regulatory element involved in zein metabolism regulation
Arabidopsis thaliana	part of a light responsive element
Petroselinum crispum	part of a conserved DNA module involved in light responsiveness
Petroselinum crispum	part of a conserved DNA module involved in light responsiveness
Petroselinum crispum	part of a conserved DNA module involved in light responsiveness
Petroselinum crispum	part of a conserved DNA module involved in light responsiveness
Petroselinum crispum	part of a conserved DNA module involved in light responsiveness
Petroselinum crispum	gibberellin-responsive element and part of a light responsive element
Oryza sativa	involved in endosperm-specific negative expression
Oryza sativa	cis-regulatory element involved in endosperm expression
Nicotiana tabacum	cis-acting regulatory element involved in auxin responsiveness
Nicotiana tabacum	cis-acting regulatory element involved in auxin responsiveness
Avena sativa	light responsive element
Arabidopsis thaliana	light responsive element
Arabidopsis thaliana	cis-acting element involved in the abscisic acid responsiveness
Petroselinum crispum	part of a conserved DNA module involved in light responsiveness
Arabidopsis thaliana	cis-acting regulatory element related to meristem expression
Hordeum vulgare	cis-acting regulatory element involved in the MeJA-responsiveness

Hordeum vulgare	cis-acting regulatory element involved in the MeJA-responsiveness
Hordeum vulgare	cis-acting regulatory element involved in the MeJA-responsiveness
Hordeum vulgare	cis-acting regulatory element involved in the MeJA-responsiveness
Hordeum vulgare	cis-acting regulatory element involved in the MeJA-responsiveness
Hordeum vulgare	cis-acting regulatory element involved in the MeJA-responsiveness
Nicotiana tabacum	cis-acting element involved in defense and stress responsiveness
Brassica oleracea	auxin-responsive element
Zea mays	cis-acting regulatory element involved in light responsiveness
Zea mays	cis-acting regulatory element involved in light responsiveness
Zea mays	cis-acting regulatory element involved in light responsiveness
Hordeum vulgare	cis-acting regulatory element involved in the MeJA-responsiveness
Hordeum vulgare	cis-acting regulatory element involved in the MeJA-responsiveness
Hordeum vulgare	cis-acting regulatory element involved in the MeJA-responsiveness
Brassica oleracea	auxin-responsive element
Brassica oleracea	gibberellin-responsive element
Arabidopsis thaliana	cis-acting regulatory element involved in light responsiveness
Arabidopsis thaliana	cis-acting regulatory element involved in light responsiveness
Oryza sativa	gibberellin-responsive element
Petroselinum crispum	cis-acting element involved in light responsiveness
Arabidopsis thaliana	cis-acting element involved in the abscisic acid responsiveness
Arabidopsis thaliana	cis-acting element involved in the abscisic acid responsiveness
Oryza sativa	cis-acting element involved in the abscisic acid responsiveness
Oryza sativa	cis-acting element involved in the abscisic acid responsiveness
Arabidopsis thaliana	cis-acting element involved in the abscisic acid responsiveness
Arabidopsis thaliana	cis-acting element involved in the abscisic acid responsiveness
Arabidopsis thaliana	cis-acting element involved in the abscisic acid responsiveness
Petroselinum crispum	part of a conserved DNA module involved in light responsiveness
Petroselinum crispum	part of a conserved DNA module involved in light responsiveness
Petroselinum crispum	part of a conserved DNA module involved in light responsiveness
Petroselinum crispum	part of a conserved DNA module involved in light responsiveness
Arabidopsis thaliana	cis-acting regulatory element related to meristem expression
Arabidopsis thaliana	part of a light responsive element
Arabidopsis thaliana	part of a light responsive element
Zea mays	cis-acting regulatory element essential for the anaerobic induction
Zea mays	cis-acting regulatory element involved in zein metabolism regulation
Pisum sativum	part of a conserved DNA module involved in light responsiveness
Pisum sativum	part of a conserved DNA module involved in light responsiveness
Hordeum vulgare	cis-acting regulatory element involved in the MeJA-responsiveness
Hordeum vulgare	cis-acting regulatory element involved in the MeJA-responsiveness
Hordeum vulgare	cis-acting regulatory element involved in the MeJA-responsiveness
Pisum sativum	cis-acting regulatory element involved in light responsiveness
Pisum sativum	cis-acting regulatory element involved in light responsiveness
Pisum sativum	cis-acting regulatory element involved in light responsiveness
Arabidopsis thaliana	cis-acting regulatory element involved in light responsiveness
Arabidopsis thaliana	cis-acting regulatory element involved in light responsiveness
Brassica oleracea	cis-acting regulatory element involved in light responsiveness

Zea mays	cis-acting regulatory element involved in light responsiveness
Larix laricina	part of a light responsive element
Solanum tuberosum	part of a light responsive element
Hordeum vulgare	cis-acting regulatory element involved in the MeJA-responsiveness
Hordeum vulgare	cis-acting regulatory element involved in the MeJA-responsiveness
Hordeum vulgare	cis-acting regulatory element involved in the MeJA-responsiveness
Petroselinum crispum	part of a conserved DNA module involved in light responsiveness
Petroselinum crispum	part of a conserved DNA module involved in light responsiveness
Zea mays	cis-acting regulatory element essential for the anaerobic induction
Zea mays	cis-acting regulatory element essential for the anaerobic induction
Zea mays	cis-acting regulatory element essential for the anaerobic induction
Hordeum vulgare	cis-acting regulatory element involved in the MeJA-responsiveness
Hordeum vulgare	cis-acting regulatory element involved in the MeJA-responsiveness
Hordeum vulgare	cis-acting regulatory element involved in the MeJA-responsiveness
Pisum sativum	cis-acting regulatory element involved in light responsiveness
Pisum sativum	cis-acting regulatory element involved in light responsiveness
Arabidopsis thaliana	light responsive element
Arabidopsis thaliana	cis-acting element involved in the abscisic acid responsiveness
Triticum aestivum	cis-acting element involved in the abscisic acid responsiveness
Arabidopsis thaliana	cis-acting element involved in the abscisic acid responsiveness
Arabidopsis thaliana	cis-acting element involved in the abscisic acid responsiveness
Arabidopsis thaliana	cis-acting element involved in the abscisic acid responsiveness
Arabidopsis thaliana	cis-acting element involved in the abscisic acid responsiveness
Zea mays	cis-acting regulatory element involved in zein metabolism regulation
Zea mays	cis-acting regulatory element involved in zein metabolism regulation
Zea mays	cis-acting regulatory element involved in zein metabolism regulation
Arabidopsis thaliana	cis-acting regulatory element related to meristem expression
Helianthus annuus	cis-acting regulatory element involved in seed-specific regulation
Petroselinum crispum	part of a conserved DNA module involved in light responsiveness
Petroselinum crispum	part of a conserved DNA module involved in light responsiveness
Petroselinum crispum	part of a conserved DNA module involved in light responsiveness
Pisum sativum	cis-acting regulatory element involved in light responsiveness
Pisum sativum	cis-acting regulatory element involved in light responsiveness
Hordeum vulgare	cis-acting regulatory element involved in the MeJA-responsiveness
Hordeum vulgare	cis-acting regulatory element involved in the MeJA-responsiveness
Hordeum vulgare	cis-acting regulatory element involved in the MeJA-responsiveness
Arabidopsis thaliana	MYB binding site involved in drought-inducibility
Arabidopsis thaliana	MYB binding site involved in drought-inducibility
Arabidopsis thaliana	MYB binding site involved in drought-inducibility
Arabidopsis thaliana	MYB binding site involved in drought-inducibility
Arabidopsis thaliana	cis-acting element involved in the abscisic acid responsiveness
Arabidopsis thaliana	cis-acting element involved in the abscisic acid responsiveness
Arabidopsis thaliana	cis-acting element involved in the abscisic acid responsiveness
Arabidopsis thaliana	light responsive element
Arabidopsis thaliana	light responsive element
Arabidopsis thaliana	light responsive element

Arabidopsis thaliana	light responsive element
Hordeum vulgare	cis-acting element involved in low-temperature responsiveness
Zea mays	enhancer-like element involved in anoxic specific inducibility
Zea mays	enhancer-like element involved in anoxic specific inducibility
Arabidopsis thaliana	cis-acting regulatory element involved in light responsiveness
Arabidopsis thaliana	cis-acting regulatory element involved in light responsiveness
Brassica oleracea	auxin-responsive element
Brassica oleracea	auxin-responsive element
Solanum tuberosum	part of a light responsive element
Petroselinum crispum	cis-acting regulatory element
Zea mays	part of a light responsive element
Nicotiana tabacum	cis-acting element involved in defense and stress responsiveness
Nicotiana tabacum	cis-acting element involved in defense and stress responsiveness
Hordeum vulgare	cis-acting regulatory element involved in the MeJA-responsiveness
Hordeum vulgare	cis-acting regulatory element involved in the MeJA-responsiveness
Hordeum vulgare	cis-acting regulatory element involved in the MeJA-responsiveness
Arabidopsis thaliana	part of a conserved DNA module involved in light responsiveness
Arabidopsis thaliana	part of a light response element
Daucus carota	part of a light responsive element
Zea mays	cis-acting regulatory element involved in light responsiveness
Arabidopsis thaliana	cis-acting regulatory element involved in light responsiveness
Solanum tuberosum	part of a light responsive element
Brassica oleracea	auxin-responsive element
Nicotiana tabacum	cis-acting element involved in salicylic acid responsiveness
Oryza sativa	gibberellin-responsive element
Arabidopsis thaliana	light responsive element
Arabidopsis thaliana	cis-acting element involved in the abscisic acid responsiveness
Arabidopsis thaliana	cis-acting element involved in the abscisic acid responsiveness
Arabidopsis thaliana	cis-acting element involved in the abscisic acid responsiveness
Zea mays	cis-acting regulatory element involved in zein metabolism regulation
Petroselinum crispum	part of a conserved DNA module involved in light responsiveness
Petroselinum crispum	part of a conserved DNA module involved in light responsiveness
Arabidopsis thaliana	cis-acting regulatory element related to meristem expression
Arabidopsis thaliana	cis-acting regulatory element related to meristem expression
Arabidopsis thaliana	part of a light responsive element
Pisum sativum	cis-acting regulatory element involved in light responsiveness
Spinacia oleracea	part of a light responsive element
Hordeum vulgare	cis-acting regulatory element involved in the MeJA-responsiveness
Nicotiana tabacum	cis-acting element involved in defense and stress responsiveness
Hordeum vulgare	cis-acting element involved in low-temperature responsiveness
Hordeum vulgare	cis-acting element involved in low-temperature responsiveness
Brassica oleracea	auxin-responsive element
Zea mays	cis-acting regulatory element involved in light responsiveness
Oryza sativa	gibberellin-responsive element
Nicotiana tabacum	cis-acting regulatory element involved in auxin responsiveness
Arabidopsis thaliana	cis-acting element involved in the abscisic acid responsiveness

Petroselinum crispum	part of a conserved DNA module involved in light responsiveness
Arabidopsis thaliana	cis-acting regulatory element related to meristem expression
Hordeum vulgare	cis-acting regulatory element involved in the MeJA-responsiveness
Pisum sativum	cis-acting regulatory element involved in light responsiveness
Arabidopsis thaliana	cis-acting regulatory element involved in light responsiveness
Hordeum vulgare	cis-acting regulatory element involved in the MeJA-responsiveness
Nicotiana tabacum	cis-acting element involved in defense and stress responsiveness
Flaveria trinervia	part of a light responsive element
Hordeum vulgare	cis-acting regulatory element involved in the MeJA-responsiveness
Petroselinum crispum	part of a conserved DNA module involved in light responsiveness
Petroselinum crispum	part of a conserved DNA module involved in light responsiveness
Petroselinum crispum	part of a conserved DNA module involved in light responsiveness
Petroselinum crispum	part of a conserved DNA module involved in light responsiveness
Petroselinum crispum	part of a conserved DNA module involved in light responsiveness
Arabidopsis thaliana	cis-acting regulatory element related to meristem expression
Arabidopsis thaliana	cis-acting regulatory element related to meristem expression
Zea mays	cis-acting regulatory element involved in zein metabolism regulation
Avena sativa	light responsive element
Arabidopsis thaliana	light responsive element
Triticum aestivum	cis-acting element involved in the abscisic acid responsiveness
Arabidopsis thaliana	cis-acting element involved in the abscisic acid responsiveness
Oryza sativa	cis-regulatory element involved in endosperm expression
Hordeum vulgare	cis-acting regulatory element involved in the MeJA-responsiveness
Hordeum vulgare	cis-acting regulatory element involved in the MeJA-responsiveness
Pisum sativum	cis-acting regulatory element involved in light responsiveness
Pisum sativum	cis-acting regulatory element involved in light responsiveness
Petroselinum crispum	part of a conserved DNA module involved in light responsiveness
Petroselinum crispum	part of a conserved DNA module involved in light responsiveness
Arabidopsis thaliana	cis-acting regulatory element related to meristem expression
Arabidopsis thaliana	light responsive element
Arabidopsis thaliana	cis-acting element involved in the abscisic acid responsiveness
Arabidopsis thaliana	cis-acting element involved in the abscisic acid responsiveness
Arabidopsis thaliana	cis-acting element involved in the abscisic acid responsiveness
Oryza sativa	light responsive element
Pisum sativum	part of a light responsive element
Arabidopsis thaliana	cis-acting regulatory element involved in light responsiveness
Hordeum vulgare	cis-acting regulatory element involved in the MeJA-responsiveness
Hordeum vulgare	cis-acting regulatory element involved in the MeJA-responsiveness
Zea mays	part of a light responsive element
Avena sativa	light responsive element
Arabidopsis thaliana	cis-acting element involved in the abscisic acid responsiveness
Hordeum vulgare	cis-acting regulatory element involved in the MeJA-responsiveness
Pisum sativum	cis-acting regulatory element involved in light responsiveness
Zea mays	cis-acting regulatory element involved in zein metabolism regulation
Petroselinum crispum	part of a conserved DNA module involved in light responsiveness
Petroselinum crispum	part of a conserved DNA module involved in light responsiveness

Hordeum vulgare	cis-acting regulatory element involved in the MeJA-responsiveness
Arabidopsis thaliana	part of a module for light response
Spinacia oleracea	part of a light responsive element
Pisum sativum	part of a light responsive element
Oryza sativa	cis-acting element involved in gibberellin-responsiveness
Hordeum vulgare	cis-acting regulatory element involved in the MeJA-responsiveness
Hordeum vulgare	cis-acting regulatory element involved in the MeJA-responsiveness
Hordeum vulgare	cis-acting regulatory element involved in the MeJA-responsiveness
Solanum tuberosum	light responsive element
Spinacia oleracea	part of a light responsive element
Petroselinum crispum	MYB binding site involved in light responsiveness
Petroselinum crispum	MYB binding site involved in light responsiveness
Zea mays	cis-acting regulatory element involved in light responsiveness
Oryza sativa	cis-acting element involved in gibberellin-responsiveness
Arabidopsis thaliana	cis-acting element involved in the abscisic acid responsiveness
Arabidopsis thaliana	cis-acting element involved in the abscisic acid responsiveness
Petroselinum crispum	cis-acting element involved in light responsiveness
Avena sativa	light responsive element
Arabidopsis thaliana	light responsive element
Arabidopsis thaliana	light responsive element
Arabidopsis thaliana	light responsive element
Oryza sativa	gibberellin-responsive element
Arabidopsis thaliana	MYB binding site involved in drought-inducibility
Pisum sativum	cis-acting regulatory element involved in light responsiveness
Hordeum vulgare	cis-acting regulatory element involved in the MeJA-responsiveness
Hordeum vulgare	cis-acting regulatory element involved in the MeJA-responsiveness
Hordeum vulgare	cis-acting regulatory element involved in the MeJA-responsiveness
Petroselinum crispum	part of a conserved DNA module involved in light responsiveness
Petroselinum crispum	part of a conserved DNA module involved in light responsiveness
Zea mays	enhancer-like element involved in anoxic specific inducibility
Hordeum vulgare	cis-acting element involved in low-temperature responsiveness
Hordeum vulgare	cis-acting element involved in low-temperature responsiveness
Oryza sativa	cis-acting element involved in gibberellin-responsiveness
Daucus carota	part of a light responsive element
Daucus carota	part of a light responsive element
Zea mays	cis-acting regulatory element involved in light responsiveness
Pisum sativum	part of a light responsive element
Arabidopsis thaliana	part of a module for light response
Spinacia oleracea	part of a light responsive element
Spinacia oleracea	part of a light responsive element
Arabidopsis thaliana	part of a light responsive element
Hordeum vulgare	cis-acting regulatory element involved in the MeJA-responsiveness
Hordeum vulgare	cis-acting regulatory element involved in the MeJA-responsiveness
Petroselinum crispum	part of a conserved DNA module involved in light responsiveness
Petroselinum crispum	part of a conserved DNA module involved in light responsiveness
Petroselinum crispum	part of a conserved DNA module involved in light responsiveness

Petroselinum crispum	light responsive element
Arabidopsis thaliana	cis-acting regulatory element related to meristem expression
Hordeum vulgare	cis-acting regulatory element involved in the MeJA-responsiveness
Hordeum vulgare	cis-acting regulatory element involved in the MeJA-responsiveness
Pisum sativum	cis-acting regulatory element involved in light responsiveness
Pisum sativum	cis-acting regulatory element involved in light responsiveness
Oryza sativa	gibberellin-responsive element
Oryza sativa	gibberellin-responsive element
Arabidopsis thaliana	light responsive element
Arabidopsis thaliana	cis-acting element involved in the abscisic acid responsiveness
Arabidopsis thaliana	cis-acting element involved in the abscisic acid responsiveness
Arabidopsis thaliana	cis-acting element involved in the abscisic acid responsiveness
Petroselinum crispum	cis-acting element involved in light responsiveness
Arabidopsis thaliana	part of a module for light response
Arabidopsis thaliana	part of a light responsive element
Arabidopsis thaliana	part of a light responsive element
Zea mays	part of a light responsive element
Hordeum vulgare	cis-acting regulatory element involved in the MeJA-responsiveness
Hordeum vulgare	cis-acting regulatory element involved in the MeJA-responsiveness
Oryza sativa	cis-acting element involved in gibberellin-responsiveness
Brassica oleracea	cis-acting regulatory element involved in light responsiveness
Zea mays	cis-acting regulatory element involved in light responsiveness
Daucus carota	part of a light responsive element
Daucus carota	part of a light responsive element
Oryza sativa	gibberellin-responsive element
Oryza sativa	gibberellin-responsive element
Oryza sativa	gibberellin-responsive element
Arabidopsis thaliana	cis-acting element involved in the abscisic acid responsiveness
Arabidopsis thaliana	cis-acting element involved in the abscisic acid responsiveness
Arabidopsis thaliana	cis-acting element involved in the abscisic acid responsiveness
Arabidopsis thaliana	cis-acting element involved in the abscisic acid responsiveness
Arabidopsis thaliana	light responsive element
Arabidopsis thaliana	cis-acting regulatory element related to meristem expression
Arabidopsis thaliana	cis-acting regulatory element related to meristem expression
Zea mays	cis-acting regulatory element essential for the anaerobic induction
Petroselinum crispum	part of a conserved DNA module involved in light responsiveness
Petroselinum crispum	part of a conserved DNA module involved in light responsiveness
Petroselinum crispum	part of a conserved DNA module involved in light responsiveness
Pisum sativum	cis-acting regulatory element involved in light responsiveness
Pisum sativum	cis-acting regulatory element involved in light responsiveness
Hordeum vulgare	cis-acting regulatory element involved in the MeJA-responsiveness
Hordeum vulgare	cis-acting regulatory element involved in the MeJA-responsiveness
Hordeum vulgare	cis-acting regulatory element involved in the MeJA-responsiveness
Hordeum vulgare	cis-acting regulatory element involved in the MeJA-responsiveness
Hordeum vulgare	cis-acting regulatory element involved in the MeJA-responsiveness
Petroselinum crispum	cis-acting regulatory element

Spinacia oleracea	part of a light responsive element
Zea mays	cis-acting regulatory element involved in light responsiveness
Zea mays	cis-acting regulatory element involved in light responsiveness
Zea mays	cis-acting regulatory element involved in light responsiveness
Daucus carota	part of a light responsive element
Oryza sativa	cis-acting element involved in gibberellin-responsiveness
Arabidopsis thaliana	cis-acting element involved in the abscisic acid responsiveness
Arabidopsis thaliana	cis-acting element involved in the abscisic acid responsiveness
Arabidopsis thaliana	cis-acting element involved in the abscisic acid responsiveness
Arabidopsis thaliana	cis-acting element involved in the abscisic acid responsiveness
Arabidopsis thaliana	light responsive element
Nicotiana tabacum	cis-acting element involved in salicylic acid responsiveness
Nicotiana tabacum	cis-acting element involved in salicylic acid responsiveness
Oryza sativa	gibberellin-responsive element
Pisum sativum	cis-acting regulatory element involved in light responsiveness
Pisum sativum	cis-acting regulatory element involved in light responsiveness
Hordeum vulgare	cis-acting regulatory element involved in the MeJA-responsiveness
Hordeum vulgare	cis-acting regulatory element involved in the MeJA-responsiveness
Hordeum vulgare	cis-acting regulatory element involved in the MeJA-responsiveness
Arabidopsis thaliana	MYB binding site involved in drought-inducibility
Zea mays	cis-acting regulatory element involved in zein metabolism regulation
Arabidopsis thaliana	cis-acting regulatory element related to meristem expression
Arabidopsis thaliana	cis-acting regulatory element related to meristem expression
Petroselinum crispum	part of a conserved DNA module involved in light responsiveness
Petroselinum crispum	part of a conserved DNA module involved in light responsiveness
Petroselinum crispum	part of a conserved DNA module involved in light responsiveness
Arabidopsis thaliana	part of a light responsive element
Larix laricina	part of a light responsive element
Flaveria trinervia	part of a light responsive element
Brassica oleracea	auxin-responsive element
Brassica oleracea	auxin-responsive element
Glycine max	binding site of AT-rich DNA binding protein (ATBP-1)
Brassica oleracea	gibberellin-responsive element
Brassica oleracea	gibberellin-responsive element
Solanum tuberosum	part of a light responsive element
Arabidopsis thaliana	part of a light responsive element
Arabidopsis thaliana	part of a light responsive element
Arabidopsis thaliana	cis-acting regulatory element involved in light responsiveness
Zea mays	cis-acting regulatory element involved in light responsiveness
Nicotiana tabacum	cis-acting element involved in salicylic acid responsiveness
Arabidopsis thaliana	cis-acting element involved in the abscisic acid responsiveness
Arabidopsis thaliana	cis-acting element involved in the abscisic acid responsiveness
Petroselinum crispum	part of a conserved DNA module involved in light responsiveness
Arabidopsis thaliana	MYB binding site involved in drought-inducibility
Pisum sativum	part of a conserved DNA module involved in light responsiveness
Pisum sativum	part of a conserved DNA module involved in light responsiveness

Pisum sativum	part of a conserved DNA module involved in light responsiveness
Pisum sativum	part of a conserved DNA module involved in light responsiveness
Hordeum vulgare	cis-acting regulatory element involved in the MeJA-responsiveness
Pisum sativum	cis-acting regulatory element involved in light responsiveness
Pisum sativum	cis-acting regulatory element involved in light responsiveness
Petroselinum crispum	part of a conserved DNA module involved in light responsiveness
Petroselinum crispum	part of a conserved DNA module involved in light responsiveness
Petroselinum crispum	part of a conserved DNA module involved in light responsiveness
Arabidopsis thaliana	part of a light responsive element
Zea mays	cis-acting regulatory element involved in zein metabolism regulation
Solanum tuberosum	light responsive element
Arabidopsis thaliana	light responsive element
Arabidopsis thaliana	cis-acting element involved in the abscisic acid responsiveness
Arabidopsis thaliana	cis-acting element involved in the abscisic acid responsiveness
Arabidopsis thaliana	cis-acting element involved in the abscisic acid responsiveness
Brassica oleracea	auxin-responsive element
Zea mays	cis-acting regulatory element involved in light responsiveness
Pisum sativum	part of a conserved DNA module array (CMA3)
Oryza sativa	cis-acting element involved in gibberellin-responsiveness
Pisum sativum	cis-acting element involved in phytochrome down-regulation expression
Hordeum vulgare	cis-acting regulatory element involved in the MeJA-responsiveness
Arabidopsis thaliana	part of a light responsive element
Arabidopsis thaliana	part of a module for light response
Zea mays	cis-acting regulatory element involved in light responsiveness
Zea mays	cis-acting regulatory element involved in light responsiveness
Arabidopsis thaliana	cis-acting regulatory element involved in light responsiveness
Zea mays	cis-acting regulatory element involved in light responsiveness
Arabidopsis thaliana	cis-acting regulatory element involved in light responsiveness
Glycine max	part of an auxin-responsive element
Brassica oleracea	auxin-responsive element
Hordeum vulgare	cis-acting element involved in low-temperature responsiveness
Gossypium hirsutum	part of a light responsive element
Flaveria trinervia	part of a light responsive element
Hordeum vulgare	cis-acting regulatory element involved in the MeJA-responsiveness
Hordeum vulgare	cis-acting regulatory element involved in the MeJA-responsiveness
Hordeum vulgare	cis-acting regulatory element involved in the MeJA-responsiveness
Hordeum vulgare	cis-acting regulatory element involved in the MeJA-responsiveness
Hordeum vulgare	cis-acting regulatory element involved in the MeJA-responsiveness
Hordeum vulgare	cis-acting regulatory element involved in the MeJA-responsiveness
Helianthus annuus	cis-acting regulatory element involved in seed-specific regulation
Petroselinum crispum	cis-acting element involved in light responsiveness
Petroselinum crispum	cis-acting element involved in light responsiveness
Arabidopsis thaliana	cis-acting element involved in the abscisic acid responsiveness
Arabidopsis thaliana	cis-acting element involved in the abscisic acid responsiveness
Arabidopsis thaliana	cis-acting element involved in the abscisic acid responsiveness
Oryza sativa	cis-regulatory element involved in endosperm expression

Hordeum vulgare	cis-acting element involved in the abscisic acid responsiveness
Arabidopsis thaliana	cis-acting element involved in the abscisic acid responsiveness
Arabidopsis thaliana	cis-acting element involved in the abscisic acid responsiveness
Arabidopsis thaliana	cis-acting element involved in the abscisic acid responsiveness
Arabidopsis thaliana	cis-acting regulatory element related to meristem expression
Arabidopsis thaliana	part of a light responsive element
Arabidopsis thaliana	part of a light responsive element
Petroselinum crispum	part of a conserved DNA module involved in light responsiveness
Pisum sativum	cis-acting regulatory element involved in light responsiveness
Hordeum vulgare	cis-acting regulatory element involved in the MeJA-responsiveness
Pisum sativum	part of a conserved DNA module involved in light responsiveness
Nicotiana tabacum	cis-acting element involved in defense and stress responsiveness
Hordeum vulgare	cis-acting regulatory element involved in the MeJA-responsiveness
Arabidopsis thaliana	cis-acting regulatory element involved in light responsiveness
Arabidopsis thaliana	cis-acting regulatory element involved in light responsiveness
Arabidopsis thaliana	part of a light responsive element
Arabidopsis thaliana	part of a light responsive element
Zea mays	cis-acting regulatory element involved in light responsiveness
Petroselinum crispum	part of a light responsive element
Hordeum vulgare	cis-acting regulatory element involved in the MeJA-responsiveness
Hordeum vulgare	cis-acting regulatory element involved in the MeJA-responsiveness
Hordeum vulgare	cis-acting regulatory element involved in the MeJA-responsiveness
Hordeum vulgare	cis-acting regulatory element involved in the MeJA-responsiveness
Nicotiana tabacum	cis-acting element involved in defense and stress responsiveness
Nicotiana tabacum	cis-acting element involved in defense and stress responsiveness
Nicotiana tabacum	cis-acting element involved in defense and stress responsiveness
Nicotiana tabacum	cis-acting element involved in defense and stress responsiveness
Arabidopsis thaliana	MYB binding site involved in drought-inducibility
Pisum sativum	cis-acting regulatory element involved in light responsiveness
Hordeum vulgare	cis-acting regulatory element involved in the MeJA-responsiveness
Hordeum vulgare	cis-acting regulatory element involved in the MeJA-responsiveness
Hordeum vulgare	cis-acting regulatory element involved in the MeJA-responsiveness
Hordeum vulgare	cis-acting regulatory element involved in the MeJA-responsiveness
Arabidopsis thaliana	cis-acting regulatory element related to meristem expression
Arabidopsis thaliana	part of a light responsive element
Arabidopsis thaliana	part of a light responsive element
Petroselinum crispum	part of a conserved DNA module involved in light responsiveness
Arabidopsis thaliana	cis-acting element involved in the abscisic acid responsiveness
Arabidopsis thaliana	cis-acting element involved in the abscisic acid responsiveness
Oryza sativa	cis-regulatory element involved in endosperm expression
Hordeum vulgare	cis-acting regulatory element involved in the MeJA-responsiveness
Hordeum vulgare	cis-acting regulatory element involved in the MeJA-responsiveness
Hordeum vulgare	cis-acting regulatory element involved in the MeJA-responsiveness
Arabidopsis thaliana	cis-acting regulatory element involved in light responsiveness
Arabidopsis thaliana	cis-acting regulatory element involved in light responsiveness
Arabidopsis thaliana	cis-acting regulatory element involved in light responsiveness

Arabidopsis thaliana	light responsive element
Arabidopsis thaliana	light responsive element
Arabidopsis thaliana	cis-acting element involved in the abscisic acid responsiveness
Arabidopsis thaliana	cis-acting element involved in the abscisic acid responsiveness
Arabidopsis thaliana	cis-acting element involved in the abscisic acid responsiveness
Hordeum vulgare	part of a light responsive element
Petroselinum crispum	cis-acting regulatory element
Petroselinum crispum	MYB binding site involved in light responsiveness
Petroselinum crispum	MYB binding site involved in light responsiveness
Brassica oleracea	auxin-responsive element
Oryza sativa	cis-acting element involved in gibberellin-responsiveness
Nicotiana tabacum	cis-acting regulatory element involved in auxin responsiveness
Hordeum vulgare	cis-acting regulatory element involved in the MeJA-responsiveness
Hordeum vulgare	cis-acting regulatory element involved in the MeJA-responsiveness
Hordeum vulgare	cis-acting regulatory element involved in the MeJA-responsiveness
Petroselinum crispum	part of a conserved DNA module involved in light responsiveness
Petroselinum crispum	part of a conserved DNA module involved in light responsiveness
Arabidopsis thaliana	cis-acting regulatory element related to meristem expression
Arabidopsis thaliana	part of a light responsive element
Arabidopsis thaliana	part of a light responsive element
Hordeum vulgare	cis-acting regulatory element involved in the MeJA-responsiveness
Zea mays	part of a light responsive element
Nicotiana tabacum	cis-acting element involved in defense and stress responsiveness
Brassica oleracea	cis-acting regulatory element involved in light responsiveness
Brassica oleracea	cis-acting regulatory element involved in light responsiveness
Brassica oleracea	cis-acting regulatory element involved in light responsiveness
Avena sativa	light responsive element
Brassica oleracea	cis-acting element involved in salicylic acid responsiveness
Hordeum vulgare	cis-acting regulatory element involved in the MeJA-responsiveness
Arabidopsis thaliana	cis-acting regulatory element related to meristem expression
Petroselinum crispum	part of a conserved DNA module involved in light responsiveness
Petroselinum crispum	part of a conserved DNA module involved in light responsiveness
Petroselinum crispum	part of a conserved DNA module involved in light responsiveness
Hordeum vulgare	cis-acting element involved in the abscisic acid responsiveness
Arabidopsis thaliana	cis-acting element involved in the abscisic acid responsiveness
Arabidopsis thaliana	cis-acting element involved in the abscisic acid responsiveness
Arabidopsis thaliana	cis-acting element involved in the abscisic acid responsiveness
Oryza sativa	gibberellin-responsive element
Pisum sativum	cis-acting regulatory element involved in light responsiveness
Pisum sativum	cis-acting regulatory element involved in light responsiveness
Arabidopsis thaliana	element involved in differentiation of the palisade mesophyll cells
Arabidopsis thaliana	cis-acting regulatory element related to meristem expression
Petroselinum crispum	part of a conserved DNA module involved in light responsiveness
Petroselinum crispum	part of a conserved DNA module involved in light responsiveness
Petroselinum crispum	part of a conserved DNA module involved in light responsiveness
Petroselinum crispum	part of a conserved DNA module involved in light responsiveness

Petroselinum crispum	part of a conserved DNA module involved in light responsiveness
Petroselinum crispum	cis-acting regulatory element
Arabidopsis thaliana	cis-acting regulatory element involved in light responsiveness
Hordeum vulgare	cis-acting regulatory element involved in the MeJA-responsiveness
Nicotiana tabacum	cis-acting element involved in defense and stress responsiveness
Brassica oleracea	auxin-responsive element
Oryza sativa	cis-regulatory element involved in endosperm expression
Arabidopsis thaliana	light responsive element
Arabidopsis thaliana	light responsive element
Avena sativa	light responsive element
Arabidopsis thaliana	light responsive element
Avena sativa	light responsive element
Avena sativa	light responsive element
Nicotiana tabacum	cis-acting regulatory element involved in auxin responsiveness
Arabidopsis thaliana	cis-acting element involved in the abscisic acid responsiveness
Arabidopsis thaliana	cis-acting element involved in the abscisic acid responsiveness
Petroselinum crispum	part of a conserved DNA module involved in light responsiveness
Arabidopsis thaliana	cis-acting regulatory element related to meristem expression
Pisum sativum	part of a conserved DNA module involved in light responsiveness
Hordeum vulgare	cis-acting regulatory element involved in the MeJA-responsiveness
Pisum sativum	cis-acting regulatory element involved in light responsiveness
Brassica oleracea	auxin-responsive element
Arabidopsis thaliana	cis-acting regulatory element involved in light responsiveness
Zea mays	cis-acting regulatory element involved in light responsiveness
Zea mays	cis-acting regulatory element involved in light responsiveness
Arabidopsis thaliana	part of a module for light response
Hordeum vulgare	cis-acting regulatory element involved in the MeJA-responsiveness
Hordeum vulgare	cis-acting regulatory element involved in the MeJA-responsiveness
Hordeum vulgare	cis-acting regulatory element involved in the MeJA-responsiveness
Petroselinum crispum	part of a conserved DNA module involved in light responsiveness
Petroselinum crispum	part of a conserved DNA module involved in light responsiveness
Petroselinum crispum	part of a conserved DNA module involved in light responsiveness
Petroselinum crispum	part of a conserved DNA module involved in light responsiveness
Pisum sativum	cis-acting regulatory element involved in light responsiveness
Pisum sativum	cis-acting regulatory element involved in light responsiveness
Hordeum vulgare	cis-acting regulatory element involved in the MeJA-responsiveness
Hordeum vulgare	cis-acting regulatory element involved in the MeJA-responsiveness
Hordeum vulgare	cis-acting regulatory element involved in the MeJA-responsiveness
Arabidopsis thaliana	cis-acting element involved in the abscisic acid responsiveness
Arabidopsis thaliana	cis-acting element involved in the abscisic acid responsiveness
Arabidopsis thaliana	cis-acting element involved in the abscisic acid responsiveness
Arabidopsis thaliana	cis-acting element involved in the abscisic acid responsiveness
Arabidopsis thaliana	cis-acting element involved in the abscisic acid responsiveness
Arabidopsis thaliana	light responsive element
Nicotiana tabacum	cis-acting element involved in salicylic acid responsiveness
Oryza sativa	gibberellin-responsive element

Arabidopsis thaliana	cis-acting element involved in the abscisic acid responsiveness
Arabidopsis thaliana	part of a light responsive element
Petroselinum crispun	part of a conserved DNA module involved in light responsiveness
Petroselinum crispun	part of a conserved DNA module involved in light responsiveness
Petroselinum crispun	part of a conserved DNA module involved in light responsiveness
Petroselinum crispun	part of a conserved DNA module involved in light responsiveness
Petroselinum crispun	part of a conserved DNA module involved in light responsiveness
Pisum sativum	cis-acting regulatory element involved in light responsiveness
Hordeum vulgare	cis-acting regulatory element involved in the MeJA-responsiveness
Hordeum vulgare	cis-acting regulatory element involved in the MeJA-responsiveness
Brassica oleracea	auxin-responsive element
Petroselinum crispun	MYB binding site involved in light responsiveness
Nicotiana tabacum	cis-acting regulatory element involved in auxin responsiveness
Arabidopsis thaliana	light responsive element
Petroselinum crispun	cis-acting element involved in light responsiveness
Arabidopsis thaliana	cis-acting element involved in the abscisic acid responsiveness
Arabidopsis thaliana	cis-acting element involved in the abscisic acid responsiveness
Oryza sativa	cis-regulatory element involved in endosperm expression
Oryza sativa	light responsive element
Oryza sativa	gibberellin-responsive element
Hordeum vulgare	cis-acting regulatory element involved in the MeJA-responsiveness
Hordeum vulgare	cis-acting regulatory element involved in the MeJA-responsiveness
Hordeum vulgare	cis-acting regulatory element involved in the MeJA-responsiveness
Hordeum vulgare	cis-acting regulatory element involved in the MeJA-responsiveness
Hordeum vulgare	cis-acting regulatory element involved in the MeJA-responsiveness
Pisum sativum	cis-acting regulatory element involved in light responsiveness
Petroselinum crispun	part of a conserved DNA module involved in light responsiveness
Petroselinum crispun	part of a conserved DNA module involved in light responsiveness
Petroselinum crispun	part of a conserved DNA module involved in light responsiveness
Petroselinum crispun	part of a conserved DNA module involved in light responsiveness
Petroselinum crispun	part of a conserved DNA module involved in light responsiveness
Hordeum vulgare	cis-acting regulatory element involved in the MeJA-responsiveness
Hordeum vulgare	cis-acting regulatory element involved in the MeJA-responsiveness
Hordeum vulgare	cis-acting regulatory element involved in the MeJA-responsiveness
Hordeum vulgare	cis-acting regulatory element involved in the MeJA-responsiveness
Hordeum vulgare	cis-acting regulatory element involved in the MeJA-responsiveness
Arabidopsis thaliana	cis-acting regulatory element involved in light responsiveness
Solanum tuberosum	part of a light responsive element
Oryza sativa	cis-acting element involved in gibberellin-responsiveness
Hordeum vulgare	cis-acting regulatory element involved in the MeJA-responsiveness
Hordeum vulgare	cis-acting regulatory element involved in the MeJA-responsiveness
Arabidopsis thaliana	part of a light responsive element
Brassica oleracea	cis-acting regulatory element involved in light responsiveness
Zea mays	cis-acting regulatory element involved in light responsiveness
Glycine max	part of an auxin-responsive element
Petroselinum crispun	part of a light responsive element

Hordeum vulgare	cis-acting regulatory element involved in the MeJA-responsiveness
Hordeum vulgare	cis-acting regulatory element involved in the MeJA-responsiveness
Arabidopsis thaliana	part of a light responsive element
Arabidopsis thaliana	part of a light responsive element
Arabidopsis thaliana	part of a light responsive element
Arabidopsis thaliana	cis-acting regulatory element related to meristem expression
Arabidopsis thaliana	cis-acting regulatory element related to meristem expression
Petroselinum crispun	part of a conserved DNA module involved in light responsiveness
Petroselinum crispun	part of a conserved DNA module involved in light responsiveness
Petroselinum crispun	part of a conserved DNA module involved in light responsiveness
Arabidopsis thaliana	light responsive element
Arabidopsis thaliana	cis-acting element involved in the abscisic acid responsiveness
Hordeum vulgare	cis-acting element involved in the abscisic acid responsiveness
Arabidopsis thaliana	cis-acting element involved in the abscisic acid responsiveness
Hordeum vulgare	cis-acting element involved in the abscisic acid responsiveness
Arabidopsis thaliana	cis-acting element involved in the abscisic acid responsiveness
Arabidopsis thaliana	cis-acting element involved in the abscisic acid responsiveness
Glycine max	part of an auxin-responsive element
Nicotiana tabacum	cis-acting element involved in salicylic acid responsiveness
Brassica oleracea	cis-acting element involved in salicylic acid responsiveness
Hordeum vulgare	cis-acting regulatory element involved in the MeJA-responsiveness
Hordeum vulgare	cis-acting regulatory element involved in the MeJA-responsiveness
Hordeum vulgare	cis-acting regulatory element involved in the MeJA-responsiveness
Hordeum vulgare	cis-acting regulatory element involved in the MeJA-responsiveness
Pisum sativum	cis-acting regulatory element involved in light responsiveness
Pisum sativum	cis-acting regulatory element involved in light responsiveness
Arabidopsis thaliana	part of a light responsive element
Hordeum vulgare	cis-acting regulatory element involved in the MeJA-responsiveness
Hordeum vulgare	cis-acting regulatory element involved in the MeJA-responsiveness
Hordeum vulgare	cis-acting regulatory element involved in the MeJA-responsiveness
Hordeum vulgare	cis-acting regulatory element involved in the MeJA-responsiveness
Arabidopsis thaliana	cis-acting regulatory element involved in light responsiveness
Brassica oleracea	cis-acting regulatory element involved in light responsiveness
Zea mays	cis-acting regulatory element involved in light responsiveness
Petroselinum crispun	part of a conserved DNA module involved in light responsiveness
Petroselinum crispun	part of a conserved DNA module involved in light responsiveness
Petroselinum crispun	part of a conserved DNA module involved in light responsiveness
Petroselinum crispun	part of a conserved DNA module involved in light responsiveness
Petroselinum crispun	part of a conserved DNA module involved in light responsiveness
Petroselinum crispun	part of a conserved DNA module involved in light responsiveness
Pisum sativum	cis-acting regulatory element involved in light responsiveness
Hordeum vulgare	cis-acting regulatory element involved in the MeJA-responsiveness
Hordeum vulgare	cis-acting regulatory element involved in the MeJA-responsiveness
Nicotiana tabacum	cis-acting element involved in salicylic acid responsiveness
Arabidopsis thaliana	cis-acting element involved in the abscisic acid responsiveness
Arabidopsis thaliana	cis-acting element involved in the abscisic acid responsiveness

Avena sativa	light responsive element
Solanum tuberosum	light responsive element
Arabidopsis thaliana	cis-acting regulatory element involved in light responsiveness
Brassica oleracea	auxin-responsive element
Nicotiana tabacum	cis-acting element involved in defense and stress responsiveness
Hordeum vulgare	cis-acting regulatory element involved in the MeJA-responsiveness
Hordeum vulgare	cis-acting regulatory element involved in the MeJA-responsiveness
Solanum tuberosum	light responsive element
Arabidopsis thaliana	light responsive element
Hordeum vulgare	cis-acting regulatory element involved in the MeJA-responsiveness
Spinacia oleracea	part of a light responsive element
Nicotiana tabacum	cis-acting element involved in defense and stress responsiveness
Hordeum vulgare	cis-acting regulatory element involved in the MeJA-responsiveness
Arabidopsis thaliana	part of a light responsive element
Hordeum vulgare	cis-acting regulatory element involved in the MeJA-responsiveness
Pisum sativum	cis-acting regulatory element involved in light responsiveness
Oryza sativa	gibberellin-responsive element
Oryza sativa	cis-regulatory element involved in endosperm expression
Arabidopsis thaliana	light responsive element
Arabidopsis thaliana	cis-acting element involved in the abscisic acid responsiveness
Triticum aestivum	cis-acting element involved in the abscisic acid responsiveness
Arabidopsis thaliana	cis-acting element involved in the abscisic acid responsiveness
Arabidopsis thaliana	cis-acting element involved in the abscisic acid responsiveness
Solanum tuberosum	part of a light responsive element
Zea mays	cis-acting regulatory element involved in light responsiveness
Brassica napus	cis-acting regulatory element involved in light responsiveness
Arabidopsis thaliana	cis-acting regulatory element involved in light responsiveness
Spinacia oleracea	part of a light responsive element
Arabidopsis thaliana	part of a module for light response
Petroselinum crispum	cis-acting regulatory element
Hordeum vulgare	cis-acting regulatory element involved in the MeJA-responsiveness
Larix laricina	part of a light responsive element
Spinacia oleracea	part of a light responsive element
Arabidopsis thaliana	part of a light responsive element
Nicotiana tabacum	cis-acting element involved in defense and stress responsiveness
Nicotiana tabacum	cis-acting element involved in defense and stress responsiveness
Brassica oleracea	auxin-responsive element
Petroselinum crispum	part of a conserved DNA module involved in light responsiveness
Arabidopsis thaliana	part of a light responsive element
Arabidopsis thaliana	cis-acting regulatory element related to meristem expression
Arabidopsis thaliana	MYB binding site involved in drought-inducibility
Hordeum vulgare	cis-acting regulatory element involved in the MeJA-responsiveness
Hordeum vulgare	cis-acting regulatory element involved in the MeJA-responsiveness
Hordeum vulgare	cis-acting regulatory element involved in the MeJA-responsiveness
Hordeum vulgare	cis-acting regulatory element involved in the MeJA-responsiveness
Hordeum vulgare	cis-acting regulatory element involved in the MeJA-responsiveness

Hordeum vulgare	cis-acting regulatory element involved in the MeJA-responsiveness
Solanum tuberosum	part of a light responsive element
Brassica oleracea	gibberellin-responsive element
Zea mays	cis-acting regulatory element involved in light responsiveness
Zea mays	cis-acting regulatory element involved in light responsiveness
Brassica napus	cis-acting regulatory element involved in light responsiveness
Arabidopsis thaliana	cis-acting regulatory element involved in light responsiveness
Oryza sativa	gibberellin-responsive element
Oryza sativa	gibberellin-responsive element
Nicotiana tabacum	cis-acting element involved in salicylic acid responsiveness
Nicotiana tabacum	cis-acting element involved in salicylic acid responsiveness
Nicotiana tabacum	cis-acting element involved in salicylic acid responsiveness
Arabidopsis thaliana	light responsive element
Arabidopsis thaliana	light responsive element
Arabidopsis thaliana	cis-acting element involved in the abscisic acid responsiveness
Arabidopsis thaliana	cis-acting element involved in the abscisic acid responsiveness
Triticum aestivum	cis-acting element involved in the abscisic acid responsiveness
Arabidopsis thaliana	cis-acting element involved in the abscisic acid responsiveness
Arabidopsis thaliana	cis-acting element involved in the abscisic acid responsiveness
Arabidopsis thaliana	element involved in differentiation of the palisade mesophyll cells
Pisum sativum	cis-acting regulatory element involved in light responsiveness
Oryza sativa	light responsive element
Oryza sativa	cis-regulatory element involved in endosperm expression
Pisum sativum	part of a light responsive element
Arabidopsis thaliana	cis-acting element involved in the abscisic acid responsiveness
Avena sativa	light responsive element
Solanum tuberosum	light responsive element
Arabidopsis thaliana	light responsive element
Arabidopsis thaliana	part of a light responsive element
Arabidopsis thaliana	cis-acting regulatory element related to meristem expression
Arabidopsis thaliana	cis-acting regulatory element related to meristem expression
Arabidopsis thaliana	cis-acting regulatory element related to meristem expression
Petroselinum crispum	part of a conserved DNA module involved in light responsiveness
Hordeum vulgare	cis-acting regulatory element involved in the MeJA-responsiveness
Hordeum vulgare	cis-acting regulatory element involved in the MeJA-responsiveness
Brassica oleracea	auxin-responsive element
Solanum tuberosum	part of a light responsive element
Arabidopsis thaliana	part of a light responsive element
Petroselinum crispum	MYB binding site involved in light responsiveness
Zea mays	cis-acting regulatory element involved in light responsiveness
Arabidopsis thaliana	cis-acting regulatory element involved in light responsiveness
Spinacia oleracea	part of a conserved DNA module involved in light responsiveness
Spinacia oleracea	part of a conserved DNA module involved in light responsiveness
Hordeum vulgare	cis-acting regulatory element involved in the MeJA-responsiveness
Hordeum vulgare	cis-acting regulatory element involved in the MeJA-responsiveness
Hordeum vulgare	cis-acting regulatory element involved in the MeJA-responsiveness

Hordeum vulgare	cis-acting regulatory element involved in the MeJA-responsiveness
Arabidopsis thaliana	cis-acting regulatory element related to meristem expression
Arabidopsis thaliana	cis-acting regulatory element related to meristem expression
Arabidopsis thaliana	cis-acting regulatory element related to meristem expression
Petroselinum crispun	part of a conserved DNA module involved in light responsiveness
Petroselinum crispun	part of a conserved DNA module involved in light responsiveness
Hordeum vulgare	cis-acting element involved in the abscisic acid responsiveness
Arabidopsis thaliana	cis-acting element involved in the abscisic acid responsiveness
Avena sativa	light responsive element
Arabidopsis thaliana	light responsive element
Avena sativa	light responsive element
Arabidopsis thaliana	light responsive element
Petroselinum crispun	part of a conserved DNA module involved in light responsiveness
Petroselinum crispun	part of a conserved DNA module involved in light responsiveness
Arabidopsis thaliana	part of a light responsive element
Hordeum vulgare	cis-acting regulatory element involved in the MeJA-responsiveness
Oryza sativa	light responsive element
Petroselinum crispun	cis-acting element involved in light responsiveness
Petroselinum crispun	MYB binding site involved in light responsiveness
Spinacia oleracea	part of a conserved DNA module involved in light responsiveness
Petroselinum crispun	cis-acting element involved in light responsiveness
Oryza sativa	cis-acting element involved in gibberellin-responsiveness
Petroselinum crispun	MYB binding site involved in light responsiveness
Pisum sativum	cis-acting regulatory element involved in light responsiveness
Oryza sativa	cis-regulatory element involved in endosperm expression
Oryza sativa	involved in endosperm-specific negative expression
Oryza sativa	involved in endosperm-specific negative expression
Oryza sativa	gibberellin-responsive element
Arabidopsis thaliana	cis-acting element involved in the abscisic acid responsiveness
Arabidopsis thaliana	light responsive element
Arabidopsis thaliana	light responsive element
Arabidopsis thaliana	light responsive element
Zea mays	cis-acting regulatory element involved in light responsiveness
Hordeum vulgare	cis-acting regulatory element involved in the MeJA-responsiveness
Hordeum vulgare	cis-acting regulatory element involved in the MeJA-responsiveness
Pisum sativum	cis-acting regulatory element involved in light responsiveness
Pisum sativum	cis-acting regulatory element involved in light responsiveness
Hordeum vulgare	cis-acting regulatory element involved in the MeJA-responsiveness
Pisum sativum	part of a conserved DNA module involved in light responsiveness
Petroselinum crispun	part of a conserved DNA module involved in light responsiveness
Helianthus annuus	cis-acting regulatory element involved in seed-specific regulation
Arabidopsis thaliana	cis-acting element involved in the abscisic acid responsiveness
Arabidopsis thaliana	cis-acting element involved in the abscisic acid responsiveness
Arabidopsis thaliana	cis-acting element involved in the abscisic acid responsiveness
Arabidopsis thaliana	cis-acting element involved in the abscisic acid responsiveness
Avena sativa	light responsive element

Arabidopsis thaliana	light responsive element
Nicotiana tabacum	cis-acting element involved in salicylic acid responsiveness
Oryza sativa	light responsive element
Arabidopsis thaliana	cis-acting regulatory element involved in light responsiveness
Arabidopsis thaliana	cis-acting regulatory element involved in light responsiveness
Brassica oleracea	auxin-responsive element
Brassica oleracea	auxin-responsive element
Hordeum vulgare	cis-acting regulatory element involved in the MeJA-responsiveness
Solanum tuberosum	light responsive element
Oryza sativa	cis-acting element involved in gibberellin-responsiveness
Brassica oleracea	auxin-responsive element
Zea mays	cis-acting regulatory element involved in light responsiveness
Petroselinum crispun	cis-acting regulatory element
Petroselinum crispun	cis-acting regulatory element
Hordeum vulgare	cis-acting regulatory element involved in the MeJA-responsiveness
Hordeum vulgare	cis-acting regulatory element involved in the MeJA-responsiveness
Larix laricina	part of a light responsive element
Arabidopsis thaliana	part of a light responsive element
Hordeum vulgare	cis-acting regulatory element involved in the MeJA-responsiveness
Hordeum vulgare	cis-acting regulatory element involved in the MeJA-responsiveness
Nicotiana tabacum	cis-acting element involved in salicylic acid responsiveness
Brassica oleracea	cis-acting element involved in salicylic acid responsiveness
Brassica oleracea	cis-acting element involved in salicylic acid responsiveness
Petroselinum crispun	cis-acting element involved in light responsiveness
Arabidopsis thaliana	cis-acting element involved in the abscisic acid responsiveness
Nicotiana tabacum	cis-acting regulatory element involved in auxin responsiveness
Arabidopsis thaliana	light responsive element
