# Supplementary material for: Genome-Wide Identification of Expansin Gene Family and Their Response under Hormone Exposure in Ginkgo biloba L
Source: Int J Mol Sci. 2023 Mar 21;24(6):5901. doi: 10.3390/ijms24065901 (PMC10053239; doi:10.3390/ijms24065901)
Supplement: Supplementary file 1 [file ijms-24-05901-s001.zip › Table S4.GO annotaion of GbEXPs.pdf]

Gene name	Description		Biologic[illegible]	
	Estimated PPV, description		Estimated PPV, [illegible]	
GBEXPA1	0.69	Expansin	0.83	GO:0009664
GBEXPA2	0.66	Expansin	0.83	GO:0009664
GBEXPA3	0.67	Expansin	0.83	GO:0009664
			0.36	GO:0006949
			0.36	GO:0009635
GBEXPA4	0.66	Expansin	0.83	GO:0009664
			0.36	GO:0006949
			0.36	GO:0009635
GBEXPA5	0.68	Expansin	0.83	GO:0009664
			0.38	GO:0006949
			0.36	GO:0010114
			0.36	GO:0010119
GBEXPA6	0.67	Expansin	0.83	GO:0009664
GBEXPA7	0.68	Expansin	0.83	GO:0009664
GBEXPA8	0.67	Expansin	0.83	GO:0009664
GBEXPA9	0.64	Expansin	0.83	GO:0009664
			0.39	GO:0006949
			0.38	GO:0009635
GBEXPA10	0.68	Expansin	0.83	GO:0009664
GBEXPA11	0.67	Expansin	0.83	GO:0009664
GBEXPB1	0.88	Expansin-B16 like		
GBEXPA20	0.66	Expansin	0.83	GO:0009664
			0.37	GO:0010311
GBEXPA21	0.67	Expansin	0.83	GO:0009664

GBEXLB1	0.81 Putative expansin/Lol pI		
GBEXLA1	0.82 Beta-expansin		
GBEXLA2	0.79 Beta-expansin		
GBEXLB2	0.85 Major pollen allergen Lol pI		
GBEXLB3	0.89 Major pollen allergen Lol pI		
GBEXPA12	0.67 Expansin	0.83	GO:0009664
GBEXPA13	0.66 Expansin	0.83	GO:0009664
GBEXPA14	0.67 Expansin	0.83	GO:0009664
		0.41	GO:0009826
		0.4	GO:0090376
		0.37	GO:0000904
		0.37	GO:0080022
		0.36	GO:0009834
		0.36	GO:0042545
GBEXLB4	0.2 Major pollen allergen Ory s 1		
GBEXLB5	0.76 Major pollen allergen Lol pI		
GBEXPA15	0.67 Expansin	0.83	GO:0009664
GBEXLA3	0.66 Major pollen allergen Ory s 1		

GBEXLA4	0.65 Major pollen allergen Lol pI		
GBEXLA5	0.73 Major pollen allergen Lol pI		
GBEXPA16	0.68 Expansin	0.83	GO:0009664
		0.38	GO:0006949
		0.38	GO:0009635
GBEXPA17	0.67 Expansin	0.83	GO:0009664
GBEXPA18	0.65 Expansin	0.83	GO:0009664
GBEXPA19	0.69 Expansin	0.83	GO:0009664
GBEXPB2	0.87 Expansin-B16 like		
GBEXPB3	0.85 Expansin-B16 like		
GBEXPB4	0.86 Expansin-B16 like		
GBEXPA22	0.64 Expansin	0.83	GO:0009664
		0.43	GO:0080022
		0.42	GO:0009826
GBEXPA23	0.67 Expansin	0.83	GO:0009664
GBEXPA24	0.67 Expansin	0.82	GO:0009664
		0.36	GO:0006949
		0.36	GO:0042545
GBEXPA25	0.66 Expansin	0.83	GO:0009664
		0.36	GO:0006949
		0.36	GO:0009635
GBEXPA26	0.68 Expansin	0.83	GO:0009664
GBEXPA27	0.69 Expansin	0.83	GO:0009664

GBEXPA28	0.68	Expansin	0.83	GO:0009664
GBEXPA29	0.66	Expansin	0.83	GO:0009664
			0.36	GO:0006949
			0.36	GO:0009635
GBEXPA30	0.67	Expansin	0.83	GO:0009664
GBEXPA31	0.66	Expansin	0.83	GO:0009664
GBEXPA32	0.65	Expansin	0.83	GO:0009664

al process	Molecular function	Cellular component		
GO-id, description	Estimated PPV, GO-id, description	Estimated PPV, GO-id, description		
plant-type cell wall organization		0.65	GO:0005576	extracellular region
		0.43	GO:0016020	membrane
plant-type cell wall organization		0.65	GO:0005576	extracellular region
		0.43	GO:0016020	membrane
plant-type cell wall organization		0.65	GO:0005576	extracellular region
syncytium formation<br>response to herbicide		0.43	GO:0016020	membrane
plant-type cell wall organization		0.65	GO:0005576	extracellular region
syncytium formation<br>response to herbicide		0.43	GO:0016020	membrane
plant-type cell wall organization<br>syncytium formation		0.65	GO:0005576	extracellular region
response to red light<br>regulation of stomatal movement		0.43	GO:0016020	membrane
plant-type cell wall organization		0.65	GO:0005576	extracellular region
		0.43	GO:0016020	membrane
plant-type cell wall organization		0.65	GO:0005576	extracellular region
		0.43	GO:0016020	membrane
plant-type cell wall organization		0.65	GO:0005576	extracellular region
		0.43	GO:0016020	membrane
plant-type cell wall organization		0.65	GO:0005576	extracellular region
syncytium formation<br>response to herbicide		0.43	GO:0016020	membrane
plant-type cell wall organization		0.65	GO:0005576	extracellular region
		0.43	GO:0016020	membrane
plant-type cell wall organization		0.65	GO:0005576	extracellular region
		0.43	GO:0016020	membrane
plant-type cell wall organization		0.65	GO:0005576	extracellular region
lateral root formation		0.43	GO:0016020	membrane
plant-type cell wall organization		0.65	GO:0005576	extracellular region
		0.43	GO:0016020	membrane

	0.65	GO:0005576	extracellular region
	0.32	GO:0016021	integral component of membrane
	0.65	GO:0005576	extracellular region
	0.65	GO:0005576	extracellular region
	0.65	GO:0005576	extracellular region
	0.32	GO:0016021	integral component of membrane
	0.65	GO:0005576	extracellular region
	0.32	GO:0016021	integral component of membrane
plant-type cell wall organization	0.65<br>0.43	GO:0005576<br>GO:0016020	extracellular region<br>membrane
plant-type cell wall organization	0.65<br>0.43	GO:0005576<br>GO:0016020	extracellular region<br>membrane
plant-type cell wall organization	0.65<br>0.43	GO:0005576<br>GO:0016020	extracellular region<br>membrane
unidimensional cell growth<br>seed trichome differentiation<br>cell morphogenesis involved in differentiation<br>primary root development<br>plant-type secondary cell wall biogenesis<br>cell wall modification	0.36	GO:0005618	cell wall
	0.65	GO:0005576	extracellular region
	0.65	GO:0005576	extracellular region
plant-type cell wall organization	0.65<br>0.43	GO:0005576<br>GO:0016020	extracellular region<br>membrane
	0.65	GO:0005576	extracellular region
	0.65	GO:0005576	extracellular region

	0.34	GO:0016021	integral component of membrane
	0.65	GO:0005576	extracellular region
	0.34	GO:0016021	integral component of membrane
plant-type cell wall organization	0.65	GO:0005576	extracellular region
syncytium formation<br>response to herbicide	0.43	GO:0016020	membrane
plant-type cell wall organization	0.65	GO:0005576	extracellular region
	0.43	GO:0016020	membrane
plant-type cell wall organization	0.65	GO:0005576	extracellular region
	0.43	GO:0016020	membrane
plant-type cell wall organization	0.65	GO:0005576	extracellular region
	0.43	GO:0016020	membrane
plant-type cell wall organization	0.65	GO:0005576	extracellular region
primary root development<br>unidimensional cell growth	0.43	GO:0016020	membrane
plant-type cell wall organization	0.65	GO:0005576	extracellular region
	0.43	GO:0016020	membrane
plant-type cell wall organization	0.65	GO:0005576	extracellular region
syncytium formation<br>cell wall modification	0.43	GO:0016020	membrane
plant-type cell wall organization	0.65	GO:0005576	extracellular region
syncytium formation<br>response to herbicide	0.43	GO:0016020	membrane
plant-type cell wall organization	0.65	GO:0005576	extracellular region
plant-type cell wall [illegible]	0.65	GO:0005576	extracellular region

organization	0.43	GO:0016020	membrane
plant-type cell wall organization	0.65	GO:0005576	extracellular region
	0.43	GO:0016020	membrane
plant-type cell wall organization	0.65	GO:0005576	extracellular region
syncytium formation<br>response to herbicide	0.43	GO:0016020	membrane
plant-type cell wall organization	0.65	GO:0005576	extracellular region
	0.43	GO:0016020	membrane
plant-type cell wall organization	0.65	GO:0005576	extracellular region
	0.43	GO:0016020	membrane
plant-type cell wall organization	0.65	GO:0005576	extracellular region
	0.43	GO:0016020	membrane
